# Optimizing lipid-lowering therapy for acute coronary syndrome using a decision support system: insights from a cluster randomized trial

**DOI:** 10.1093/ehjdh/ztaf135

**Published:** 2025-11-17

**Authors:** Christophe A T Stevens, Jessica Smith, Julia Brandts, Fotios Barkas, Maria Moreno Morales, Leila Janani, Gaia Kiru, Nandita Kaza, Victoria Cornelius, Neil R Poulter, Kamlesh Khunti, John William McEvoy, Alberto Zambon, Jose L Lopez-sendon, Derek Connolly, Lorna Hazell, Kausik K Ray, the ZODIAC trial, Kausik K Ray, Victoria Cornelius, Neil R Poulter, Gaia Kiru, Leila Janani, Jessica Smith, Christophe A T Stevens, Maria Moreno Morales, Annie Wright, Eloise Britten, Felicia Frost, Francesco Lala, Jonathan Dao, Karen Danois, Lisa Feng, Lorna Hazell, Nandita Kaza, Nayan Das, Safa Anwar, Sima Toopchiani, Smita Das, Stephanie Amiridis, Vanaja Kakarla, Yasmin Abdat, Derek Connolly, Kamlesh Khunti, Vinoda Sharma, Christina Elorz, Andrew Moriarty, Afzar Zaman, Satheesh Balakrishnan-Nair, Rasha Al-Lamee, Azeem Sheikh, Mohamed Alama, George Hunter, David Ripley, Vivek Kodoth, Daniel McKenzie, Joe Martins, Rashed Hossain, Sharad Agrawal, Helen Routledge, Sukhbir Dhamrait, Alberto Zambon, Natale Brunetti, Alessandro Navazio, Gianni Casella, Paolo Calabrò, Giuseppe Andò, Claudio Fresco, Roberta Della Bona, Andrea Borin, Raffaele De Caterina, Alessandro Sciahbasi, Giuseppe Boriani, Jesús Peteiro, Jose Ramón González Juanatey, José Tuñón, Manuel Martínez Sellés, Leire Unzué, Carlos Arellano, Jose Lopez Aguilera, Alessandro Sionis, Roberto Martín, Raul Moreno, Alejandro Villanueva, Jordi Lozano, Domingo Pascual, Francisco Javier Cortés, Julia Brandts

**Affiliations:** Department of Primary Care and Public Health, School of Public Health, Imperial College London, 90 Wood Lane, London W12 0BZ, UK; Imperial Clinical Trials Unit, School of Public Health, Imperial College London, 68 Wood Lane, London W12 7RH, UK; Department of Primary Care and Public Health, School of Public Health, Imperial College London, 90 Wood Lane, London W12 0BZ, UK; Department of Medicine, RWTH University Hospital Aachen, Pauwelsstraße 30, Aachen 52074, Germany; Department of Primary Care and Public Health, School of Public Health, Imperial College London, 90 Wood Lane, London W12 0BZ, UK; Faculty of Medicine, School of Health Sciences, University of Ioannina, P.O. Box 1186, Ioannina GR-45500, Greece; Imperial Clinical Trials Unit, School of Public Health, Imperial College London, 68 Wood Lane, London W12 7RH, UK; Imperial Clinical Trials Unit, School of Public Health, Imperial College London, 68 Wood Lane, London W12 7RH, UK; Imperial Clinical Trials Unit, School of Public Health, Imperial College London, 68 Wood Lane, London W12 7RH, UK; Imperial Clinical Trials Unit, School of Public Health, Imperial College London, 68 Wood Lane, London W12 7RH, UK; Imperial Clinical Trials Unit, School of Public Health, Imperial College London, 68 Wood Lane, London W12 7RH, UK; Imperial Clinical Trials Unit, School of Public Health, Imperial College London, 68 Wood Lane, London W12 7RH, UK; Diabetes Research Centre, University of Leicester, Gwendolen Road, Leicester LE5 4PW, UK; University of Galway, University Road, Galway H91 TK33, Ireland; Department of Medicine, University of Padua, Via Ospedale Civile 77, Padua 35121, Italy; IdiPaz Research Institute, Paseo de la Castellana 261, Madrid 28046, Spain; Department of Cardiology, The Midland Metropolitan University Hospital, Grove Lane, Birmingham B66 2QT, UK; Imperial Clinical Trials Unit, School of Public Health, Imperial College London, 68 Wood Lane, London W12 7RH, UK; Department of Primary Care and Public Health, School of Public Health, Imperial College London, 90 Wood Lane, London W12 0BZ, UK; Imperial Clinical Trials Unit, School of Public Health, Imperial College London, 68 Wood Lane, London W12 7RH, UK

**Keywords:** Lipid-lowering therapies, Acute coronary syndrome (ACS), Implementation, Digital tools, Randomized controlled trial, Precision medicine

## Abstract

**Aims:**

Lipid-lowering therapy (LLT) after acute coronary syndrome (ACS) typically follows stepwise intensification, delaying use of combination therapies and low-density lipoprotein cholesterol (LDL-C) goal attainment. We assessed whether access to a decision support system (DSS) altered the intensity of LLT prescribing vs. standard-of-care (SoC).

**Methods and results:**

Pragmatic, multinational, parallel 1:1 cluster-randomized controlled trial of ACS patients comparing mandatory access to a DSS (providing estimates of cardiovascular events and benefits from different LLT scenarios) to SoC. The primary endpoint was the proportion receiving intensified monotherapy or initiated/escalated combination LLT by Week 16 compared to pre-admission LLT; secondary endpoints included individual components of the primary endpoint, proportions at goal (LDL-C < 1.4 mmol/L), and timing of LLT escalations. 42 sites from UK, Italy, and Spain were randomized, enrolling 1139 participants, 79% male, median age 62 years (IQR: 55, 69), 84% without prior CVD, 69% LLT-naïve at admission, and median admission LDL-C 3.0 mmol/L (IQR: 2.46, 3.75). The primary endpoint was met in 71.7% (DSS) vs. 65.7% (SoC) and risk ratio (RR) 1.11 (95%CI:0.92–1.33, *P* = 0.29). Intensification of monotherapy occurred in 9.0% vs. 13.1% (RR: 0.68, 95%CI: 0.46–1.00), combination LLT in 61.6% vs. 50.6% (RR: 1.35, 95%CI: 0.93–1.98). LDL-C goal achievement was 54.8% vs. 50.3% (RR 1.06, 95%CI: 0.88–1.28), with LLT escalation before discharge in 64.8% vs. 60.7%.

**Conclusion:**

Access to a DSS, in hospitals managing ACS, did not improve LLT intensification within 16 weeks or LDL-C goal attainment but revealed a favourable trend towards earlier combination LLT use, which merits larger, longer studies in other settings.

## Graphical Abstract

**Graphical Abstract:**
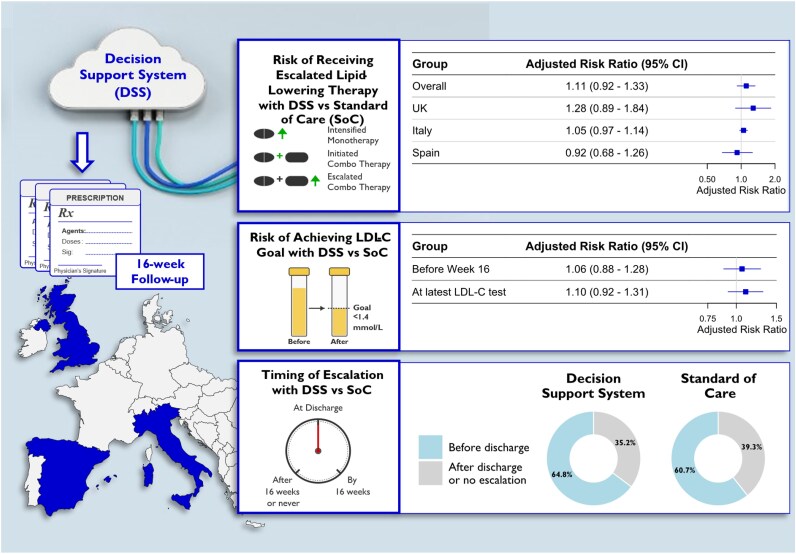
The ZODIAC trial evaluated whether a decision support system improves lipid-lowering therapy after acute coronary syndrome. While not significantly improving overall therapy intensification or low-density lipoprotein cholesterol goal attainment, trends toward earlier combination therapy use were observed, suggesting potential value in settings with suboptimal guideline implementation.

Key Findings

- Patients whose doctors had access to the decision support system were no more likely to receive intensified cholesterol-lowering therapy (71.7% vs. 65.7%) or achieve cholesterol levels below 1.4 mmol/L (54.8% vs. 50.3%) within 16 weeks compared to standard care.
- The DSS showed promise in encouraging doctors to use more potent combination cholesterol-lowering therapy earlier (61.6% vs. 50.6%), suggesting potential benefits that warrant investigation in larger, longer studies to determine whether the DSS improves prescribing and cholesterol lowering beyond 16 weeks.

## Introduction

Patients with a recent acute coronary syndrome (ACS) are at high risk of recurrent atherosclerotic cardiovascular disease (ASCVD) events.^1^ Despite robust evidence supporting intensive low-density lipoprotein cholesterol (LDL-C) lowering after ACS, with each 1.0 mmol/L reduction associated with ∼22% reduction in major vascular events,^2–5^ implementation gaps persist, with most patients failing to achieve recommended LDL-C goals.^6^ Current guidelines advocate stringent LDL-C control to lower LDL-C by at least 50% and below 1.4 mmol/L, achieved through a series of iterative steps, starting with statins and then adding additional therapies if LDL-C goals are not attained.^7^ However, this stepwise recommendation fails to recognize that 75% of patients are lipid-lowering therapy (LLT)-naïve when presenting with ACS^8^ and the current LDL-C goals of <1.4 mmol/L are frequently beyond the reach of statin monotherapy,^9,10^ meaning combination therapies will inevitably be needed for ∼80% of patients with ACS.^9^

However, as recent evidence from registries has shown, delaying the timing of achievement of optimal cholesterol control results in avoidable adverse cardiovascular outcomes.^11^ The current stepwise approach requires sequential LDL-C measurements to guide treatment escalation. Therefore, we sought to assess whether physicians’ behaviour could be modified using quantifiable measures of risk and potential benefit from different LLT regimens available to practising clinicians. Combining aspects of a baseline risk calculator using the SMART risk equation,^12^ and a lifetime benefit calculator that quantifies relative risk reduction from absolute changes in LDL-C,^13^ Imperial College London developed a decision support system (DSS). The DSS offers clinicians the opportunity for early individualized, LLT optimization by visualizing a risk and benefit quantifiable approach. Whether this approach might result in earlier escalation of LLT (increasing intensity of monotherapy or use of combination LLT) regimens and thus achieve the guideline-recommended LDL-C goals earlier was evaluated in the OptimiZation Of lipid lowering therapies using a DSS. In patients with Acute Coronary syndrome (ZODIAC) cluster-randomized controlled trial conducted across three European countries, which also assessed usability of the DSS.

## Methods

### Trial design

ZODIAC was a pragmatic, multinational, parallel cluster-randomized controlled trial conducted across 42 secondary, tertiary, and mixed-care sites in the UK, Spain, and Italy that enrolled participants between April 2023 and July 2024. The protocol available in Supplementary Document  *S1* was designed in adherence to the CONSORT statement for cluster-randomized clinical trials (see Supplementary Method  *S1*). This effectiveness trial evaluated the impact of access to the DSS on LLT escalation compared to the standard of care (SoC). As the DSS only provided visualization of risk and benefit from existing, licensed LLT options, with clinicians responsible for therapeutic decisions, safety outcomes were not required. However, device deficiencies were monitored as required for investigational software medical devices. The trial was registered on ClinicalTrials.gov (NCT05844566).

### Sites and participants

Sites were required to have the capability to use the DSS with willingness to undertake training in its use. LLT prescribing was unrestricted, provided it adhered to national guidelines and reimbursement policies.

Adults aged 18–79 years admitted with ACS were eligible. Key exclusion criteria included inability to provide written informed consent or baseline LDL-C < 1.8 mmol/L, as levels below this may have biased the patient against treatment intensification.^14^ Complete eligibility criteria are provided in Supplementary Method  *S2*.

### Randomization

Sites were randomized to either DSS availability or SoC, stratified by country and site type. Randomization lists were secured in a restricted system to maintain allocation concealment. Due to the intervention's nature, site and clinician blinding were not possible. Patient participants were blinded to their site’s group assignments.

### Ethics and regulatory approvals

ZODIAC was conducted according to the Declaration of Helsinki and Good Clinical Practice guidelines, with ethical approvals and informed consents obtained from all participants. The DSS was approved as an investigational medical device in the UK, Italy, and Spain.

### Intervention and standard of care

**Figure 1:**
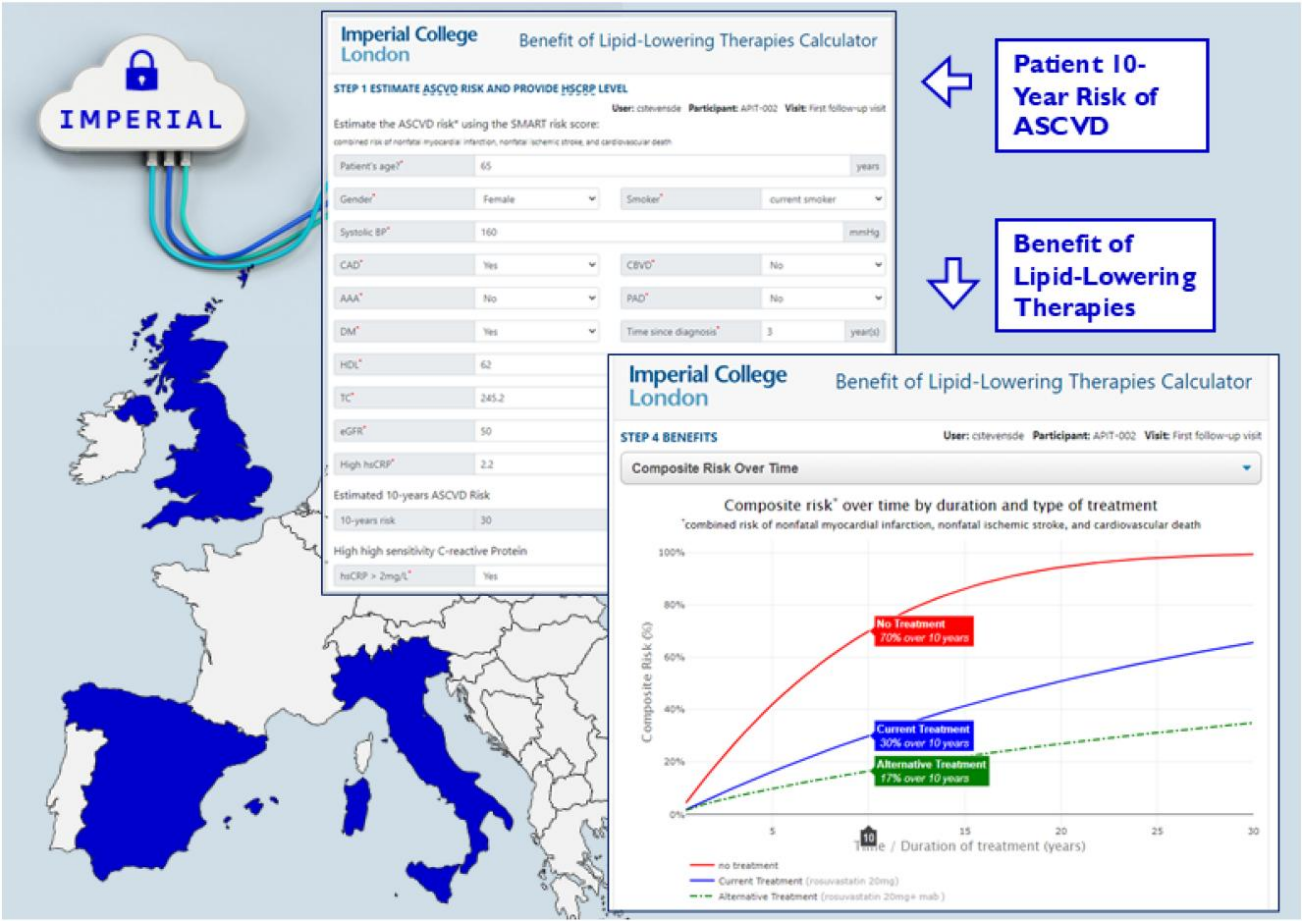
Decision support system screenshots showing (left) SMART risk calculator for 10-year ASCVD risk estimation and (right) visualization of risk trajectories under different lipid-lowering treatment regimens.

The DSS was a web application available (in English) online at https://zodiac.study. It provided clinicians with personalized, quantifiable visualization of ASCVD risk and the potential benefit of LLT optimization in terms of 30-year risk reduction (*Figure 1*, Supplementary Method  *S3*). The DSS combined the 10-year risk of recurrence of ASCVD using the SMART risk score^12^ and the projected benefits of various LLT regimens selected by the clinician (user) based on patient-specific profiles using a time-dependent benefit calculator^13^ (see Supplementary material online, *Figures S1* and *S2*). Prescribing clinicians in the DSS group received standardized training through two components: first, training conducted at the end of the site initiation visit video call, where the software engineer presented a recorded demonstration and answered questions from site staff; second, individual site users were required to watch an e-learning video and pass an online quiz to obtain certification required for DSS access. Clinicians were required to access the DSS prior to discharge following index ACS (access was optional during follow-up), while the control group followed standard care without DSS access (see Supplementary material online, *Figure S3*). Follow-up visits were conducted as per usual clinical care and were not mandated at specific intervals. Only data collected up to Week 16 post-ACS were included in the primary outcome analysis, as most patients were expected to have at least one visit in that period.

### Data collection

Data were collected using electronic case report forms and managed in the OpenClinica secure database, with regular monitoring to ensure quality.

### Endpoints

The primary endpoint was the composite proportion of patients who received intensified monotherapy, or initiated combination LLT, or escalated combination LLT within 16 weeks post-ACS. The expected percentage reduction in LDL-C for monotherapies is provided in Supplementary Method  *S4*.

Secondary endpoints included the individual components of the primary endpoint, the proportion of participants reaching LDL-C levels <1.4 mmol/L (<55 mg/dL) by Week 16, and the timing of LLT intensification (at discharge or during follow-up). Exploratory outcomes included the proportion of patients stratified by LLT potency (categories in Supplementary Method  *S5*) at discharge and the proportion achieving LDL-C goals at Week 16 based on the LLT regimen at discharge. The usability was evaluated using System Usability Scale questionnaires distributed after trial completion.

Follow-up LDL-C measurements beyond Week 16 were not mandatory and only used if performed and thus available. A sensitivity analysis of LDL-C goal attainment included lipid measurements available from 16 to 37 weeks post-ACS to assess consistency of findings beyond the primary 16-week timeframe.

### Sample size calculation

The original sample size was calculated assuming a 7% use of combination therapy or escalated monotherapy in the SoC group, with a 10% absolute increase in the DSS sites at the end of a 24-week follow-up (7% vs. 17%).^15,16^ Due to time constraints, the follow-up period was shortened to 16 weeks on 17 January 2024. The proportions were updated to 5% and 15%, respectively, based on prior studies,^14^ and accounting for a shorter follow-up.

To detect this, 10% difference with 90% power at the 5% significance level with a two-sided statistical test, assuming an intraclass correlation coefficient of 0.1^17^ and similar cluster sizes, the trial required 744 participants across 24 clusters per arm. Accounting for a 5% loss to follow-up, the target sample size was 1584 patients. Calculations were made using the ‘Tests for Two Proportions in a Cluster-Randomised Design’^18^ and PASS 2022 software.

### Statistical analysis

The primary endpoint was derived programmatically from LLT prescribed prior to admission, upon discharge, and during follow-up. A blinded clinician independently reviewed the primary endpoint for 10% randomly selected participants. This primary estimand used a ‘modified intention-to-treat’ basis in all eligible participants alive at Week 16, regardless of DSS use.

A generalized linear mixed model with a log link function and Poisson distribution was used to estimate the intervention effect on the primary endpoint, adjusting for country and site type while accounting for the clustering effect by site. Multilevel multiple imputation was performed for missing primary outcome data using the JOMO package in R statistical software version 4.4.2. Two sensitivity analyses were conducted: one using the full ITT population, including patients who died by Week 16 with missing data imputation and another using participants with complete outcome data only. Subgroup analyses used the primary analysis model with treatment-by-subgroup interaction terms. Binary secondary outcomes were analysed using the same approach as the primary outcome. All analyses used Stata/IC version 17 (StataCorp, College Station, TX, USA). The statistical analysis plan is provided in Supplementary Document  *S2*.

## Results

### Participating sites and study population

**Figure 2:**
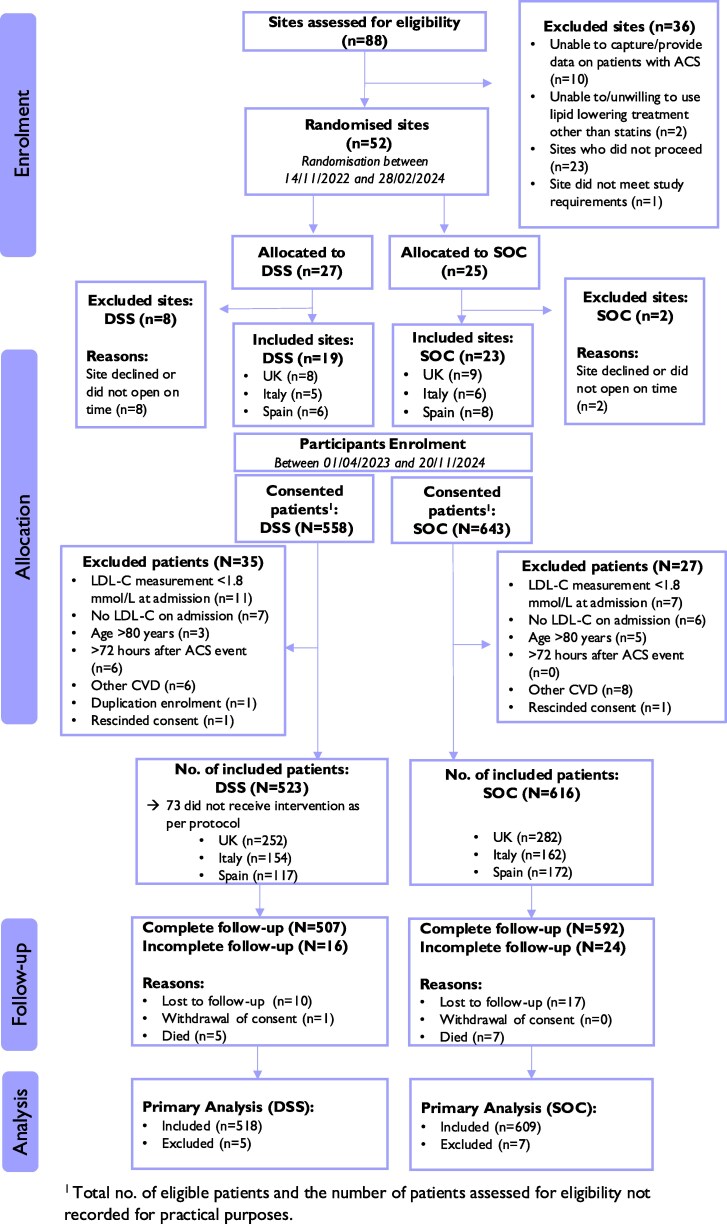
CONSORT diagram. DSS, decision support system; SoC, standard of care.

The trial enrolled 1139 participants across 42 sites in the UK, Italy, and Spain, with 616 participants from 23 sites in the SoC group and 523 from 19 sites in the DSS group (*Figure 2*). During the 16-week follow-up, 81.3% of participants had a single visit, with a median of 82 days after baseline (see Supplementary material online, *Table S1*), and 96.5% of participants (1099/1139) completed follow-up. The DSS was used per protocol before index ACS discharge for 86% (450/523) of participants in the DSS arm.

### Baseline characteristics

Participant baseline characteristics were similar between randomized groups (*Table 1*). Participants had a mean age of 62 years (IQR 55, 69), were predominantly male (78.9%), with prior coronary artery disease in 12.5% and prior ASCVD in 15.8%, 34.5% were current smokers, and 19.4% had diabetes. The median baseline LDL-C was 3.0 mmol/L (IQR 2.5, 3.8), with 68.8% of participants being LLT-naïve before the index ACS event.

**Table 1:** Patient baseline characteristics and medication before index ACS event

	Trial arms		
	Standard of care (*n* = 616)	Decision support system (*n* = 523)	Overall (*n* = 1139)
Age (years)^a^	61 (55,69)	62 (55,69)	62 (55,69)
Male sex^b^	502 (81.5)	397 (75.9)	899 (78.9)
Diabetes^b^	117 (19.0)	104 (19.9)	221 (19.4)
Smoking history^b^			
Current regular smoker (everyday)	221 (35.9)	172 (32.9)	393 (34.5)
Current occasional smoker	16 (2.6)	9 (1.7)	25 (2.2)
Former smoker	183 (29.7)	159 (30.4)	342 (30.0)
Never smoked	194 (31.5)	183 (35.0)	377 (33.1)
Systolic blood pressure (mmHg)^c^	128 (23)	126 (19)	127 (21)
Prior cardiovascular disease^b^	94 (15.3)	86 (16.4)	180 (15.8)
Prior coronary artery disease^b^	67 (10.9)	75 (14.3)	142 (12.5)
10-Year risk of CVD using SMART^a,d^	—	14.4 (10.9,22.8)	—
Years since first ASCVD diagnosis in CVD patients^a^	10 (3,14)	10 (4,14)	10 (4,14)
Total cholesterol (mmol/L)^a^	4.8 (4.2,5.7)	5.0 (4.2,5.8)	4.9 (4.2,5.7)
LDL-cholesterol (mmol/L)^a^	3.0 (2.5,3.7)	3.0 (2.4,3.8)	3.0 (2.5,3.8)
HDL-cholesterol (mmol/L)^a^	1.1 (0.9,1.3)	1.2 (1.0,1.4)	1.1 (1.0,1.3)
Triglycerides (mmol/L)^a^	1.4 (1.0,1.9)	1.4 (1.1,2.0)	1.4 (1.0,2.0)
LLT prior to admission^b^			
LLT-naïve	416 (67.5)	368 (70.4)	784 (68.8)
Statin monotherapy: low intensity	15 (2.4)	8 (1.5)	23 (2.0)
Statin monotherapy: medium intensity	133 (21.6)	105 (20.1)	238 (20.9)
Statin monotherapy: high intensity	24 (3.9)	23 (4.4)	47 (4.1)
Ezetimibe monotherapy	6 (1.0)	8 (1.5)	14 (1.2)
PCSK9-siRNA monotherapy	1 (0.2)	0 (0.0)	1 (0.1)
PCSK9 monoclonal antibodies	0 (0.0)	0 (0.0)	0 (0.0)
Combination: moderate intensity	4 (0.6)	1 (0.2)	5 (0.4)
Combination: high intensity	9 (1.5)	7 (1.3)	16 (1.4)
Combination: very high intensity	5 (0.8)	1 (0.2)	6 (0.5)
LLT not classified	3 (0.5)	2 (0.4)	5 (0.4)

CVD, cardiovascular disease; LLT, lipid-lowering treatment; PCSK9, proprotein convertase subtilisin/kexin type 9; siRNA, silencer RNA.

^a^Median IQR.

^b^ *n*, %.

^c^Mean (SD).

^d^The 10-year risk of ASCVD was calculated by the DSS, but only in the DSS arm where the system was implemented.

### Primary endpoint

**Figure 3:**
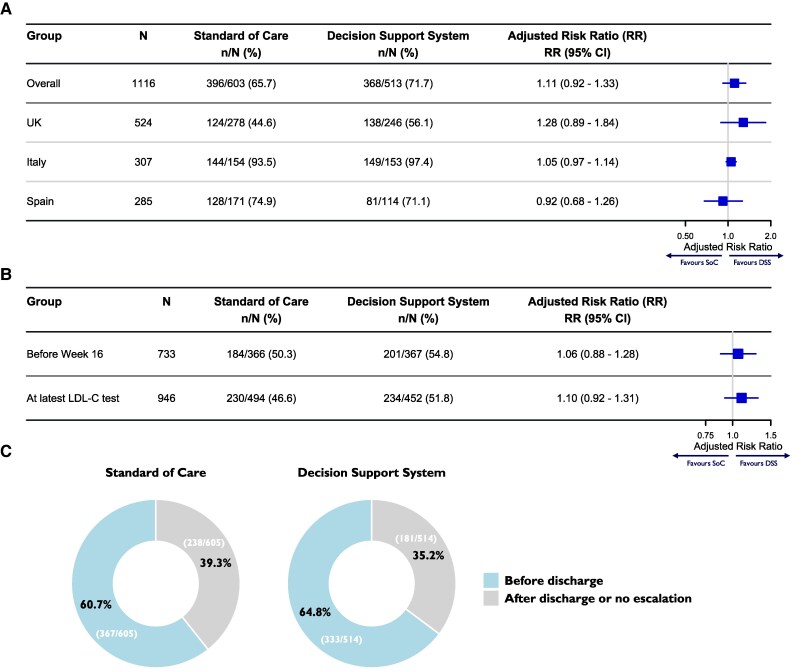
Risk ratios adjusted by country and site for: (*A*) intensification of monotherapy or initiation/escalation of combination lipid-lowering treatment by Week 16. (*B*) Low-density lipoprotein-cholesterol goal attainment (<1.4 mmol/L, < 55 mg/dL) by Week 16. (*C*) Timing of lipid-lowering therapy escalations. Abbreviations: LDL-C, low-density lipoprotein cholesterol.

Of the 1139 participants enrolled, 12 were excluded due to death before 16 weeks, leaving 1127 for the primary endpoint analysis (with data imputed for 11 participants). Intensification of monotherapy or initiation/escalation of combination therapy during the 16 weeks post-ACS event occurred in 71.7% of participants with complete outcome data (368/513) in the DSS arm and 65.7% (396/603) in SoC. After missing data imputation, the adjusted risk ratio (adjusted-RR) was 1.11 (95%CI 0.92,1.33), accounting for country, site, and site type (*Figure 3A*). In the UK, Italy, and Spain, the primary endpoint occurred in 56.1% (138/246), 97.4% (149/153), and 71.1% (81/114) in the DSS arm vs. 44.6% (124/278), 93.5% (144/154), and 74.9% (128/171) in SoC, respectively. Adjusted-RRs were 1.28 (95%CI 0.89,1.84), 1.05 (95%CI 0.97,1.14), and 0.93 (95%CI 0.68,1.26), respectively. Sensitivity analyses (see Supplementary material online, *Tables S2* and S3) and subgroup analyses by sex, age, history of diabetes and ASCVD, and LLT before ACS showed no significant effect of DSS access on LLT intensification (see Supplementary material online, *Figure S4*).

### Secondary endpoints

#### Type and timing of LLT escalation

Analysis of the individual components of the primary endpoint showed that initiation of combination therapy during the 16 weeks post-ACS event occurred in 61.6% participants (316/513) in the DSS arm and 50.6% (305/603) in SoC, with adjusted-RR 1.35 (95%CI 0.93,1.98). Intensification of monotherapy occurred in 9% of participants (46/513) in the DSS arm and 13.1% (79/603) in SoC, with adjusted-RR 0.68 (95%CI 0.46,1.0). Escalation of combination therapy occurred in 1.2% (6/513) in the DSS arm and 2% (12/603) in SoC, with adjusted-RR 0.63 (95%CI 0.22,1.79) (see Supplementary material online, *Figure S5*).

The primary endpoint occurred prior to discharge in 64.8% (367/605) of participants in the DSS arm and 60.7% (333/514) in SoC (*Figure 3C*). At discharge, 55.5% (290/523) in the DSS arm and 48.5% (299/616) in SoC received combination therapy.

#### LDL-C goal attainment

By Week 16, the LDL-C < 1.4 mmol/L goal was achieved in 54.8% (201/367) of participants in the DSS arm and 50.3% (184/366) in SoC (adjusted-RR 1.06, 95%CI: 0.88,1.28; *Figure 3B*). In an exploratory analysis including 213 LDL-C results obtained beyond 16 weeks, these figures were 51.8% (234/452) and 46.6% (230/494), respectively (adjusted-RR 1.10, 95%CI: 0.92,1.31). Lipid levels at 16 weeks are available in Supplementary material online, *Table S4*.

### Exploratory analyses

#### Potency of LLT and LDL-C goal attainment

**Figure 4:**
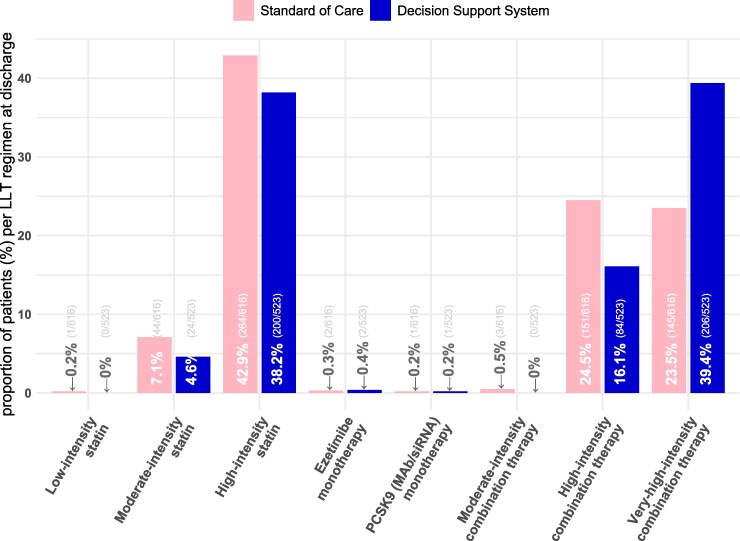
Distribution of lipid-lowering therapy at discharge. Abbreviations: PCSK9, proprotein convertase subtilisin/kexin type 9; MAb, monoclonal antibodies; siRNA, silencing RNA.

Among participants receiving combination therapy at discharge, 71% (206/290) in the DSS arm and 48.5% (145/299) in SoC received very-high-intensity combination therapy (≥65% LDL-C reduction)—*Figure 4*. Across both groups, among participants receiving very-high-intensity combination therapy at discharge, 85% (297/351) received dual therapy, and 15% (54/351) received triple therapy. An injectable LLT was included for 1% (3/297) and 81% (44/54) of the participants receiving dual and triple therapy, respectively. Overall, among patients prescribed a high-intensity combination therapy regimen (50% to <65% LDL-C reduction) at discharge or follow-up, most received ezetimibe in addition to either atorvastatin 40 mg (198/289; 69%) or rosuvastatin 20 mg (60/289; 21%). Among those prescribed very-high-intensity therapy (≥65% LDL-C reduction), most (323/443, 73%) received atorvastatin 80 mg with ezetimibe. While patients were frequently prescribed high or very-high intensity combination therapies, these were less commonly prescribed than atorvastatin 80 mg monotherapy (465/1139; 40.82%).

**Figure 5:**
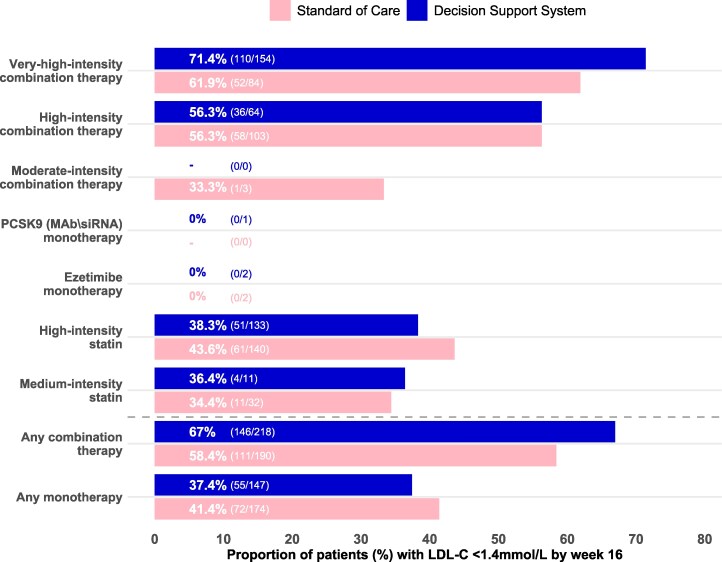
Low-density lipoprotein-cholesterol goal attainment (<1.4 mmol/L, < 55 mg/dL) stratified by lipid-lowering therapy at discharge. Abbreviations: PCSK9, proprotein convertase subtilisin/kexin type 9; MAb, monoclonal antibodies; siRNA, silencing RNA; LDL-C, low-density lipoprotein cholesterol.

Of participants receiving monotherapy post-ACS discharge with available LDL-C by Week 16, 37.4% (55/147) in the DSS arm and 41.4% (72/174) in SoC achieved LDL-C goal. For combination therapy, 67% (146/218) in the DSS arm and 58.4% (111/190) in SoC achieved LDL-C goals. In participants receiving very-high-intensity combination LLT, 71.4% (110/154) in the DSS arm and 61.9% (52/84) in SoC achieved the LDL-C goal (*Figure 5*).

#### Usability of the DSS

The DSS Usability Evaluation Questionnaire was fully completed by at least one user per DSS site, which represented 46.7% of all users across DSS sites. The median overall usability score was 70 out of 100 (IQR 65–75; Supplementary material online, *Table S5*), with 90.5% feeling confident using the tool. No device deficiencies were identified during the trial.

## Discussion

In this cluster-randomised clinical trial, access to a DSS did not significantly improve the intensification of lipid-lowering monotherapy or initiation/escalation of combination therapy for patients admitted to hospital with an ACS compared with SoC. Similarly, there was no significant difference in LDL-C goal achievement between groups, with approximately half of the patients in both arms achieving the LDL-C goal of <1.4 mmol/L (55 mg/dL) by Week 16. However, a favourable trend toward greater use of combination therapy and in particular very high-intensity regimens was observed in the DSS arm, potentially indicating an impact on prescribing patterns despite the neutral outcome.

Beyond these clinical results, this trial provided important insights into the feasibility, usability, and contextual efficacy of this digital intervention.^19^ Early-phase evaluations typically focus on establishing whether a digital system works as intended in a given context, whether it can be used effectively by the target users, and whether it shows promise of efficacy in controlled settings. While measures of efficacy were neutral, we successfully demonstrated stable functionality of the digital tool (no device deficiencies or prolonged ‘downtime’), and when user acceptability was assessed using a qualitative survey, all DSS sites responded with the DSS being well-received, high usability ratings, no safety concerns, and reports of easy integration into practice. The high usability scores suggest that technical barriers were minimal and unlikely to impair clinical decision-making.

The neutral primary outcome should be considered in the context of unexpected practice patterns observed in our study. Previous studies indicate that LLT initiation post-ACS typically begins with high-intensity statins before hospital discharge, with stepwise escalation typically with the addition of ezetimibe and injectable PCSK9-directed therapies. Given the iterative nature of this process, this results in delays in treatment escalation and poor attainment of lipid goals.^11^ Reports suggest that use of combination therapy is low post-ACS, ranging between 17 and 22% in routine clinical practice at 24 weeks post-ACS.^15,16^ In the present study, a high proportion of patients in the SoC group received combination LLT at discharge, contrasting with real-world data. The higher-than-expected use of combination therapy may reflect the Hawthorne effect or possibly an evolving shift in clinical practice, where LLT intensification occurs earlier in response to accumulating evidence about failures to implement guidelines.^6,20^ It is also likely influenced by the inclusion of centres with a specific interest in LLT research, which may be inherently more proactive in applying intensive lipid-lowering strategies. The awareness of being observed in a clinical trial leads to changes in prescribing behaviour.^21^ Increased attention to and close monitoring of guideline-based therapy during the trial may have prompted more intensive LLT across both arms.

This high use of combination therapy as part of the routine care pathway in both arms may have limited our ability to detect a significant difference with the DSS based on the initial sample size, as most patients were already discharged on combination therapy, inherently restricting room for further intensification. Importantly, this limitation highlights the context-dependent nature of the DSS's efficacy. The DSS is likely to have greater utility in contexts where treatment intensification follows a stepwise approach, such as when patients start with monotherapy and require up-titration, as evidenced by regional differences observed in our trial and other European studies. In the SANTORINI study, which assessed LLT in ASCVD patients across Europe, combination therapy rates were significantly higher in Spain and Italy (33–40%) compared to the UK, where only 4% of patients received combination therapy.^16^ Our study showed a trend towards more potent LLT prescribing with the DSS in the UK compared to Spain and Italy, though statistically insignificant. This geographical differences in the impact of the DSS underscore the critical role of context in digital health intervention efficacy and effectiveness and suggest that tools like the one we assessed may be particularly valuable in settings where guideline implementation lags behind, such as primary care, rather than in secondary care settings who already appear to be moving away from the stepwise LLT approach outlined in the 2019 ESC guidelines.

This unanticipated evolution in standard care practices highlights a critical consideration for digital health intervention evaluations: the context in which a system is deployed may change during the evaluation period, potentially diminishing the measurable impact of the intervention. The WHO framework for evaluating digital health interventions recognizes this challenge, emphasizing that early phase studies must be followed by adaptive evaluations that account for evolving practice patterns. Future evaluation of this DSS should consider targeting settings where the combination of LLT use remains suboptimal and incorporate adaptive trial designs that can account for rapidly evolving standards of care, thereby maximizing the potential to demonstrate the DSS efficacy in improving lipid management after ACS.

The trial’s neutral outcome might also be attributed to the fact that, while the mandatory use of the DSS provided estimations of the benefits of user-selected treatment regimen, the implementation of the output was discretionary. Even when the DSS provided evidence-based suggestions for treatment up-titration, clinicians were not obligated to act on them. This may have introduced variability in clinical decision-making, where some clinicians may have opted not to intensify therapy despite graphical demonstration of the benefits. Such decisions could stem from a combination of competing clinical priorities, or scepticism about the incremental benefits of further treatment adjustments. This highlights a critical challenge: addressing deeper behavioural barriers among clinicians.^22^ These barriers include misperceptions of cardiovascular risk, the efficacy of treatment intensification, and a tendency for clinicians to rely on perceived improvements in LDL-C reduction rather than on achievement of guideline-based thresholds. Addressing these factors may require more structured pathways in which the DSS is used, for example, in primary care settings, along with tailored education to reinforce the importance of achieving very low LDL-C levels and improving clinician confidence in prescribing combination therapies.

Lastly, a trend was observed in our study towards greater use of very-high-intensity LLT in the DSS group, both early in the treatment course and overall. This included regimens capable of achieving ≥65% LDL-C reduction, such as high-intensity statins combined with ezetimibe and bempedoic acid (triple combination) or the addition of PCSK9 inhibitors, which are the most potent class apart from statins. As prescribing patterns favoured more potent LLT strategies, there was a corresponding decline in the use of lower-intensity therapies, which may still achieve LDL-C reductions of ≥50%, often through moderate- or high-intensity statins combined with ezetimibe or high-intensity statins alone. Given the mutually exclusive nature of these regimens, this redistribution suggests a more proactive approach to lipid management in the DSS arm. Since the primary endpoint encompassed both high- and very-high-intensity regimens, this redistribution may have attenuated the estimates of overall efficacy of the DSS. From a clinical perspective, the increased use of very-high-intensity regimens in the DSS arm aligns with evidence suggesting that these strategies are more effective in achieving LDL-C targets.^9^ Moreover, we only assessed 16 weeks post-ACS, and continued therapy escalation beyond discharge would have been required to fully assess the potential clinical utility of the DSS in care pathways. Future research should explore whether structured DSS integration into clinical workflows can further facilitate timely and appropriate intensification of therapy, particularly in settings where combination treatment remains underutilized, with future implementations prioritising electronic medical record integration, automated data population, and embedded clinical decision support—potentially incorporating AI-driven treatment suggestions that clinicians can approve or adapt to individual patient circumstances.

Even with the observed trend toward more combination therapy use in the SoC group, additional treatment measures would still be necessary to bring all patients to their LDL-C goals. Simulation studies based on DA VINCI data suggest that in patients with ASCVD, ∼90% LDL-C goal attainment (<1.4 mmol/L) would require a combination of statins and ezetimibe in about one-third of patients, while the remaining two-thirds would need a PCSK9 inhibitor,^9^ or another oral agent.^23^ This highlights the need for continued therapy escalation beyond discharge, even among patients already on combination therapy. In this context, decision-support tools like the DSS may be useful in facilitating evidence-based treatment intensification, particularly as many clinicians are unaware of the benefits of early intensification of LLT and its potential legacy benefits for future cardiovascular events.^24^

### Strengths and limitations of the trial

The ZODIAC trial has several notable strengths. It was pragmatic, ensuring practical applicability, allowing decisions on treatment intensification to be made flexibly and in alignment with real-world practices. It tested a personalized approach to LLT optimization, providing risk-informed treatment selection based on individual patient profiles. The trial implementation strategy focused on the critical window before hospital discharge, when patient motivation for secondary prevention is high. Early escalation of LLT during this period is particularly impactful, as delays in treatment optimization often result in prolonged periods of suboptimal LDL-C control and increased cardiovascular risk.^11^ The multinational design across diverse healthcare systems across the UK, Italy, and Spain captured variations in clinical practice and LLT accessibility. The availability of intensive LLT options across participating countries ensured that the potential of the DSS could be assessed vs. SoC in a setting where all guideline-recommended treatments were accessible. The DSS incorporated several key components from established frameworks,^25^ including individualized risk–benefit estimation, educational outreach features, and audit-and-feedback mechanisms—approaches consistently shown to enhance prescribing behaviours—all integrated into a user-friendly interface that generated actionable treatment recommendations.

Despite its strengths, the ZODIAC trial also had several limitations. A notable limitation was the failure to achieve the planned sample size, with 1139 of the intended 1584 cases (∼70%) included in the final analysis. In addition, the initial event rate assumptions—occurrence of LLT intensification in the SoC arm—were significantly underestimated, resulting in a smaller-than-expected effect size. A larger trial with more sites and revised assumptions about event rates may have been better positioned to reveal statistically significant differences. The lack of a pilot study limited the ability to pre-emptively adjust for changes in care pathways or clinician behaviour, which may have influenced the observed outcomes. Furthermore, differences in healthcare systems across the UK, Italy, and Spain, and the exclusive focus on Western European countries, may limit the generalizability of the findings to other global regions, especially given the predominance of UK sites. The trial’s short follow-up period of 16 weeks may not have been sufficient to observe long-term trends in LDL-C. Longer follow-up durations would provide greater clarity on the sustained impact of DSS-guided treatment intensification at multiple visits, each offering an opportunity for intensification based on residual risk (DSS) vs. an LDL-C (at goal/not at goal) approach. Another key limitation was the voluntary use of the DSS during follow-up visits; while use at baseline was mandatory, there was no requirement for clinicians to act on the DSS outputs. This highlights a critical challenge—the presence of a DSS does not guarantee changes in prescribing behaviour until barriers, such as risk misperception, are addressed. Additionally, we did not systematically assess inter-clinician variability in interpreting DSS outputs and subsequent treatment decisions. However, evaluation of users via the System Usability Scale showed that 90.5% of users felt confident using the tool, suggesting that interpretation of the DSS was generally not problematic for most clinicians. While our DSS was intentionally designed to preserve clinical autonomy and some variability in decision-making is expected and appropriate, future studies could evaluate whether standardized case scenarios yield consistent clinical responses across different users. Availability and timing of lipid measurements during follow-up were variable, which may have influenced the assessment of goal attainment. Lastly, we did not systematically capture adverse events, statin intolerance, or adherence to medications. The overall number of individuals not on any statin was 1.67% at discharge and 2.63% at 16 weeks. Moreover, statin intolerance is only likely to be relevant for the one-third of patients admitted on LLT, as the majority were LLT naïve at index ACS. In the latter patients, statin intolerance is likely to emerge during the follow-up period, which may influence subsequent treatment intensification decisions. Future studies of longer duration tracking adverse events and adherence could provide valuable context for understanding the real-world impact and safety of DSS-guided treatment intensification.

### Conclusion

The ZODIAC trial provides critical insights into personalized risk and benefit-based DSS tools for optimizing LLT in ACS patients. While the primary outcome was neutral, the findings might suggest that DSS tools may support clinicians in prescribing more potent combination therapies and reveal important contextual factors that influence digital intervention efficacy, such as variability in guideline implementation at the country level. Furthermore, DSS tools may offer information but may not mandate behaviour change; hence, understanding how to address clinician behavioural barriers should be part of digital intervention development. Finally, insights from ZODIAC provide a foundation for future research that should aim to evaluate the DSS in primary care settings, where most patients are treated, guideline implementation lags behind most, and the Electronic Health Record systems often at regional/national level, may lend themselves to scalability.

## Supplementary Material

ztaf135_Supplementary_Data

## Data Availability

The data described in this manuscript will not be made publicly available. However, anonymized data collected will be made available upon request for audit and quality control purposes only. This approach ensures compliance with ethical standards and protects the privacy of participants.

## Author information

ZODIAC Trial: United Kingdom—Imperial College London: Kausik K Ray, Victoria Cornelius, Neil R Poulter, Gaia Kiru, Leila Janani, Jessica Smith, Christophe AT Stevens, Maria Moreno Morales, Annie Wright, Eloise Britten, Felicia Frost, Francesco Lala, Jonathan Dao, Karen Danois, Lisa Feng, Lorna Hazell, Nandita Kaza, Nayan Das, Safa Anwar, Sima Toopchiani, Smita Das, Stephanie Amiridis, Vanaja Kakarla, Yasmin Abdat; The Midland Metropolitan University Hospital: Derek Connolly; University of Leicester: Kamlesh Khunti; Birmingham City Hospital, Sandwell & West Birmingham NHS Trust: Vinoda Sharma; Conquest Hospital, East Sussex Healthcare NHS Trust: Christina Elorz; Craigavon Area Hospital, HSC Southern Health and Social Care Trust: Andrew Moriarty; Freeman Hospital, Newcastle upon Tyne Hospitals NHS Foundation Trust: Afzar Zaman; Glan Glwyd, Betsi Cadwala University Health Board: Satheesh Balakrishnan-Nair; Hammersmith Hospital, Imperial College Healthcare NHS Trust: Rasha Al-Lamee; Huddersfield Royal Infirmary, Calderdale and Huddersfield NHS Foundation Trust: Azeem Sheikh; Kettering General Hospital, Kettering General Hospital NHS Foundation Trust: Mohamed Alama; Luton and Dunstable University Hospital, Bedfordshire Hospitals NHS Foundation Trust: George Hunter; North Tyneside General Hospital, Northumbria NHS Foundation Trust: David Ripley; Royal Bournemouth Hospital, University Hospitals Dorset NHS Foundation Trust: Vivek Kodoth; Royal United Hospital, Royal United Hospitals Bath NHS Foundation Trust: Daniel McKenzie; Russell’s Hall Hospital, Dudley Group NHS Foundation Trust: Joe Martins; Scunthorpe General Hospital, North Lincolnshire and Goole NHS Foundation Trust: Rashed Hossain; Sunderland Royal Hospital, South Tyneside and Sunderland NHS Foundation Trust: Sharad Agrawal; Worcestershire Royal Hospital, Worcestershire Acute Hospitals NHS Trust: Helen Routledge; Worthing Hospital, University Hospitals Sussex NHS Foundation Trust: Sukhbir Dhamrait. Italy—University Hospital of Padova: Alberto Zambon, A.O.U. Ospedali Riuniti U.O.C. Cardiologia e UTIC: Natale Brunetti; Arcispedale Santa Maria Nuova—Azienda USL/IRCCS Reggio Emilia: Alessandro Navazio; AUSL di Bologna-Ospedale Maggiore: Gianni Casella; Azienda Ospedaliera di Rilievo Nazionale ‘Sant’Anna e San Sebastiano’ di Caserta: Paolo Calabrò; Azienda Ospedaliera Universitaria Policlinico Gaetano Martino U.O. Cardiologia: Giuseppe Andò; Azienda Ospedaliero Universitaria: Claudio Fresco; IRCCS Ospedale Policlinico San Martino: Roberta Della Bona; IRCCS Policlinico San Donato: Andrea Borin; Ospedale di Cisanello—A.U.O.P. Azienda Ospedaliera Universitaria: Raffaele De Caterina; Ospedale Sandro Pertini—ASL Roma 2: Alessandro Sciahbasi; Policlinico di Modena S.C. di Cardiologia: Giuseppe Boriani. Spain—IdiPaz Research Institute: José L López-Sendón, Hospital A Coruña: Jesús Peteiro; Hospital Clínico Universitario Santiago de Compostela: Jose Ramón González Juanatey; Hospital Fundación Jiménez Díaz: José Tuñón; Hospital Gregorio Marañón: Manuel Martínez Sellés; Hospital HM Montepríncipe: Leire Unzué; Hospital Puerta de Hierro: Carlos Arellano; Hospital Reina Sofía: Jose Lopez Aguilera; Hospital Sant Pau: Alessandro Sionis; Hospital Universitario La Luz Quiron: Roberto Martín; Hospital Universitario La Paz: Raul Moreno; Hospital Universitario Rey Juan Carlos: Alejandro Villanueva; Hospital Vall d'Hebron: Jordi Lozano; Hospital Virgen Arrixaca: Domingo Pascual; Hospital Virgen Macarena: Francisco Javier Cortés. Ireland—University of Galway: John William McEvoy. Germany—RWTH University Hospital Aachen: Julia Brandts. Greece—University of Ioannina: Fotios Barkas.

## References

[1] Bergmark BA, Mathenge N, Merlini PA, Lawrence-Wright MB, Giugliano RP. Acute coronary syndromes. Lancet 2022;399:1347–1358. 35367005 10.1016/S0140-6736(21)02391-6 PMC8970581

[2] Cannon CP, Braunwald E, McCabe CH, Rader DJ, Rouleau JL, Belder R, et al Intensive versus moderate lipid lowering with statins after acute coronary syndromes. N Engl J Med 2004;350:1495–1504. 15007110 10.1056/NEJMoa040583

[3] Cannon CP, Blazing MA, Giugliano RP, McCagg A, White JA, Theroux P, et al Ezetimibe added to statin therapy after acute coronary syndromes. N Engl J Med 2015;372:2387–2397. 26039521 10.1056/NEJMoa1410489

[4] Schwartz GG, Steg PG, Szarek M, Bhatt DL, Bittner VA, Diaz R, et al Alirocumab and cardiovascular outcomes after acute coronary syndrome. N Engl J Med 2018;379:2097–2107. 30403574 10.1056/NEJMoa1801174

[5] Cholesterol Treatment Trialists C . Efficacy and safety of more intensive lowering of LDL cholesterol: a meta-analysis of data from 170,000 participants in 26 randomised trials. The Lancet 2010;376:1670–1681. 10.1016/S0140-6736(10)61350-5 PMC2988224 21067804

[6] Allahyari A, Jernberg T, Hagström E, Leosdottir M, Lundman P, Ueda P. Application of the 2019 ESC/EAS dyslipidaemia guidelines to nationwide data of patients with a recent myocardial infarction: a simulation study. Eur Heart J 2020;41:3900–3909. 32072178 10.1093/eurheartj/ehaa034 PMC7654933

[7] Mach F, Baigent C, Catapano AL, Koskinas KC, Casula M, Badimon L, et al 2019 ESC/EAS guidelines for the management of dyslipidaemias: lipid modification to reduce cardiovascular risk: the task force for the management of dyslipidaemias of the European Society of Cardiology (ESC) and European Atherosclerosis Society (EAS). Eur Heart J 2020;41:111–188. 31504418 10.1093/eurheartj/ehz455

[8] Schubert J, Lindahl B, Melhus H, Renlund H, Leosdottir M, Yari A, et al Low-density lipoprotein cholesterol reduction and statin intensity in myocardial infarction patients and major adverse outcomes: a Swedish nationwide cohort study. Eur Heart J 2020;42:243–252. 10.1093/eurheartj/ehaa1011 PMC7954251 33367526

[9] Brandts J, Bray S, Villa G, Catapano AL, Poulter NR, Vallejo-Vaz AJ, et al Optimal implementation of the 2019 ESC/EAS dyslipidaemia guidelines in patients with and without atherosclerotic cardiovascular disease across Europe: a simulation based on the DA VINCI study. Lancet Reg Health Eur 2023;31:100665. 37547279 10.1016/j.lanepe.2023.100665 PMC10398584

[10] Noack F, Schwaab B, Voeller H, Eckrich K, Guha M, Bongarth C, et al The current LDL-C target <1.4mmol/l of the ESC is achieved in less than 16% of patients with coronary heart disease despite effective lipid-lowering therapy: data from the LLT-R registry. Eur Heart J 2020;41:ehaa946-2998.

[11] Schubert J, Leosdottir M, Lindahl B, Westerbergh J, Melhus H, Modica A, et al Intensive early and sustained lowering of non–high-density lipoprotein cholesterol after myocardial infarction and prognosis: the SWEDEHEART registry. Eur Heart J 2024;45:4204–4215. 39217499 10.1093/eurheartj/ehae576 PMC11472424

[12] Dorresteijn JA, Visseren FL, Wassink AM, Gondrie MJ, Steyerberg EW, Ridker PM, et al Development and validation of a prediction rule for recurrent vascular events based on a cohort study of patients with arterial disease: the SMART risk score. Heart 2013;99:866–872. 23574971 10.1136/heartjnl-2013-303640

[13] Khan I, Peterson ED, Cannon CP, Sedita LE, Edelberg JM, Ray KK. Time-dependent cardiovascular treatment benefit model for lipid-lowering therapies. J Am Heart Assoc 2020;9:e016506. 32720582 10.1161/JAHA.120.016506 PMC7792260

[14] Koskinas KC, Windecker S, Pedrazzini G, Mueller C, Cook S, Matter CM, et al Evolocumab for early reduction of LDL cholesterol levels in patients with acute coronary syndromes (EVOPACS). JACC 2019;74:2452–2462. 31479722 10.1016/j.jacc.2019.08.010

[15] Koskinas KC, Gencer B, Nanchen D, Branca M, Carballo D, Klingenberg R, et al Eligibility for PCSK9 inhibitors based on the 2019 ESC/EAS and 2018 ACC/AHA guidelines. Eur J Prev Cardiol 2021;0:2047487320940102. 10.1177/2047487320940102 33755142

[16] Ray KK, Aguiar C, Arca M, Connolly DL, Eriksson M, Ferrières J, et al Use of combination therapy is associated with improved LDL cholesterol management: 1-year follow-up results from the European observational SANTORINI study. Eur J Prev Cardiol 2024;31:1792–1803. 38861400 10.1093/eurjpc/zwae199

[17] Ray KK, Molemans B, Schoonen WM, Giovas P, Bray S, Kiru G, et al EU-wide cross-sectional observational study of lipid-modifying therapy use in secondary and primary care: the DA VINCI study. Eur J Prev Cardiol 2021;28:1279–1289. 33580789 10.1093/eurjpc/zwaa047

[18] Campbell M, Grimshaw J, Steen N. Sample size calculations for cluster randomised trials. Changing professional practice in Europe group (EU BIOMED II concerted action). J Health Serv Res Policy 2000;5:12–16. 10787581 10.1177/135581960000500105

[19] World Health Organization . Monitoring and Evaluating Digital Health Interventions: A Practical Guide to Conducting Research and Assessment. Geneva, Switzerland: World Health Organization; 2016.

[20] Catapano AL, De Caterina R, Jukema JW, Klempfner R, Landmesser U, Schiele F, et al Addressing current challenges in optimization of lipid management following an ACS event: outcomes of the ACS EuroPath III initiative. Clin Cardiol 2023;46:407–415. 36799113 10.1002/clc.23988 PMC10106658

[21] Sedgwick P, Greenwood N. Understanding the Hawthorne effect. BMJ 2015;351:h4672. 26341898 10.1136/bmj.h4672

[22] Wang T, Tan J-Y, Liu X-L, Zhao I. Barriers and enablers to implementing clinical practice guidelines in primary care: an overview of systematic reviews. BMJ Open 2023;13:e062158. 10.1136/bmjopen-2022-062158 PMC9827241 36609329

[23] Ray KK, Catapano AL, Diamand F, Wolowacz S, Haq I, Bilitou A, et al Simulation of bempedoic acid in the lipid-lowering treatment pathway using the European contemporary SANTORINI cohort of high- and very high-risk patients. Eur Heart J 2022;43:ehac544-2377.

[24] Khunti K, Kosiborod M, Ray KK. Legacy benefits of blood glucose, blood pressure and lipid control in individuals with diabetes and cardiovascular disease: time to overcome multifactorial therapeutic inertia? Diabetes Obe Metab 2018;20:1337–1341. 10.1111/dom.13243 29405543

[25] Kawamoto K, Houlihan CA, Balas EA, Lobach DF. Improving clinical practice using clinical decision support systems: a systematic review of trials to identify features critical to success. BMJ 2005;330:765. 15767266 10.1136/bmj.38398.500764.8F PMC555881

