# Correction to: Optimizing lipid-lowering therapy for acute coronary syndrome using a decision support system: insights from a cluster randomized trial

**DOI:** 10.1093/ehjdh/ztag071

**Published:** 2026-05-19

This is a correction to: Christophe A T Stevens, Jessica Smith, Julia Brandts, Fotios Barkas, Maria Moreno Morales, Leila Janani, Gaia Kiru, Nandita Kaza, Victoria Cornelius, Neil R Poulter, Kamlesh Khunti, John William McEvoy, Alberto Zambon, Jose L Lopez-sendon, Derek Connolly, Lorna Hazell, Kausik K Ray, the ZODIAC trial, on behalf of, Optimizing lipid-lowering therapy for acute coronary syndrome using a decision support system: insights from a cluster randomized trial, *European Heart Journal - Digital Health*, Volume 7, Issue 2, March 2026, ztaf135, https://doi.org/10.1093/ehjdh/ztaf135

The following change has been made to the originally published paper. A corrected supplementary file has been supplied to the paper, in which the name Dr Imtiaz Kalyar has been added to those for the group of the Zodiac trial (from which list the name was omitted in error in the original publication).

