# Supplementary material for: Optimizing lipid-lowering therapy for acute coronary syndrome using a decision support system: insights from a cluster randomized trial
Source: Eur Heart J Digit Health. 2025 Nov 17;7(2):ztaf135. doi: 10.1093/ehjdh/ztaf135 (PMC12853117; doi:10.1093/ehjdh/ztaf135)

Supplementary Materials

**for**

**Optimising Lipid-Lowering Therapy for Acute Coronary Syndrome using a Decision Support System: Results from a Cluster Randomised Trial**

Christophe AT. Stevens, Jessica Smith, Julia Brandts, Fotios Barkas, Maria Moreno Morales, Leila Janani, Gaia Kiru, Nandita Kaza, Victoria Cornelius, Neil R. Poulter, Kamlesh Khunti, John William McEvoy, Alberto Zambon, Jose L. Lopez-Sendon, Derek Connolly, Lorna Hazell, Kausik K. Ray. on behalf of the ZODIAC trial

**Table of Contents**

[List of Collaborators 3](#_Toc193703235)

[Supplementary Methods 6](#_Toc193703236)

[Supplementary Method 1: CONSORT 2010 checklist of information to include when reporting a cluster randomised trial 6](#_Toc193703237)

[Supplementary Method 2: Full List of Inclusion and Exclusion Criteria 9](#_Toc193703238)

[Supplementary Method 3: Description of the Decision Support System 10](#_Toc193703239)

[Supplementary Method 4: Expected percentage low-density lipoprotein cholesterol (LDL-C) reduction used by the DSS and in the calculation of the primary endpoint. 12](#_Toc193703240)

[Supplementary Method 5: Description of the Lipid-Lowering Therapy Potency Categories 13](#_Toc193703241)

[Supplementary Tables 14](#_Toc193703242)

[Supplementary Table 1: Visit attendance pattern 14](#_Toc193703243)

[Supplementary Table 2: Sensitivity analysis of the effect of the DSS on proportion of participants initiated on LLT combination therapy, escalation of LLT monotherapy or escalation of LLT combination therapy in participants *with complete outcome data* 15](#_Toc193703244)

[Supplementary Table 3: Sensitivity analysis of the effect of the DSS on proportion of participants initiated on LLT combination therapy, escalation of LLT monotherapy or escalation of LLT combination therapy *inclusive of participants who died (full ITT)* 16](#_Toc193703245)

[Supplementary Table 4: Lipid levels at 16 weeks post-ACS. 17](#_Toc193703246)

[Supplementary Table 5: Responses to the System Usability Scale (SUS) Questionnaire (n=21) 18](#_Toc193703247)

[Supplementary Figures 19](#_Toc193703248)

[Supplementary Figure 1: Screenshot of the estimation of 10-year risk of ASCVD using the SMART calculator in the Decision Support System. 19](#_Toc193703249)

[Supplementary Figure 2: Screenshot of the risk trajectories under different Lipid Lowering Treatment (LLT) regimens over the next 30 years, which can help clinicians understand the benefits of changing different LLTs for a given patient using the Decision Support System. 20](#_Toc193703250)

[Supplementary Figure 3: Study design schema. 21](#_Toc193703251)

[Supplementary Figure 4: Subgroups analyses of the primary endpoint. 22](#_Toc193703252)

[Supplementary Figure 5: Effect of DSS on separate components of the primary endpoint; adjusted for site, site type and country. 23](#_Toc193703253)

[Supplementary Documents 24](#_Toc193703254)

[Supplementary Document 1: Protocol 24](#_Toc193703255)

[Supplementary Document 2: Statistical Analysis Plan (SAP) 70](#_Toc193703256)

# List of Collaborators

**Scientific Steering Committee:**

*Executive Committee:*

Prof. Neil Poulter, Prof. Kausik K Ray, Prof. Derek Connolly, Prof. José López-Sendón, Prof. Alberto Zambon, Prof. Kamlesh Khunti, Prof. John William McEvoy, Prof. Victoria Cornelius, Dr Leila Janani, Dr Gaia Kiru, Jessica Smith, Christophe Stevens, Maria Moreno Morales

*National Lead Investigators:*

Prof. Derek Connolly, University of Birmingham (UK National Lead Investigator), Prof. José López-Sendón, University Hospital La Paz (Spain National Lead Investigator), Prof. Alberto Zambon, University Hospital of Padova (Italy National Lead Investigator)

**Academic Research Organisations (AROs/CRO):**

*UK Trial Management/Central Data Management/Sponsor Organisation:*

Imperial Clinical Trials Unit (ICTU) - Global – Lead ARO/Sponsor

ICTU Global - Clinical Operations: Senior Operations: Gaia Kiru, Jessica Smith; Clinical Trial Managers: Karen Danois, Maria Moreno Morales; Clinical Trial Monitors: Sima Toopchiani, Stephanie Amiridis, Lisa Feng, Yasmin Abdat; Clinical Trial Administrator: Felicia Frost; ICTU Quality Assurance (QA): Head of QA: Eloise Britten; QA Administrator: Jonathan Dao; ICTU Statistics: Annie Wright (SAP Development only), Lorna Hazell, Leila Janani, Victoria Cornelius; ICL Software Engineer: Christophe AT Stevens; ICTU Research Fellows: Julia Brandts, Fotios Barkas, Nandita Kaza, Ahmed Mohamed; ICTU Clinical Data Systems: Francesco Lala, Nayan Das, Smita Das, Lee Barker, Vanaja Kakarla; Trial Data Manager: Safa Anwar

*Italy Trial Management:*

Hippocrates Research CRO

*Spain Trial Management:*

La Paz ARO

**Sites and Principal Investigators:**

*United Kingdom:*

Dr Rasha Al-Lamee, Hammersmith Hospital, Imperial College Healthcare NHS Trust (SoC, Tertiary care)

Prof. Afzar Zaman, Freeman Hospital, Newcastle upon Tyne Hospitals NHS Foundation Trust (DSS, Tertiary care)

Dr Joe Martins, Russell’s Hall Hospital, Dudley Group NHS Foundation Trust (SoC, Secondary care)

Dr Sharad Agrawal, Sunderland Royal Hospital, South Tyneside and Sunderland NHS Foundation Trust (SoC, Secondary care)

Dr Daniel McKenzie, Royal United Hospital, Royal United Hospitals Bath NHS Foundation Trust (DSS, Secondary care)

Dr Christina Elorz, Conquest Hospital, East Sussex Healthcare NHS Trust (DSS, Secondary care)

Dr Imtiaz Kalyar, Conquest Hospital, East Sussex Healthcare NHS Trust (DSS, Secondary care)

Dr Vivek Kodoth, Royal Bournemouth Hospital, University Hospitals Dorset NHS Foundation Trust (SoC, Mixed care)

Dr Vinoda Sharma, Birmingham City Hospital, Sandwell & West Birmingham NHS Trust (SoC, Secondary care)

Dr Mohammed Alama, Kettering General Hospital, Kettering General Hospital NHS Foundation Trust (SoC, Secondary care)

Dr Sukhbir Dhamrait, Worthing Hospital, University Hospitals Sussex NHS Foundation Trust (DSS, Secondary care)

Dr Azeem Sheikh, Huddersfield Royal Infirmary, Calderdale and Huddersfield NHS Foundation Trust (DSS, Secondary care)

Dr Helen Routledge, Worcestershire Royal Hospital, Worcestershire Acute Hospitals NHS Trust (SoC, Secondary care)

Dr Rashed Hossain, Scunthorpe General Hospital, North Lincolnshire and Goole NHS Foundation Trust (SoC, Secondary care)

Dr Satheesh Balakrishnan-Nair, Glan Glwyd, Betsi Cadwaladr University Health Board (DSS, Mixed care)

Prof. David Ripley, North Tyneside General Hospital, Northumbria NHS Foundation Trust (DSS, Secondary care)

Dr George Hunter, Luton and Dunstable University Hospital, Bedfordshire Hospitals NHS Foundation Trust (DSS, Secondary care)

Dr Andrew Moriarty, Craigavon Area Hospital, HSC Southern Health and Social Care Trust (SoC, Secondary care)

*Italy:*

Prof. Natale Brunetti, A.O.U. Ospedali Riuniti U.O.C. Cardiologia e UTIC (Standard of Care (SoC), Secondary care)

Prof. Raffaele De Caterina, Ospedale di Cisanello - A.U.O.P. Azienda Ospedaliera Universitaria (SoC, Secondary care)

Prof. Giuseppe Boriani, Policlinico di Modena S.C. di Cardiologia (Decision Support System (DSS), Secondary care)

Dott. Claudio Fresco, Azienda Ospedaliero Universitaria (DSS, Secondary care)

Dott.ssa Roberta Della Bona, IRCCS Ospedale Policlinico San Martino (SoC, Secondary care)

Prof. Giuseppe Andò, Azienda Ospedaliera Universitaria Policlinico Gaetano Martino U.O. Cardiologia (DSS, Secondary care)

Prof. Paolo Calabrò, Azienda Ospedaliera di Rilievo Nazionale (A.O.R.N.) “Sant’Anna e San Sebastiano” di Caserta (SoC, Secondary care)

Dott. Alessandro Sciahbasi, Ospedale Sandro Pertini - ASL Roma 2 (DSS, Secondary care)

Dott. Alessandro Navazio, Arcispedale Santa Maria Nuova - Azienda USL/IRCCS Reggio Emilia (SoC, Secondary care)

Dott. Gianni Casella, AUSL di Bologna-Ospedale Maggiore (DSS, Secondary care)

Dott. Andrea Borin, IRCCS Policlinico San Donato (SoC, Mixed care)

*Spain:*

Dr Raul Moreno, Hospital Universitario La Paz (SoC, Tertiary care)

Dr Jose Ramón González Juanatey, Hospital Clínico Universitario Santiago de Compostela (DSS, Mixed care)

Dr Alessandro Sionis, Hospital Sant Pau (DSS, Mixed care)

Dr Alejandro Villanueva, Hospital Universitario Rey Juan Carlos (DSS, Secondary care)

Dr Jesús Peteiro, Hospital A Coruña (SoC, Tertiary care)

Dr Carlos Arellano, Hospital Puerta de Hierro (SoC, Tertiary care)

Dr Jordi Lozano, Hospital Vall d'Hebron (DSS, Tertiary care)

Dr José Tuñón, Hospital Fundación Jiménez Díaz (SoC, Tertiary care)

Dr Manuel Martínez Sellés, Hospital Gregorio Marañón (DSS, Tertiary care)

Dr Roberto Martín, Hospital Universitario La Luz Quiron (SoC, Mixed care)

Dr Jose Lopez Aguilera, Hospital Reina Sofía (SoC, Mixed care)

Dr Francisco Javier Cortés, Hospital Virgen Macarena (SoC, Secondary care)

Dr Leire Unzué, Hospital HM Montepríncipe (DSS, Mixed care)

Dr Domingo Pascual, Hospital Virgen Arrixaca (SoC, Mixed care)

# Supplementary Methods

## Supplementary Method 1: CONSORT 2010 checklist of information to include when reporting a cluster randomised trial

The CONSORT 2010 statement extension for cluster randomized controlled trials was published by Campbell MK, Piaggio G, Elbourne DR, and Altman DG (BMJ. 2012;345:e5661)

| **Section/topic and item No** | **Standard checklist item Extension for cluster designs** | | | **Page No*** |
| --- | --- | --- | --- | --- |
| **Title and abstract** |  | | |  |
| 1a | Identification as a randomised trial in the title Identification as a cluster randomised trial in the title | | | Title (p1) |
| 1b | Structured summary of trial design, methods, results, and conclusions See table 2 (for specific guidance see CONSORT for abstracts)^1112^ | | | Abstract (p1) |
| **Introduction** |  | | |  |
| Background and objectives: |  | | |  |
| 2a | Scientific background and explanation of rationale Rationale for using a cluster design | | | Intro (p3) |
| 2b | Specific objectives or hypotheses Whether objectives pertain to the cluster level, the individual participant level, or both | | | Ontro (p3-4) |
| **Methods** |  | | |  |
| Trial design: |  | | |  |
| 3a | Description of trial design (such as parallel, factorial) including Definition of cluster and description of how the designallocation ratio features apply to the clusters | | | Methods (p4-5) |
| 3b | Important changes to methods after trial commencement (such as eligibility criteria), with reasons | | | Methods (p4-5) |
| Participants: |  | | |  |
| 4a | Eligibility criteria for participants Eligibility criteria for clusters | | | Methods (p4) |
| 4b | Settings and locations where the data were collected | | | Methods (p4-5) |
| Interventions: |  | | |  |
| 5 | The interventions for each group with sufficient details to allow Whether interventions pertain to the cluster level, the replication, including how and when they were actually administered individual participant level, or both | | | Methods (p5) |
| Outcomes: |  | | |  |
| 6a | Completely defined prespecified primary and secondary outcome  Whether outcome measures pertain to the cluster measures, including how and when they were assessed level, the individual participant level, or both | | | Methods (p6) |
| 6b | Any changes to trial outcomes after the trial commenced, with reasons | | | Methods (p6-7) |
| Sample size: |  | | |  |
| 7a | How sample size was determined  Method of calculation, number of clusters(s) (and  whether equal or unequal cluster sizes are assumed),  cluster size, a coefficient of intracluster correlation (ICC or *k*), and an indication of its uncertainty | | | Methods (p7) |
| 7b | When applicable, explanation of any interim analyses and stopping guidelines | | | NA |
| **Randomisation** |  |  | |  |
| Sequence generation: |  |  | |  |
| 8a | Method used to generate the random allocation sequence |  | | Methods (p5) |
| 8b | Type of randomisation; details of any restriction (such as blocking and block size) | Details of stratification or matching if used | | Methods (p5) |
| Allocation concealment mechanism: |  |  | |  |
| 9 | Mechanism used to implement the random allocation sequence (such as sequentially numbered containers), describing any steps taken to conceal the sequence until interventions were assigned | Specification that allocation was based on clusters rather than individuals and whether allocation concealment (if any) was at the cluster level, the individual participant level, or both | | Methods (p5) |
| Implementation: |  |  | |  |
| 10 | Who generated the random allocation sequence, who enrolled participants, and who assigned participants to interventions | Replaced by 10a, 10b, and 10c | | Methods (p5) |
| 10a |  | Who generated the random allocation sequence, who enrolled clusters, and who assigned clusters to interventions | |  |
| 10b |  | Mechanism by which individual participants were included in clusters for the purposes of the trial (such as complete enumeration, random sampling) | |  |
| 10c |  | From whom consent was sought (representatives of the cluster, or individual cluster members, or both) and whether consent was sought before or after randomisation | |  |
| Blinding: |  |  | |  |
| 11a | If done, who was blinded after assignment to interventions (for example, participants, care providers, those assessing outcomes) and how |  | | Methods (p5) |
| 11b | If relevant, description of the similarity of interventions |  | | NA |
| Statistical methods: |  |  | |  |
| 12a | Statistical methods used to compare groups for primary and secondary outcomes | How clustering was taken into account | | Methods (p7) |
| 12b | Methods for additional analyses, such as subgroup analyses and adjusted analyses |  | | Methods (p7) + Suppl. Material (SAP) |
| **Results** |  |  | |  |
| Participant flow (a diagram is strongly recommended): | |  | |  |
| 13a | For each group, the numbers of participants who were randomly assigned, received intended treatment, and were analysed for the primary outcome | For each group, the numbers of clusters that were randomly assigned, received intended treatment, and  were analysed for the primary outcome | | Results (p8-9) + Figure 2 |
| 13b | For each group, losses and exclusions after randomisation, together For each group, losses and exclusions for both  with reasons clusters and individual cluster members | | | Results (p8-9) + Figure 2 |
| Recruitment: |  |  | |  |
| 14a | Dates defining the periods of recruitment and follow-up |  | | Results (p8/9) |
| 14b | Why the trial ended or was stopped |  | | Results (p8/9) |
| Baseline data: |  |  | |  |
| 15 | A table showing baseline demographic and clinical characteristics for each group | Baseline characteristics for the individual and cluster levels as applicable for each group | | Table 1 |
| Numbers analysed: |  |  | |  |
| 16 | For each group, number of participants (denominator) included in each analysis and whether the analysis was by original assigned groups | For each group, number of clusters included in each analysis | | Results text and figures |
| Outcomes and estimation: |  |  | |  |
| 17a | For each primary and secondary outcome, results for each group, and the estimated effect size and its precision (such as 95% confidence interval) | Results at the individual or cluster level as applicable and a coefficient of intracluster correlation (ICC or *k*) for each primary outcome | | Results text and figures |
| 17b | For binary outcomes, presentation of both absolute and relative effect sizes is recommended |  | | Results text and figures |
| Ancillary analyses: |  |  | |  |
| 18 | Results of any other analyses performed, including subgroup analyses and adjusted analyses, distinguishing prespecified from exploratory |  | | Results (p9-11) |
| Harms: |  |  | |  |
| 19 | All important harms or unintended effects in each group (for specific guidance see CONSORT for harms106) | | | Results (p8) / NA |
| **Discussion** |  | | |  |
| Limitations: |  | | |  |
| 20 | Trial limitations, addressing sources of potential bias, imprecision, and, if relevant, multiplicity of analyses | | | Discussion (p11-14) |
| Generalisability: |  | | |  |
| 21 | Generalisability (external validity, applicability) of the trial findings Generalisability to clusters and/or individual participants (as relevant) | | | Discussion (p11-14) |
| Interpretation: |  | |  |  |
| 22 | Interpretation consistent with results, balancing benefits and harms, and considering other relevant evidence | |  | Discussion (p11-14) |
| **Other information** |  | |  |  |
| Registration: |  | |  |  |
| 23 | Registration number and name of trial registry | |  | Abstract and Methods (study design) |
| Protocol: |  | |  |  |
| 24 | Where the full trial protocol can be accessed, if available | |  | Suppl. Material |
| Funding: |  | |  |  |
| 25 | Sources of funding and other support (such as supply of drugs), role of funders | |  | Abstract and Methods (funding section) |
| *Page numbers optional depending on journal requirements. | | |  |  |

## Supplementary Method 2: Full List of Inclusion and Exclusion Criteria

Sites were required to have reliable internet access, the capability to use DSS and a willingness and able to undertake training for the DSS. There were no restrictions on the LLLT use, provided treatment adhered to national guidelines and reimbursement policies. Sites also needed record or access to the following patient information: lipid profiles, LLT at the time of LDL-C measurement, estimated GFR (eGFR), demographics, prior ASCVD, coronary artery disease, cerebrovascular disease, abdominal aortic aneurysm, peripheral artery disease, diabetes mellitus, systolic blood pressure, and smoking status. Sites unable to capture or provide data on patients with ACS during admission and the 16-week follow-up period, or unable or unwilling to use non-statin LLTs were excluded.

Adults aged 18 to 79 years admitted to a trial site were eligible if they experienced ACS, defined as myocardial ischemia symptoms at rest or with minimal exertion within 72 hours of unscheduled admission due to presumed or proven obstructive coronary disease, or had elevated cardiac biomarkers or ischemic ECG changes. Participants needed to be willing to take lipid-lowering therapies (LLTs) for secondary cardiovascular prevention and attend follow-up at the same trial site or clinical team over 16 weeks. Exclusion criteria included inability to provide written informed consent or having a baseline LDL-C measurement below 1.8 mmol/L.

## Supplementary Method 3: Description of the Decision Support System

The Decision Support System (DSS) used in the trial is a web-based application accessible online to prescribing clinicians. It is designed to provide personalized, evidence-based visualization of the risk of atherosclerotic cardiovascular disease (ASCVD) and the potential benefits of lipid-lowering therapy (LLT) optimization. The DSS estimates the 10-year risk of ASCVD recurrence using the SMART risk score and projects this risk over 30 years.

The DSS incorporates a time-dependent cardiovascular treatment benefit model, as described in the article by Khan et al. (2020) in the Journal of the American Heart Association. This model uses data from 22 randomized controlled trials to summarize the relationship between LDL-C lowering and cardiovascular risk reduction. It includes parameters such as the time since treatment initiation, the magnitude of LDL-C reduction, and additional patient characteristics. The model accurately predicts treatment benefits by incorporating patient profiles, treatment types, and durations.

Using this model, the DSS calculates the clinical benefit, in terms of risk reduction, for various LLT regimens selected by the user, including monotherapy and combination therapies. It shows the expected clinical benefit of therapies selected by the clinicians (i.e. it does not make recommendation), supporting clinical decision-making in line with the guidelines of the European Atherosclerosis Society (EAS) and the European Society of Cardiology (ESC). The DSS aims to facilitate a patient-specific assessment, helping clinicians make informed decisions about LLT strategies and communicate the clinical value of continued therapy to patients. Sites randomized to the DSS group received standardized training on the use of the DSS tool.

The DSS enables users to impute high-sensitivity C-Reactive Protein (hsCRP) values using the eTable 1 from McKay et al. (European Journal of Preventive Cardiology, 2022). This imputation feature is essential as hsCRP is a required input for both the SMART risk score and the benefit calculator, despite being rarely measured in routine clinical practice.

### Technical Aspects of the DSS


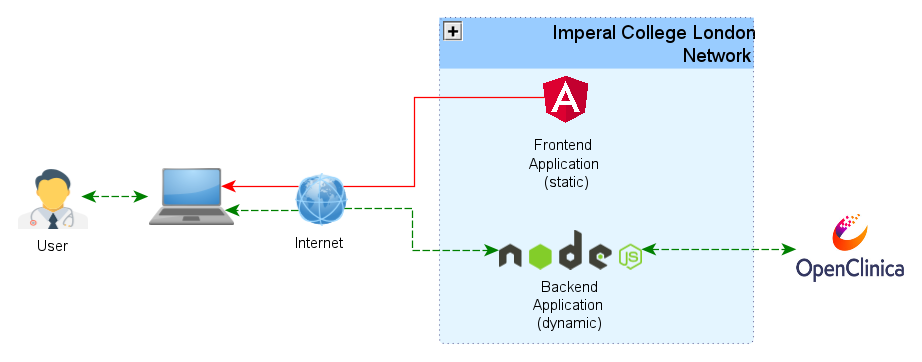


**Figure: Diagrams of communication between the end user, Imperial College London and OpenClinica. The red arrow is unidirectional. The green arrow is bidirectional.**

The Decision Support System (DSS) is composed of two main software components: a **front end** and a **back end**. The front end is responsible for the graphical interface and includes key functionalities, allowing users to interact with the system seamlessly. The back end, on the other hand, manages the features required to push data into the database and retrieve (pull) data from it, ensuring efficient secure, communication with the underlying data sources.

When users visit <https://zodiac.study>, the front end application, built using the Angular framework, is loaded into their web browser (e.g., Mozilla Firefox, Microsoft Edge, Google Chrome, Safari). This application runs directly in the user's browser and communicates with the back end, which is a Node.js-based service.

The back end acts as a proxy between the front end and the OpenClinica system. It processes requests sent by the Angular front end, prepares them, and communicates with the OpenClinica backend via its REST API (version 4). REST stands for Representational State Transfer, a widely used architectural style for designing web services. It allows communication between systems using standardized HTTP methods.

The back end receives responses from OpenClinica, processes and transforms the data, and sends it back to the Angular front end. The front end then uses this information to display the estimated risks and benefits or to manage functionalities such as user authentication. Users inputs were pushed backed from the front end to the study database.

User access security is managed entirely by the OpenClinica system, which serves as the central authentication and authorisation mechanism. This approach ensures that only verified participants with appropriate credentials can access the DSS. User accounts are created within the OpenClinica environment, with specific role-based permissions assigned. This integration provides a robust security framework that maintains data integrity and confidentiality whilst allowing authorised clinicians to utilise the decision support tools. All system interactions are logged and monitored through OpenClinica's comprehensive audit trail capabilities, providing transparency and accountability in line with clinical research standards.

## Supplementary Method 4: Expected percentage low-density lipoprotein cholesterol (LDL-C) reduction used by the DSS and in the calculation of the primary endpoint.

| **Treatment** | **Reduction (%)** | **Source** |
| --- | --- | --- |
| rosuvastatin 5mg | 38 | [Meta-analysis](https://www.bmj.com/content/326/7404/1423.short) |
| rosuvastatin 10mg | 43 | [Meta-analysis](https://www.bmj.com/content/326/7404/1423.short) |
| rosuvastatin 20mg | 48 | [Meta-analysis](https://www.bmj.com/content/326/7404/1423.short) |
| rosuvastatin 40mg | 53 | [Meta-analysis](https://www.bmj.com/content/326/7404/1423.short) |
| atorvastatin 10mg | 37 | [Meta-analysis](https://www.bmj.com/content/326/7404/1423.short) |
| atorvastatin 20mg | 43 | [Meta-analysis](https://www.bmj.com/content/326/7404/1423.short) |
| atorvastatin 40mg | 49 | [Meta-analysis](https://www.bmj.com/content/326/7404/1423.short) |
| atorvastatin 80mg | 55 | [Meta-analysis](https://www.bmj.com/content/326/7404/1423.short) |
| simvastatin 5mg | 23 | [Meta-analysis](https://www.bmj.com/content/326/7404/1423.short) |
| simvastatin 10mg | 27 | [Meta-analysis](https://www.bmj.com/content/326/7404/1423.short) |
| simvastatin 20mg | 32 | [Meta-analysis](https://www.bmj.com/content/326/7404/1423.short) |
| simvastatin 40mg | 37 | [Meta-analysis](https://www.bmj.com/content/326/7404/1423.short) |
| simvastatin 80mg | 42 | [Meta-analysis](https://www.bmj.com/content/326/7404/1423.short) |
| fluvastatin 20mg | 21 | [Meta-analysis](https://www.bmj.com/content/326/7404/1423.short) |
| fluvastatin 40mg | 27 | [Meta-analysis](https://www.bmj.com/content/326/7404/1423.short) |
| fluvastatin 80mg | 33 | [Meta-analysis](https://www.bmj.com/content/326/7404/1423.short) |
| lovastatin 10mg | 21 | [Meta-analysis](https://www.bmj.com/content/326/7404/1423.short) |
| lovastatin 20mg | 29 | [Meta-analysis](https://www.bmj.com/content/326/7404/1423.short) |
| lovastatin 40mg | 37 | [Meta-analysis](https://www.bmj.com/content/326/7404/1423.short) |
| lovastatin 80mg | 45 | [Meta-analysis](https://www.bmj.com/content/326/7404/1423.short) |
| pravastatin 10mg | 20 | [Meta-analysis](https://www.bmj.com/content/326/7404/1423.short) |
| pravastatin 20mg | 24 | [Meta-analysis](https://www.bmj.com/content/326/7404/1423.short) |
| pravastatin 40mg | 29 | [Meta-analysis](https://www.bmj.com/content/326/7404/1423.short) |
| pravastatin 80mg | 33 | [Meta-analysis](https://www.bmj.com/content/326/7404/1423.short) |
| pitavastatin 1mg | 33.3 | [SmPC](https://kowapharmaceuticals.eu/wp-content/uploads/Livazo-1mg-2mg-4mg-SmPC-04.12.2020.pdf) |
| pitavastatin 2mg | 38.2 | [SmPC](https://kowapharmaceuticals.eu/wp-content/uploads/Livazo-1mg-2mg-4mg-SmPC-04.12.2020.pdf) |
| pitavastatin 4mg | 46.5 | [SmPC](https://kowapharmaceuticals.eu/wp-content/uploads/Livazo-1mg-2mg-4mg-SmPC-04.12.2020.pdf) |
| ezetimibe 10mg | 24.64 | [Network Meta-analysis](https://www.ahajournals.org/doi/10.1161/JAHA.122.025551) |
| alirocumab 75mg | 53.32 | [Network meta-analysis](https://www.ahajournals.org/doi/10.1161/JAHA.122.025551) |
| alirocumab 150mg | 62.71 | [Network Meta-analysis](https://www.ahajournals.org/doi/10.1161/JAHA.122.025551) |
| evolocumab 140mg | 64.73 | [Network Meta-analysis](https://www.ahajournals.org/doi/10.1161/JAHA.122.025551) |
| evolocumab 420mg | 64.73 | [Network Meta-analysis](https://www.ahajournals.org/doi/10.1161/JAHA.122.025551) |
| inclisiran 284mg injection | 50.17 | [Network Meta-analysis](https://www.ahajournals.org/doi/10.1161/JAHA.122.025551) |
| bempedoic acid 180mg | 23.4 | [Pooled RCT/SLR](https://www.ahajournals.org/doi/full/10.1161/JAHA.119.016262) |
| any bile acid sequestrant | 16.2 | [Meta-analysis](https://www.amjmed.com/article/S0002-9343(20)30372-7/fulltext) |

## Supplementary Method 5: Description of the Lipid-Lowering Therapy Potency Categories

The intensity of lipid lowering therapies, both mono and combination therapies, was defined based on the table below. Intensification

| Intensity | ZODIAC STUDY Categories BASED on 2019 ESC/EAS GUIDELINE | List of MONOTHERAPIES and POSSIBILITY OF COMBINATION THERAPIES |
| --- | --- | --- |
| Low | [ 0%, 30%] | **Monotherapies:** any bile acid sequestrant, pravastatin 10mg, fluvastatin 20mg, lovastatin 10mg, simvastatin 5mg, bempedoic acid 180mg, pravastatin 20mg, ezetimibe 10mg, simvastatin 10mg, fluvastatin 40mg, lovastatin 20mg, pravastatin 40mg  **Combination therapies:** no |
| Medium/MODERATE | [30%, 50%] | **Monotherapies:** simvastatin 20mg,fluvastatin 80mg,pravastatin 80mg,pitavastatin 1mg,atorvastatin 10mg,simvastatin 40mg,lovastatin 40mg,rosuvastatin 5mg,pitavastatin 2mg,simvastatin 80mg,rosuvastatin 10mg,atorvastatin 20mg,lovastatin 80mg,pitavastatin 4mg,rosuvastatin 20mg,atorvastatin 40mg  **Combination therapies:** yes, e.g., simvastatin 5mg+ ezetimibe |
| High | ]50%, 65%] | **Monotherapies:** inclisiran 284mg injection, rosuvastatin 40mg, alirocumab 75mg, atorvastatin 80mg, alirocumab 150mg, evolocumab 140mg, evolocumab 420mg  **Combination therapies:** yes e.g., rosuvastatin 20mg+ ezetimibe |
| VERY HIGH | [65%, 100%] | **Monotherapies:** no  **Combination therapies:** yes e.g., rosuvastatin 40mg+ alirocumab 150mg |

# Supplementary Tables

## Supplementary Table 1: Visit attendance pattern

|  | Trial arm | | |
| --- | --- | --- | --- |
|  | SOC | DSS | Total |
| N | 616 | 523 | 1,139 |
| Number of post-baseline visits per patient, n(%) |  |  |  |
| None | 30 (4.9) | 20 (3.8) | 50 (4.4) |
| 1 | 466 (75.6) | 460 (88.0) | 926 (81.3) |
| 2 | 99 (16.1) | 31 (5.9) | 130 (11.4) |
| 3 or more | 21 (3.4) | 12 (2.3) | 33 (2.9) |
| Time to first visit (days) |  |  |  |
| Mean (SD) | 78 (44) | 91 (44) | 84 (44) |
| Median (IQR) | 70 (43,107) | 90 (53,115) | 82 (46,112) |
| Month of post-baseline visit, n (%) |  |  |  |
| Before Month 1 | 44 (5.9) | 16 (2.9) | 60 (4.6) |
| Month 2 | 210 (28.1) | 122 (21.8) | 332 (25.4) |
| Month 3 | 131 (17.5) | 105 (18.8) | 236 (18.1) |
| Month 4 | 152 (20.3) | 156 (27.9) | 308 (23.6) |
| Later than Month 4 | 210 (28.1) | 161 (28.7) | 371 (28.4) |

## Supplementary Table 2: Sensitivity analysis of the effect of the DSS on proportion of participants initiated on LLT combination therapy, escalation of LLT monotherapy or escalation of LLT combination therapy in participants *with complete outcome data*

| Participants with  primary outcome before 16 weeks | Trial arm | | Adjusted  Risk Ratio^a^ | 95% CI; p value |
| --- | --- | --- | --- | --- |
|  | SOC | DSS |  |  |
| n^b^ (missing) | 603 (6) | 513 (5) | 1.11 | 0.92 to 1.34; 0.282 |
| n^c^ (%) | 396 (65.7) | 368 (71.7) |  |  |

^a^adjusted for country, site type (secondary/ tertiary care/mix of both) and study site

^b^ N=number of participants with outcome data; participants who died are excluded from primary estimand; multiple imputation used for missing outcome data

^c^ n=number of participants meeting endpoint and % of non-missing

## Supplementary Table 3: Sensitivity analysis of the effect of the DSS on proportion of participants initiated on LLT combination therapy, escalation of LLT monotherapy or escalation of LLT combination therapy *inclusive of participants who died (full ITT)*

| **Participants with primary outcome before 16 weeks** | **Trial arm** | | **Adjusted Risk Ratio^a^** | **95% CI; p value** |
| --- | --- | --- | --- | --- |
|  | **SOC** | **DSS** |  |  |
| **N^b^ (missing)** | 605 (11) | 514 (9) | 1.11 | 0.92 to 1.34; 0.289 |
| **n^c^ (%)** | 398 (65.8) | 369 (71.8) |  |  |

^a^adjusted for country, site type (secondary/ tertiary care/mix of both) and study site

^b^ N=number of participants with outcome data; participants who died are excluded from primary estimand; multiple imputation used for missing outcome data

^c^ n=number of participants meeting endpoint and % of non-missing

## Supplementary Table 4: Lipid levels at 16 weeks post-ACS.

|  | **Values at 16 weeks per Arm^*^** | |
| --- | --- | --- |
|  | **SOC** | **DSS** |
| **N** | 616 | 523 |
| **Total Cholesterol (mmol/L)** |  |  |
| N non-missing | 362 | 364 |
| Median (IQR) | 3.0 (2.6, 3.6) | 3.0 (2.5, 3.5) |
| **LDL-C (mmol/L)** |  |  |
| N non-missing | 366 | 367 |
| Median (IQR) | 1.4 (1.1, 1.8) | 1.3 (1.0, 1.8) |
| **HDL-C (mmol/L)** |  |  |
| N non-missing | 357 | 364 |
| Median (IQR) | 1.1 (0.9, 1.3) | 1.1 (0.9, 1.2) |
| **Triglycerides (mmol/L)** |  |  |
| N non-missing | 357 | 364 |
| Median (IQR) | 1.1 (0.9, 1.5) | 1.1 (0.8, 1.5) |
| ^*^ Using closest test result in follow-up period up to Week 16 | | |

## Supplementary Table 5: Responses to the System Usability Scale (SUS) Questionnaire (n=21)

| Statement^a^ | Mean Rating^b^ (SD); range | Agree, n (%) | Disagree, n (%) |
| --- | --- | --- | --- |
| *1.I think that I would like to use this tool frequently.* | 4 (1);2,5 | 15 (71.4) | 2 (9.5) |
| *2.I found the tool unnecessarily complex.* | 4 (1);1,5 | 3 (14.3) | 16 (76.2) |
| *3.I thought the tool was easy to use.* | 4 (1);3,5 | 17 (81.0) | 0 (0.0) |
| *4.I think that I would need the support of a technical person to be able to use* | 5 (1);1,5 | 2 (9.5) | 19 (90.5) |
| *5.I found the various functions in the tool were well integrated.* | 4 (1);1,5 | 13 (61.9) | 3 (14.3) |
| *6.I thought there was too much inconsistency in this tool.* | 4 (1);1,5 | 3 (14.3) | 17 (81.0) |
| *7. I imagine that most people would learn to use this tool very quickly.* | 4 (1);4,5 | 21 (100.0) | 0 (0.0) |
| *8. I found the tool very awkward to use.* | 4 (1);1,5 | 2 (9.5) | 16 (76.2) |
| *9. I felt very confident using the tool.* | 4 (1);2,5 | 19 (90.5) | 1 (4.8) |
| *10. I needed to learn a lot of things before I could get going with this tool.* | 4 (1);1,5 | 3 (14.3) | 17 (81.0) |
| Statement^c^ | Mean Rating (SD),  range | Poor or worse,  n (%) | Good or better,  n (%) |
| 11.Overall, I would rate the user-friendliness of this tool as | 5 (1);3,6 | 0 (0.0) | 19 (90.5) |

^a^ Score from 1 to 5

^b^ Higher score is better; even numbered items have been reverse coded

^c^ Score from 1 to 6

The System Usability Scale (SUS) questionnaire was developed by Brooke J. and introduced in *Usability Evaluation in Industry*, edited by Jordan PW, Thomas B, Weerdmeester BA, and McClelland IL (London: Taylor & Francis; 1996. p. 189-94).

# Supplementary Figures

## Supplementary Figure 1: Screenshot of the estimation of 10-year risk of ASCVD using the SMART calculator in the Decision Support System.

**
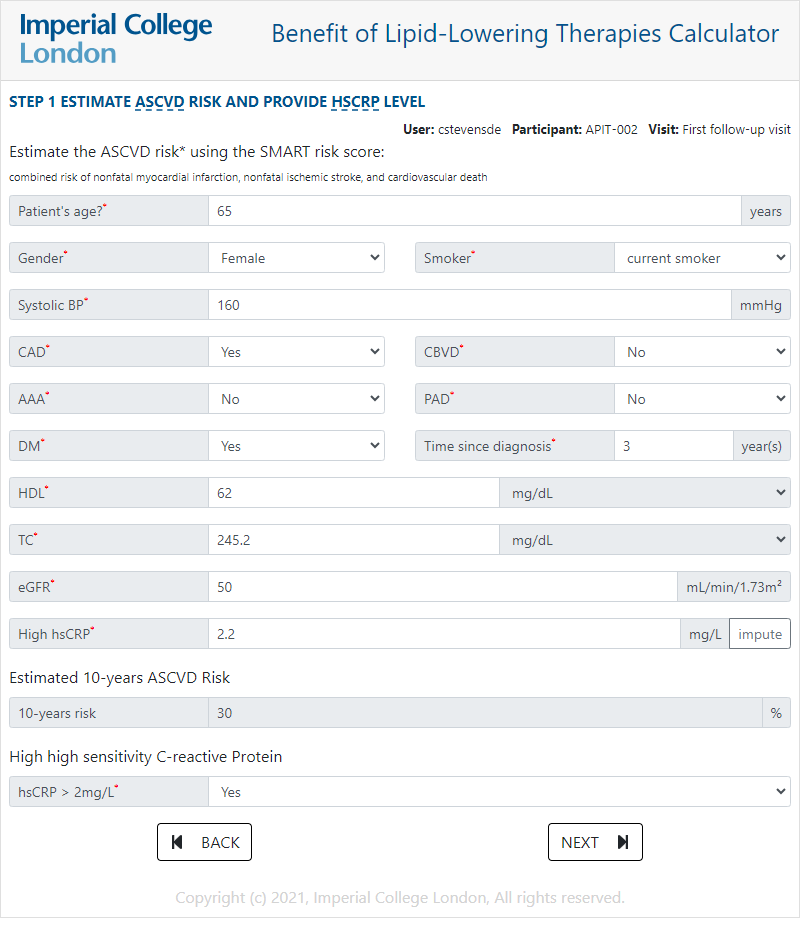
**

## Supplementary Figure 2: Screenshot of the risk trajectories under different Lipid Lowering Treatment (LLT) regimens over the next 30 years, which can help clinicians understand the benefits of changing different LLTs for a given patient using the Decision Support System.


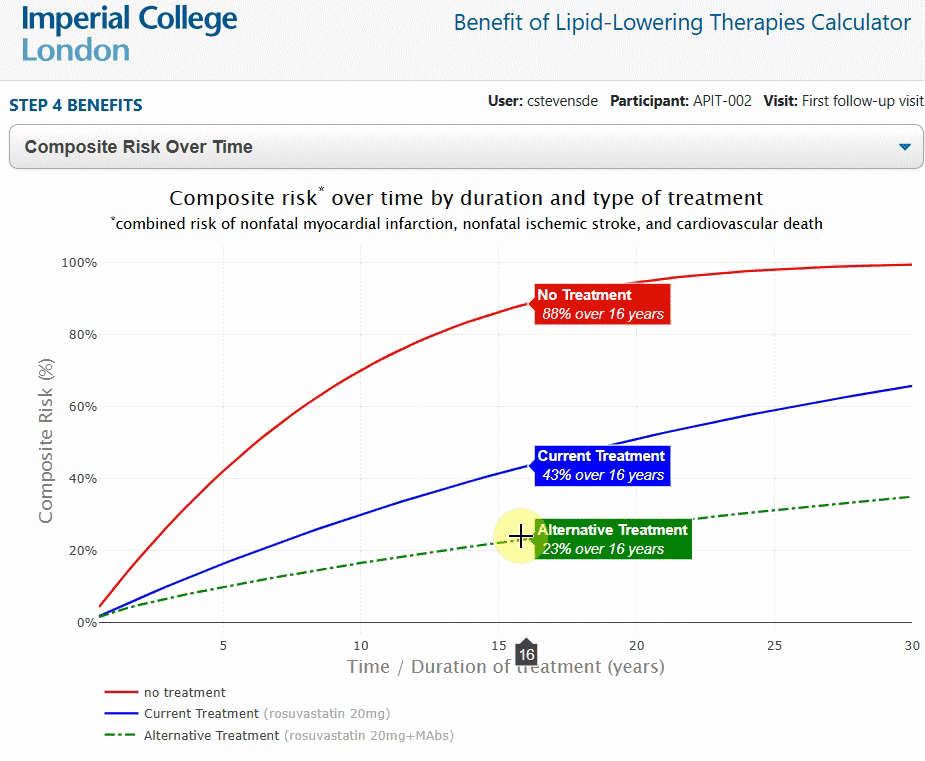


## Supplementary Figure 3: Study design schema.


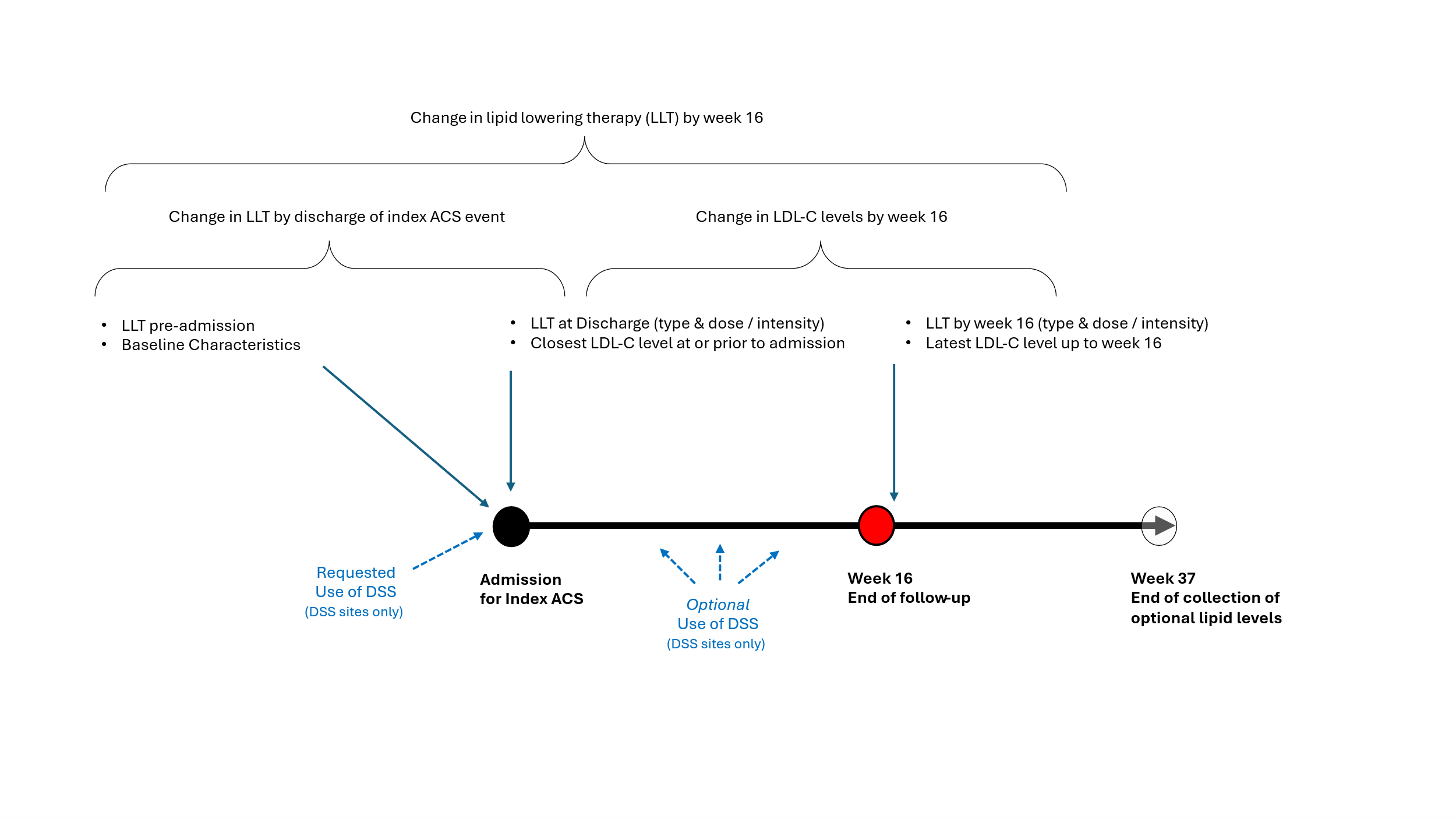


## Supplementary Figure 4: Subgroups analyses of the primary endpoint.

**Abbreviations:** LLT, lipid lowering treatment; CV, cardiovascular; CVD, cardiovascular disease.


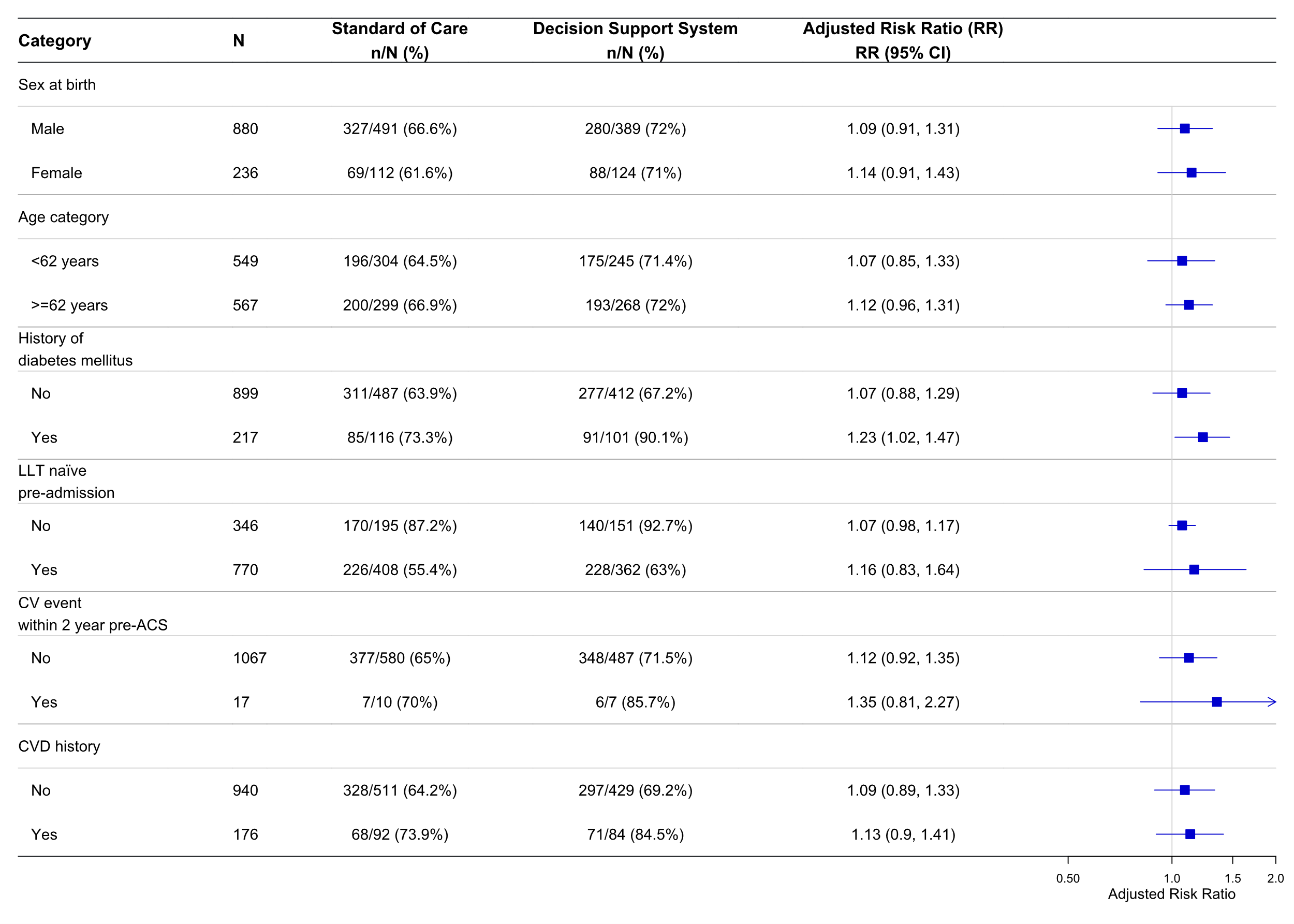


## Supplementary Figure 5: Effect of DSS on separate components of the primary endpoint; adjusted for site, site type and country.

**Abbreviations:** LLT, lipid lowering treatment.


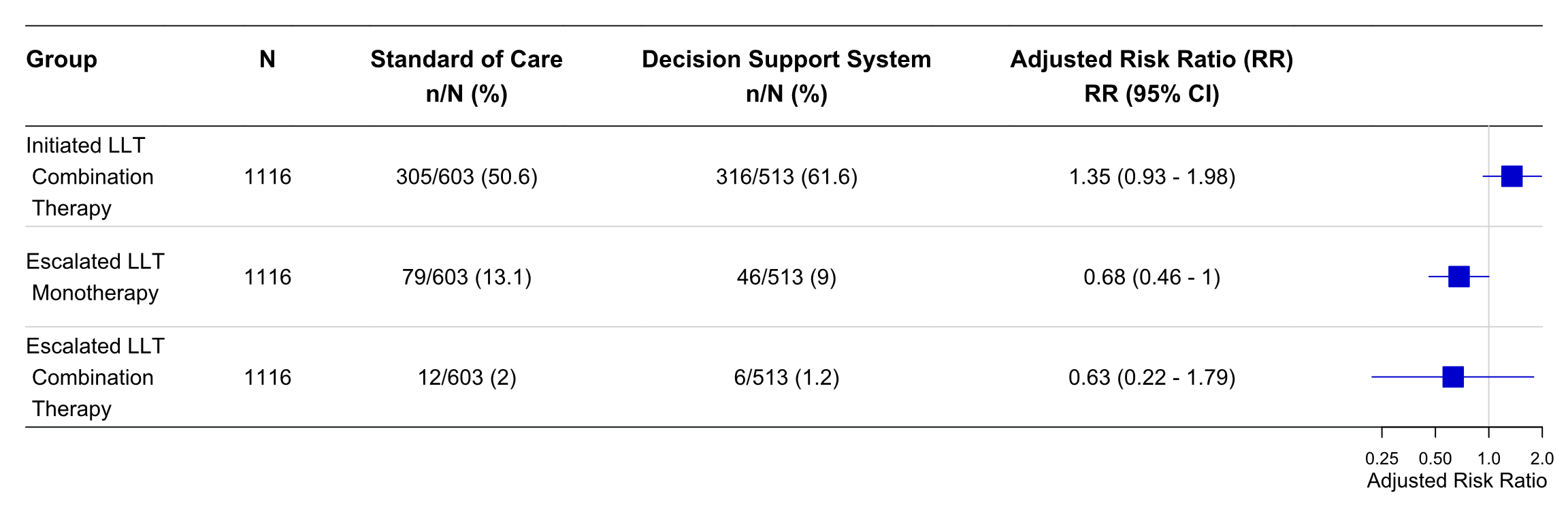


# Supplementary Documents

## Supplementary Document 1: Protocol


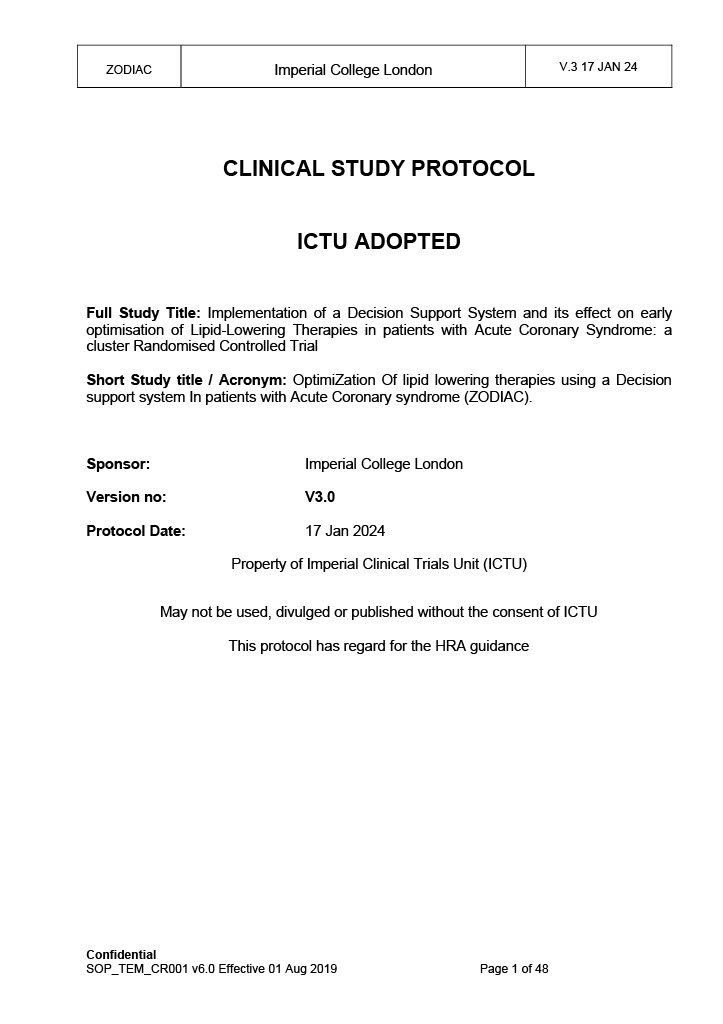


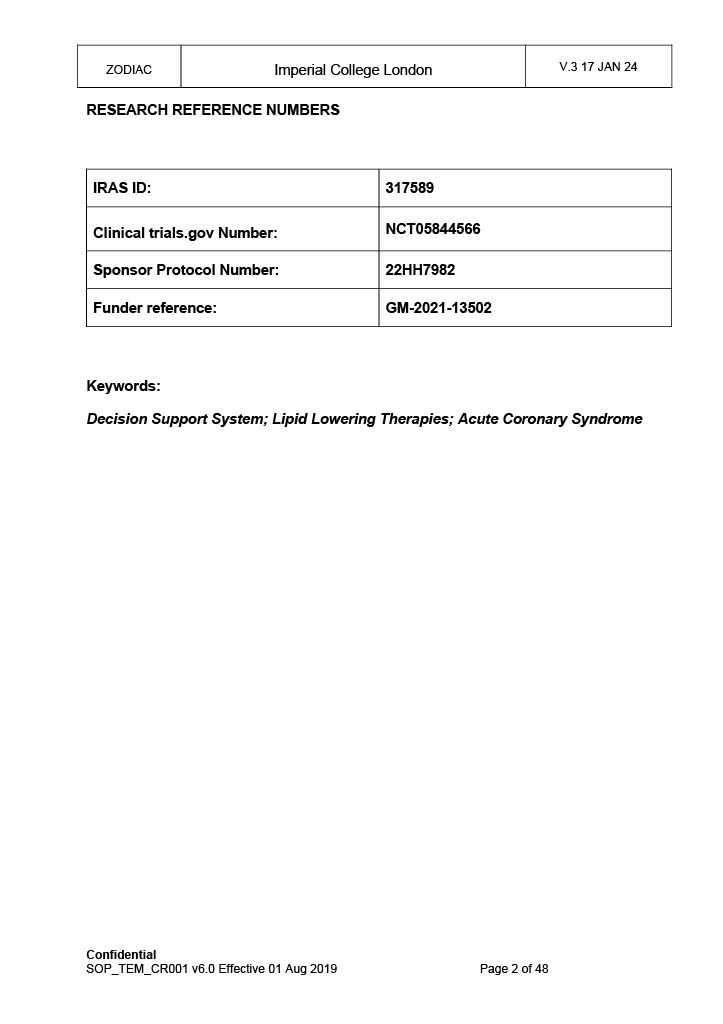

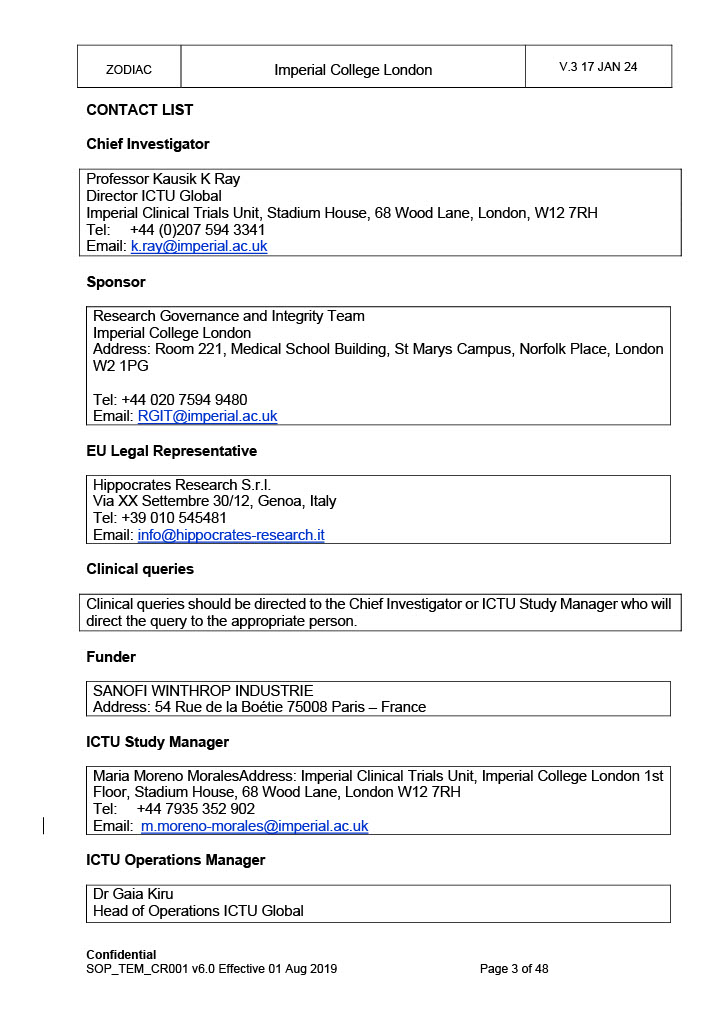

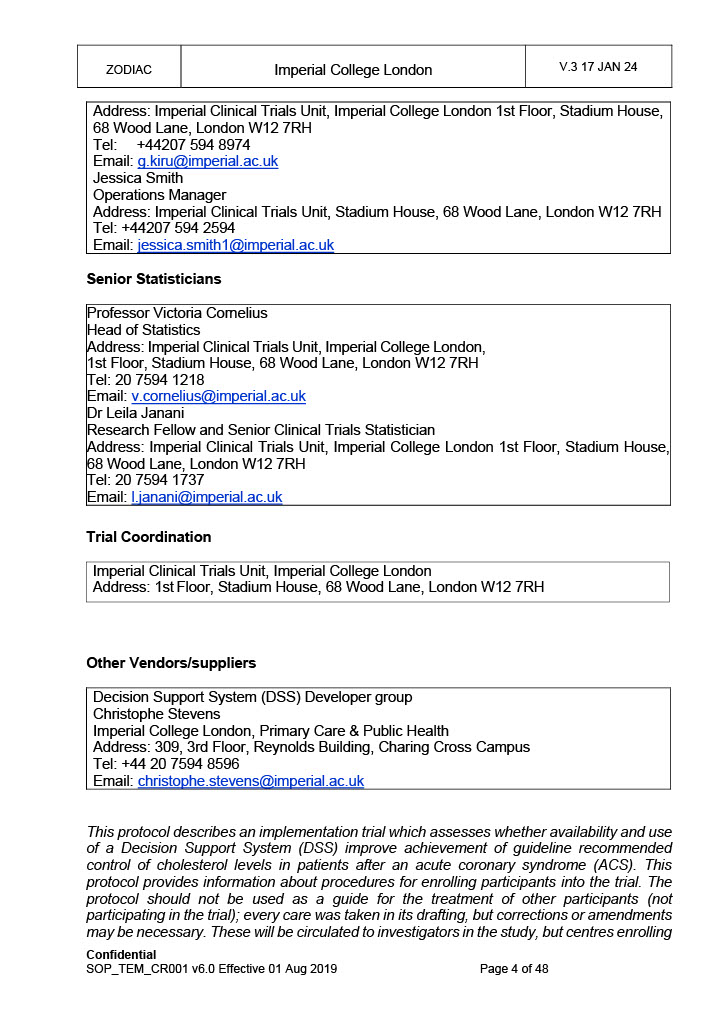

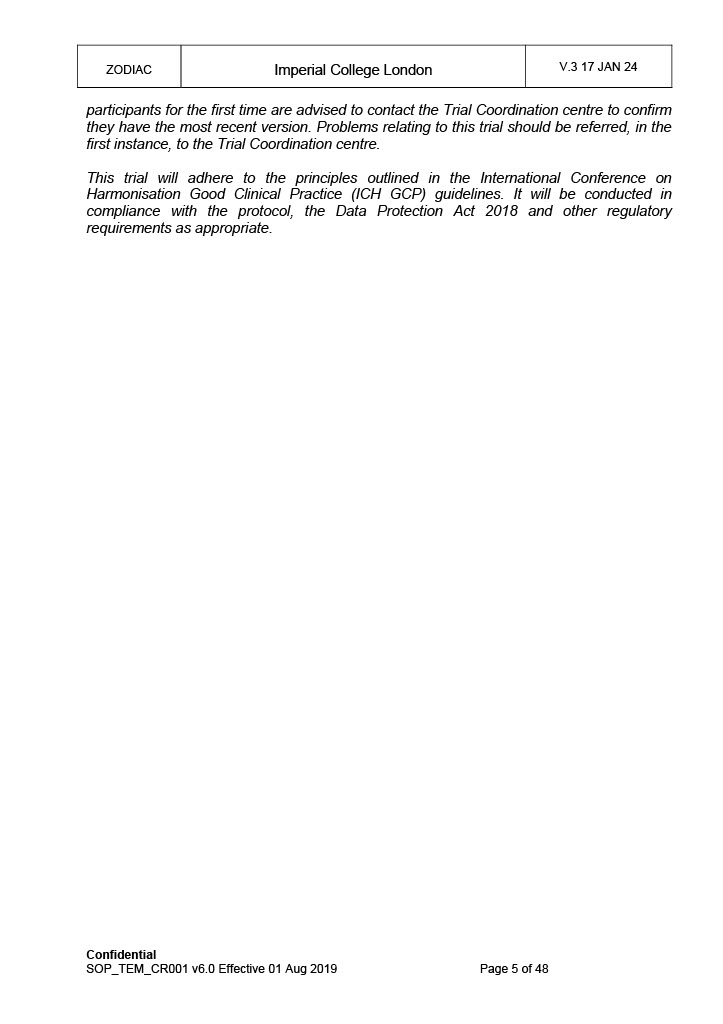

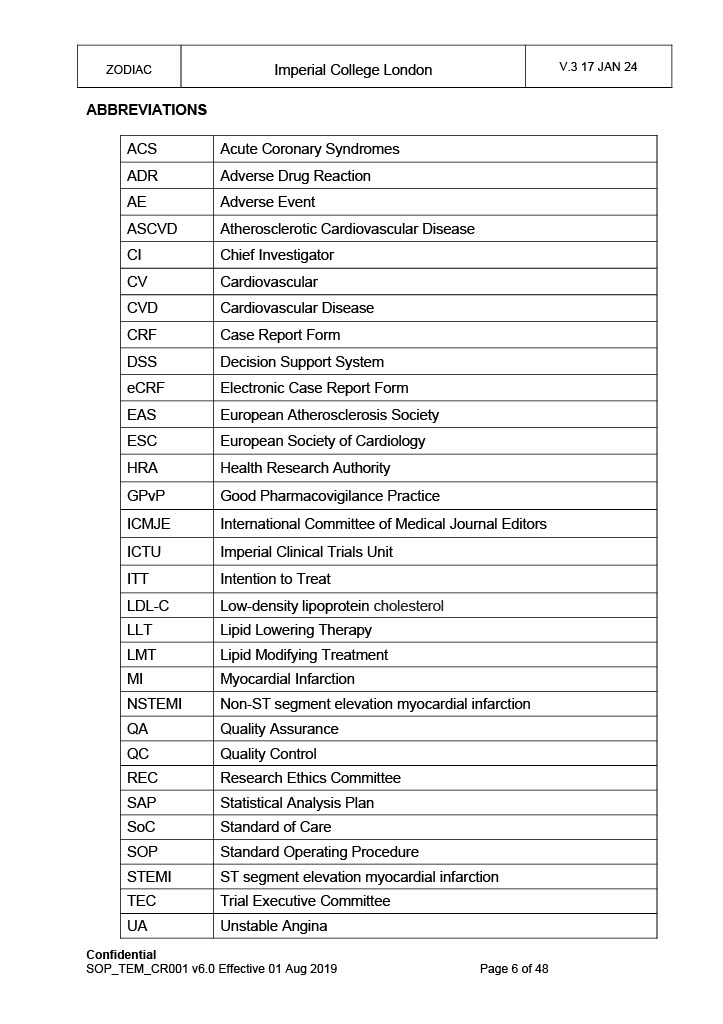

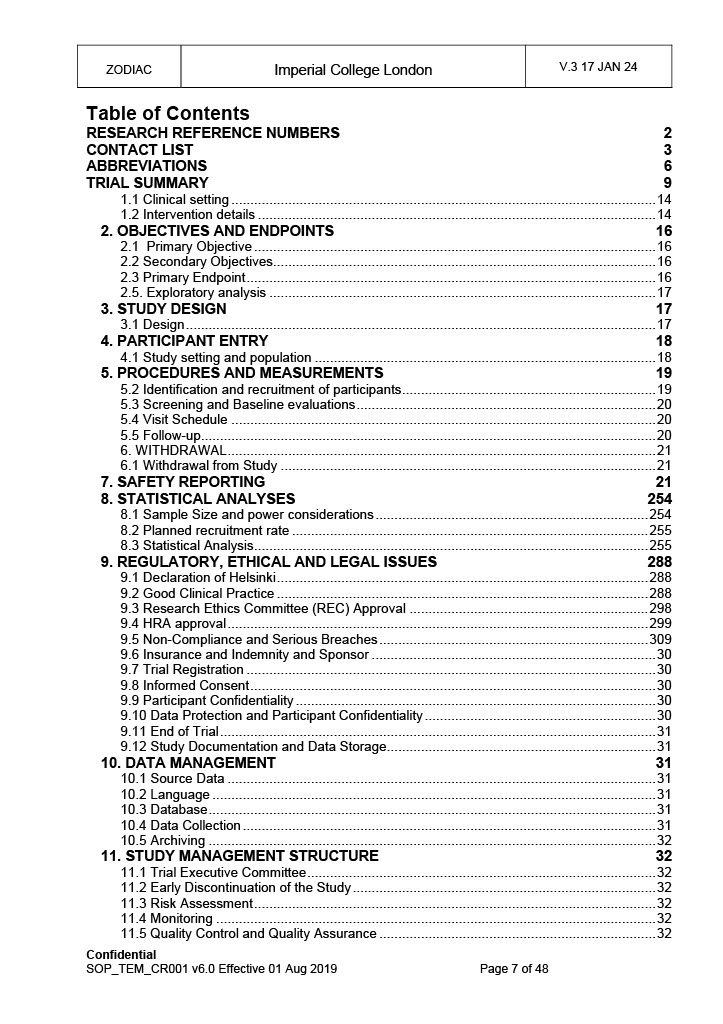

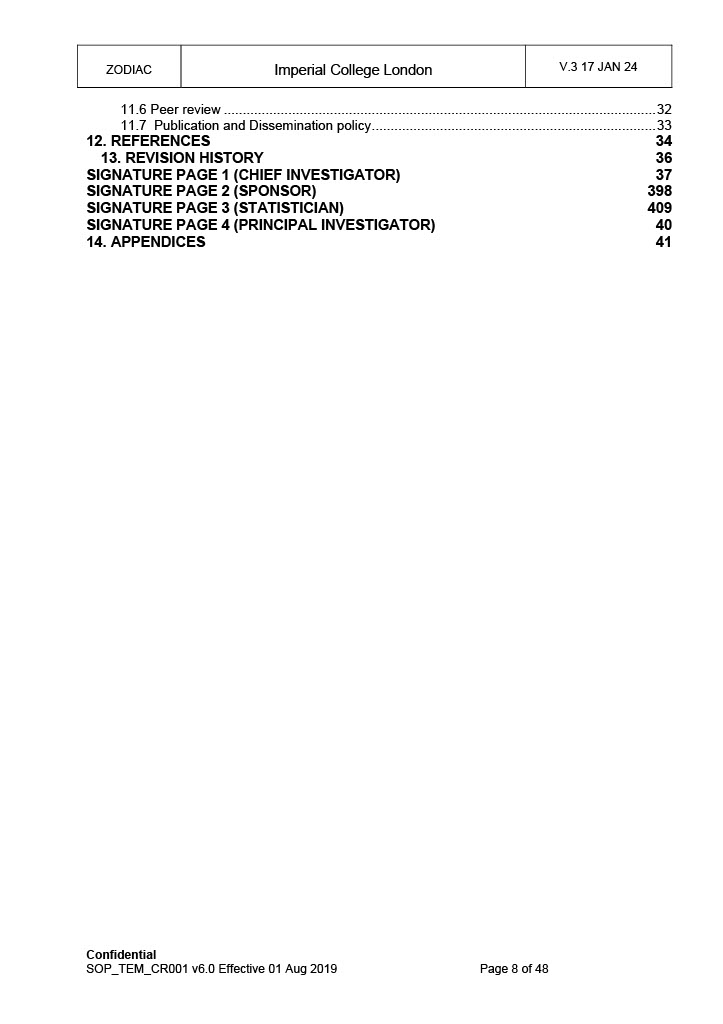

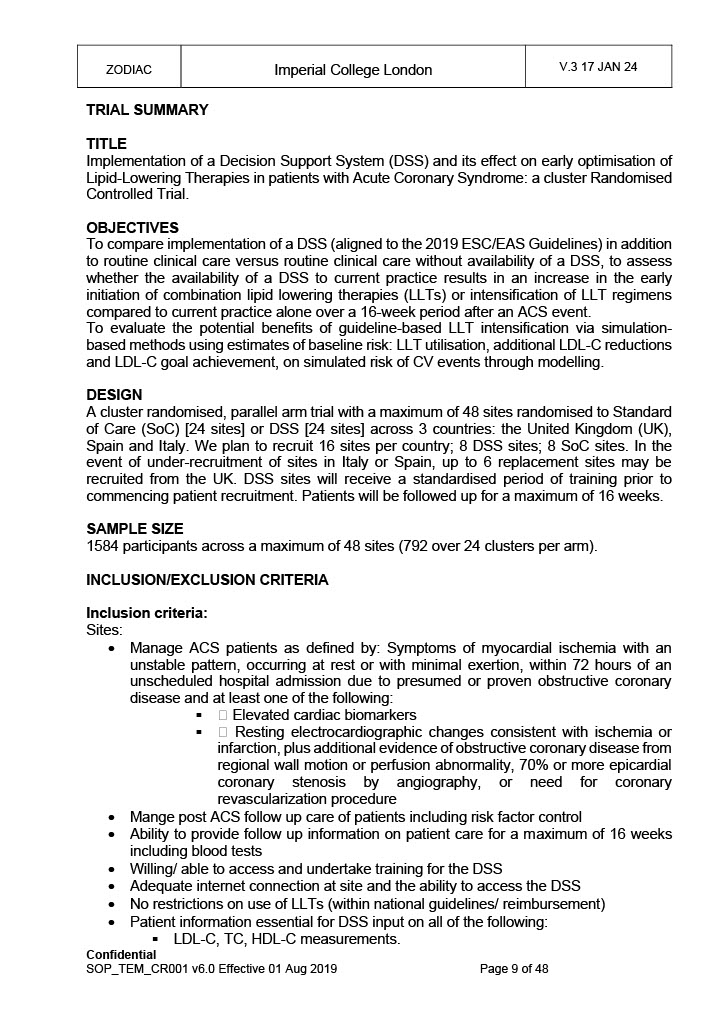

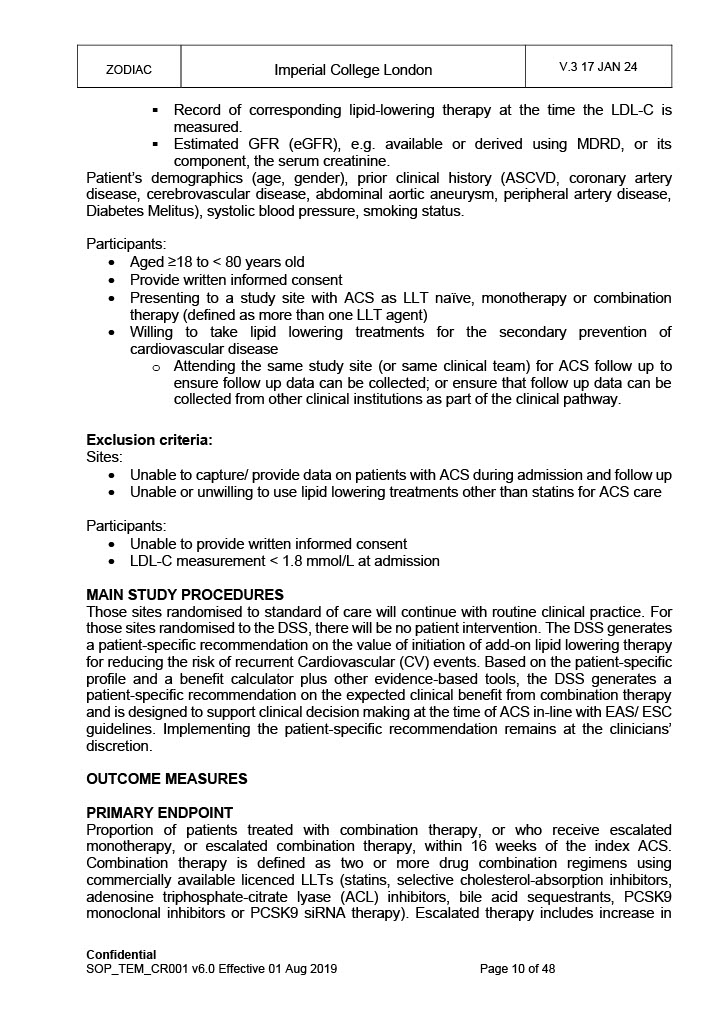

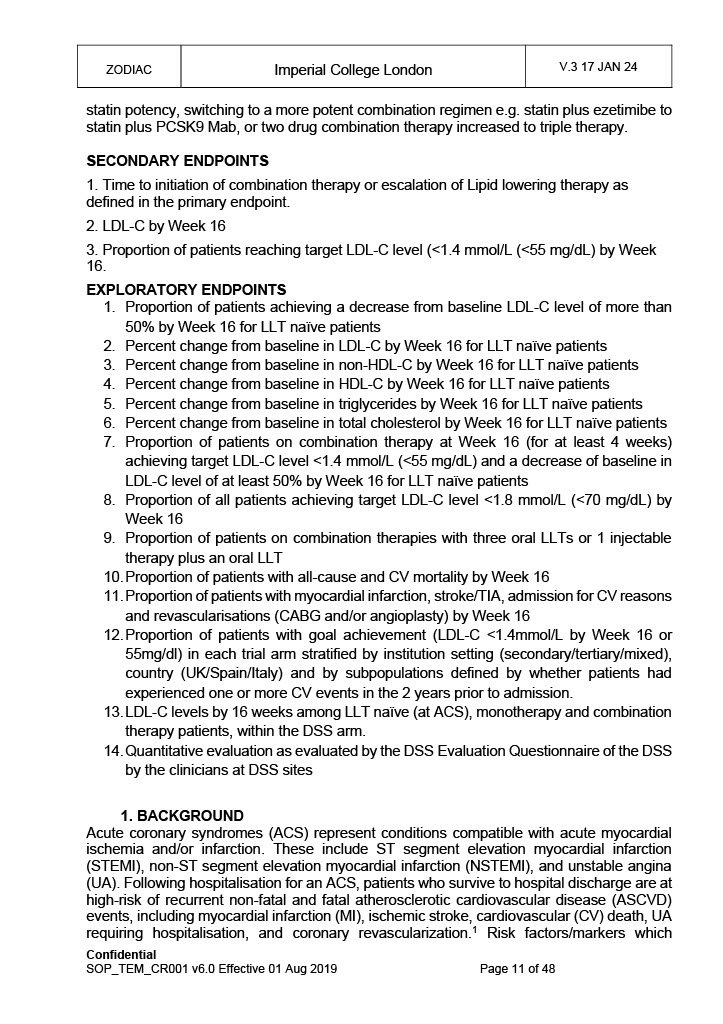

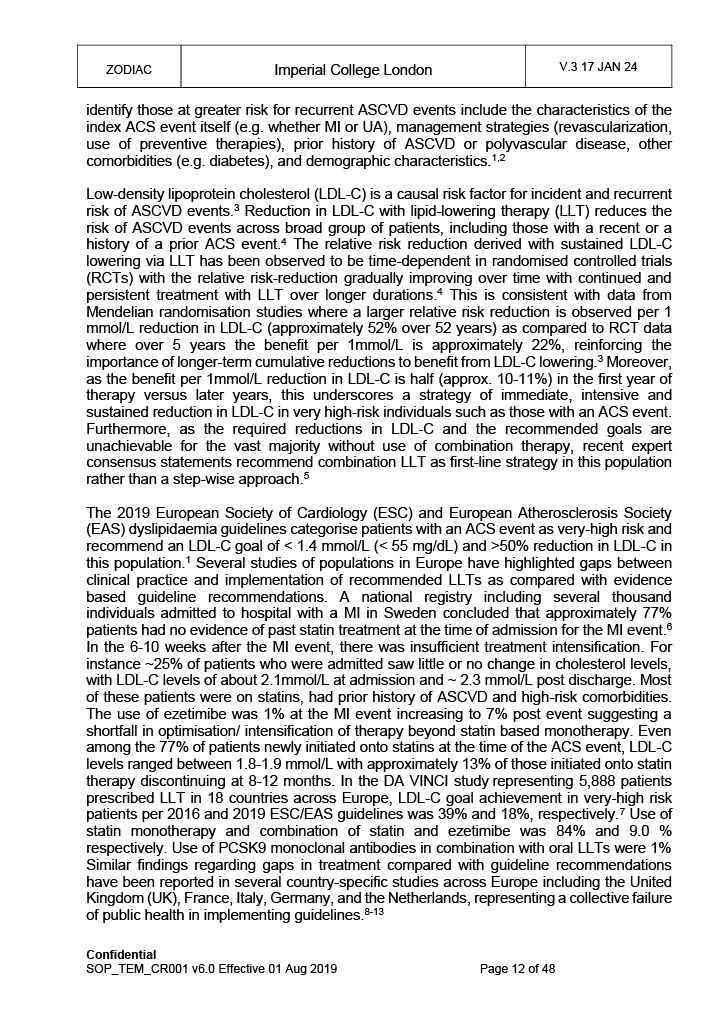

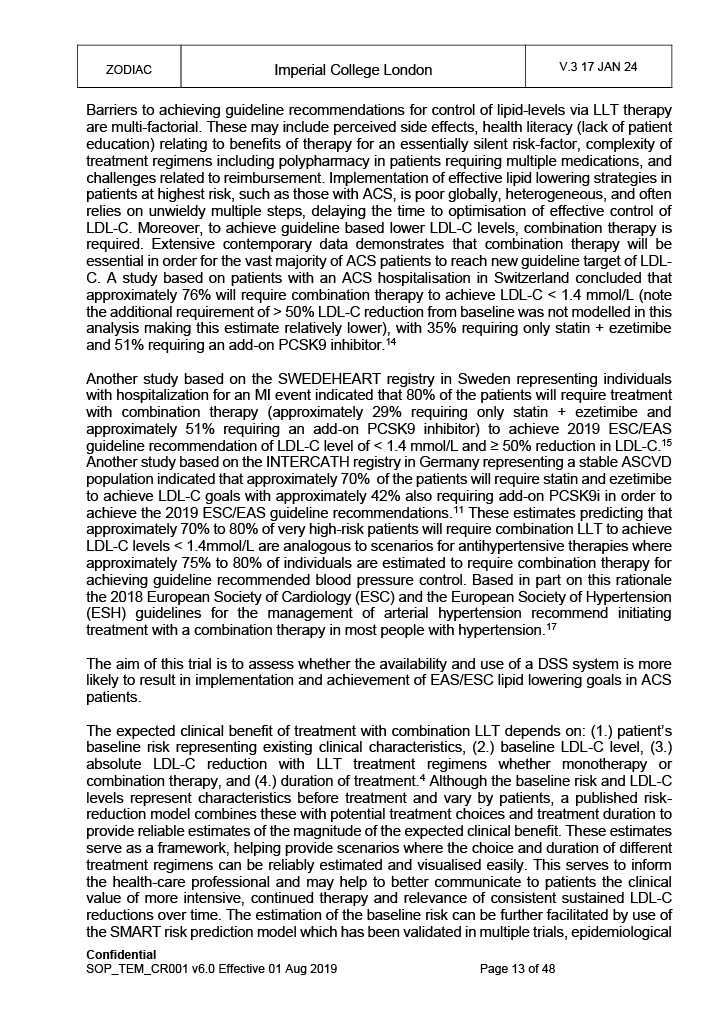

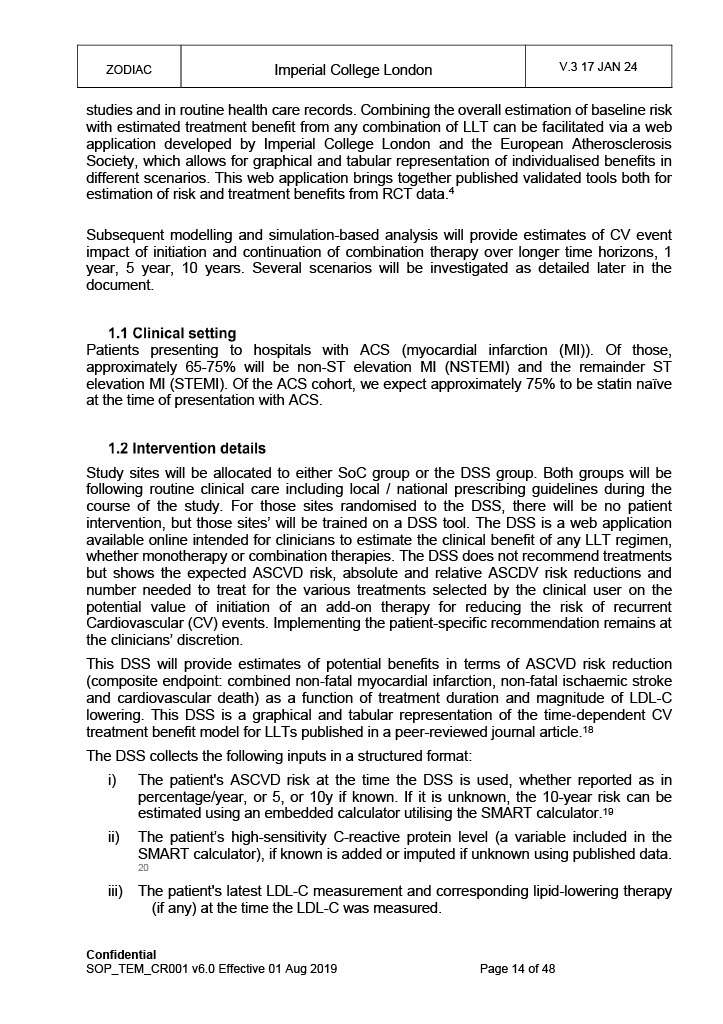

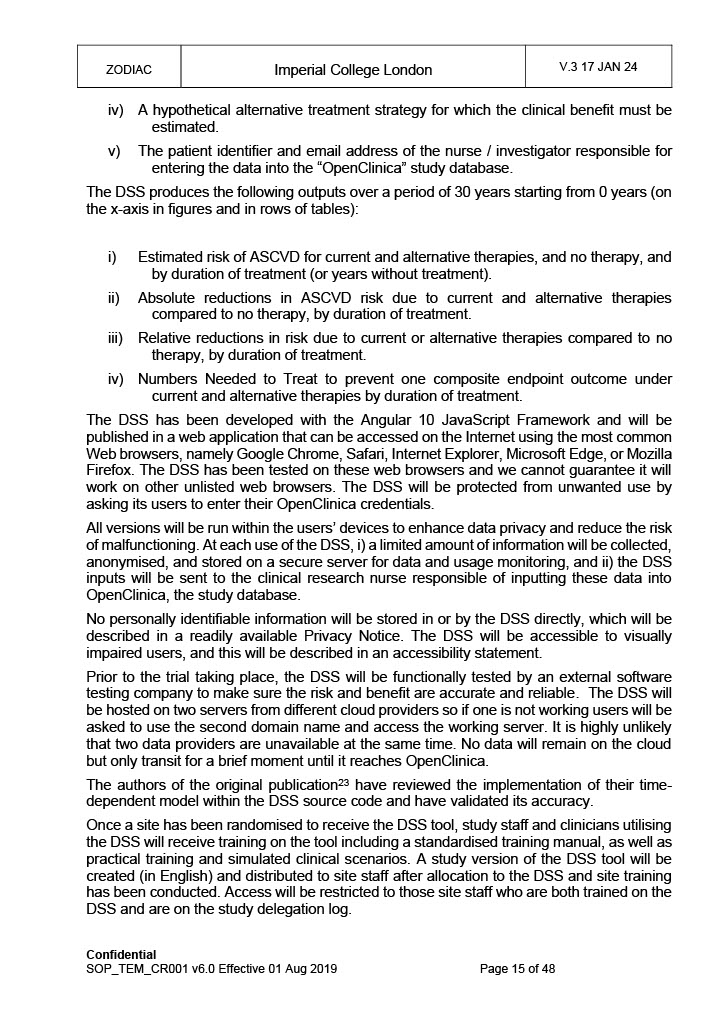

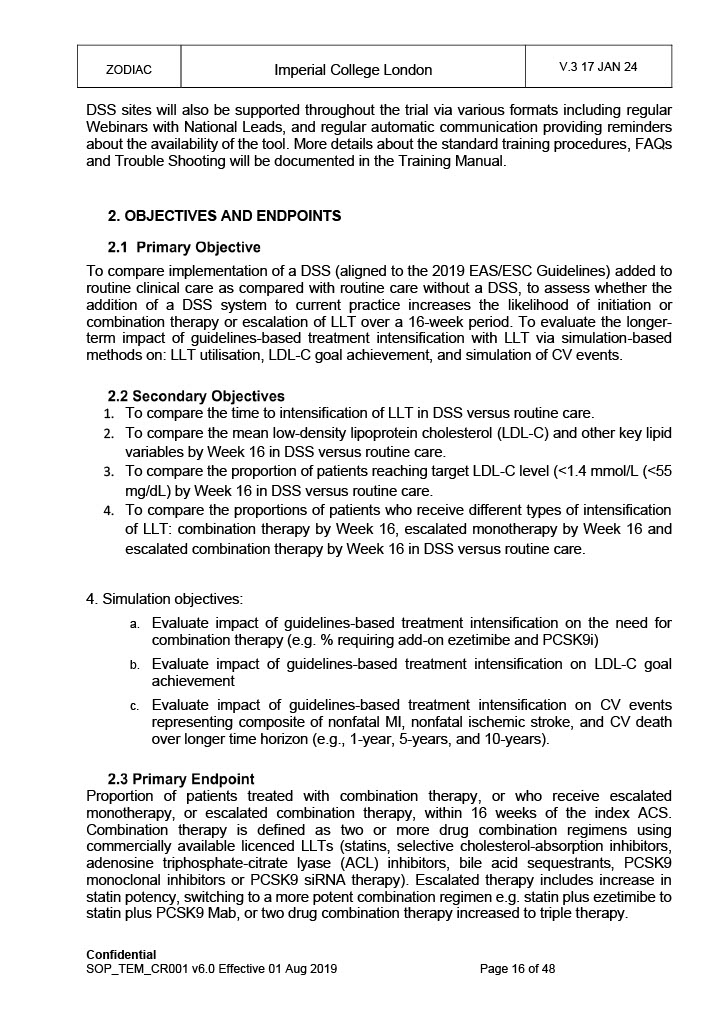

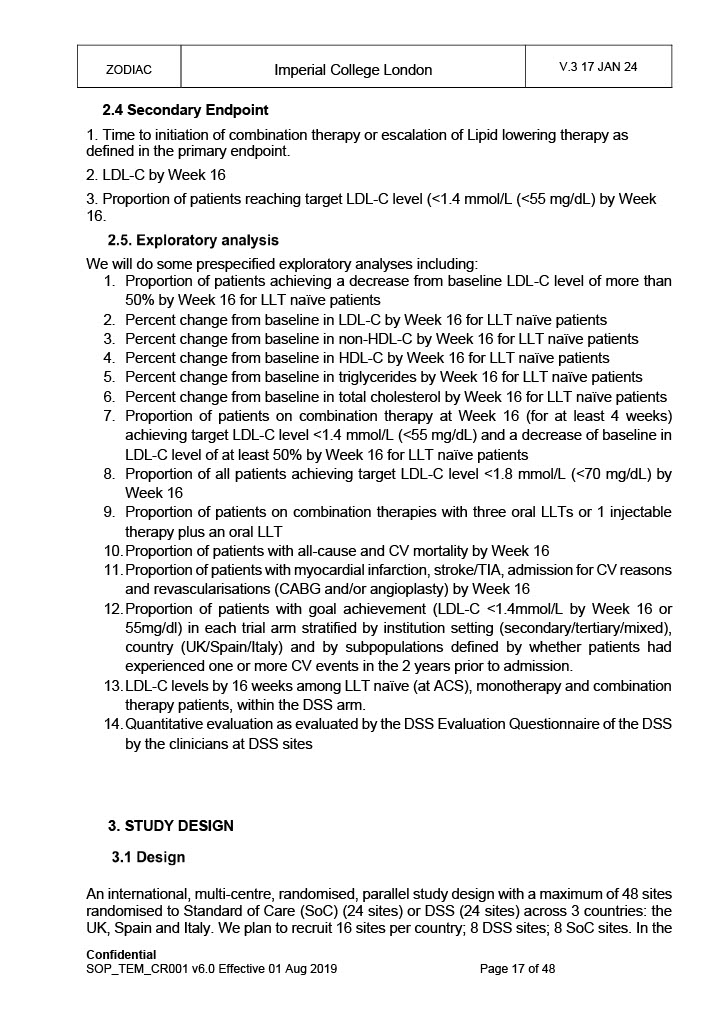

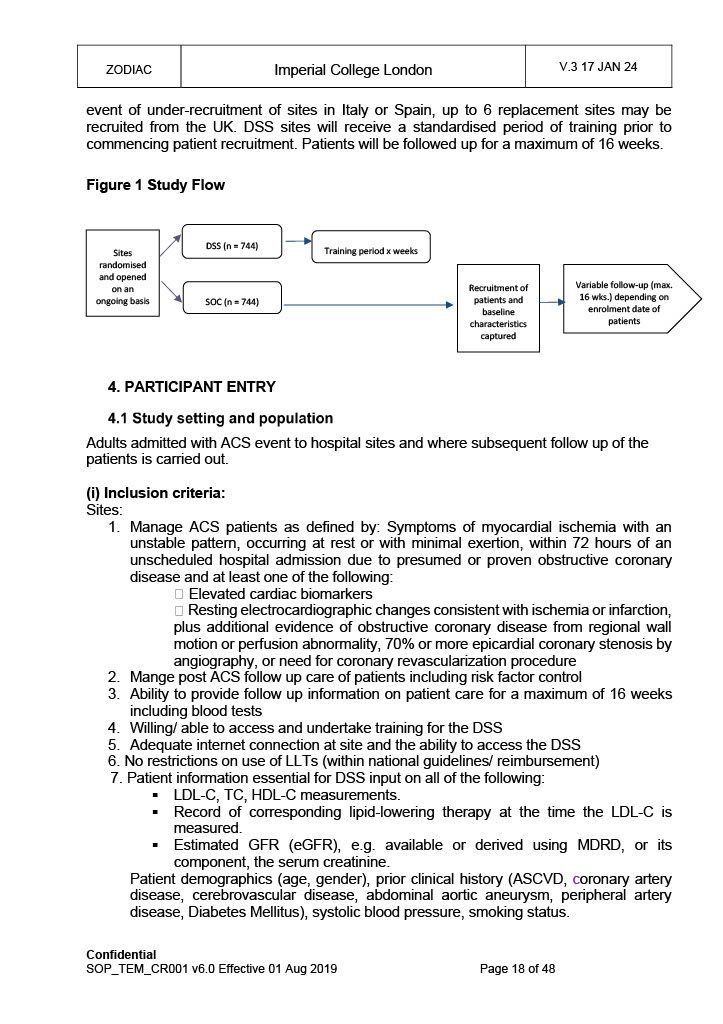

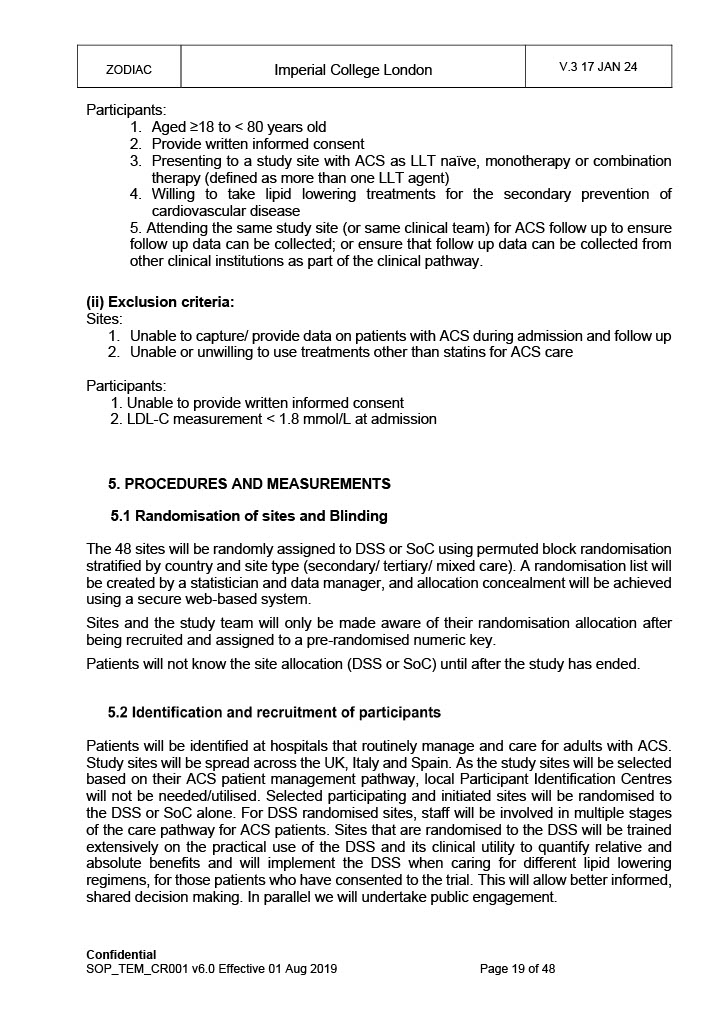

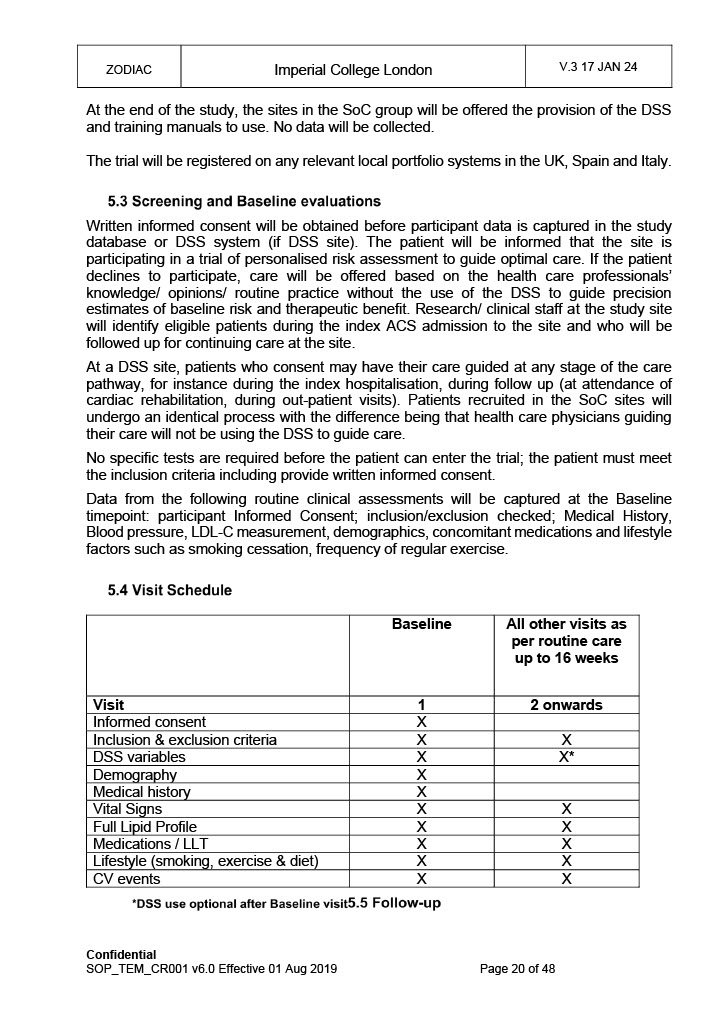

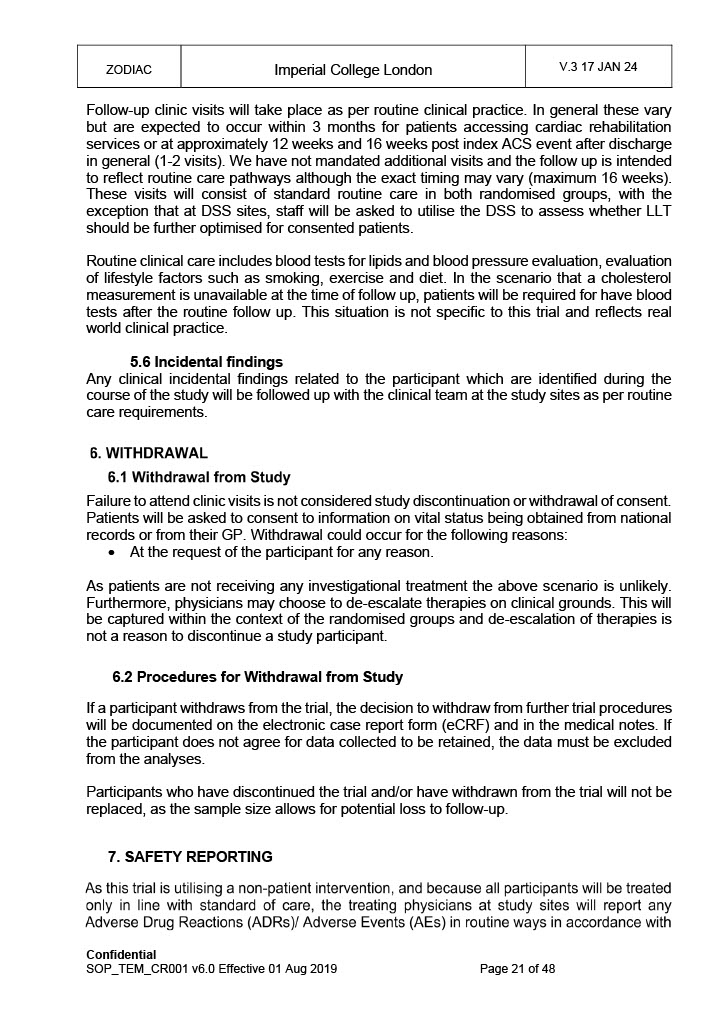

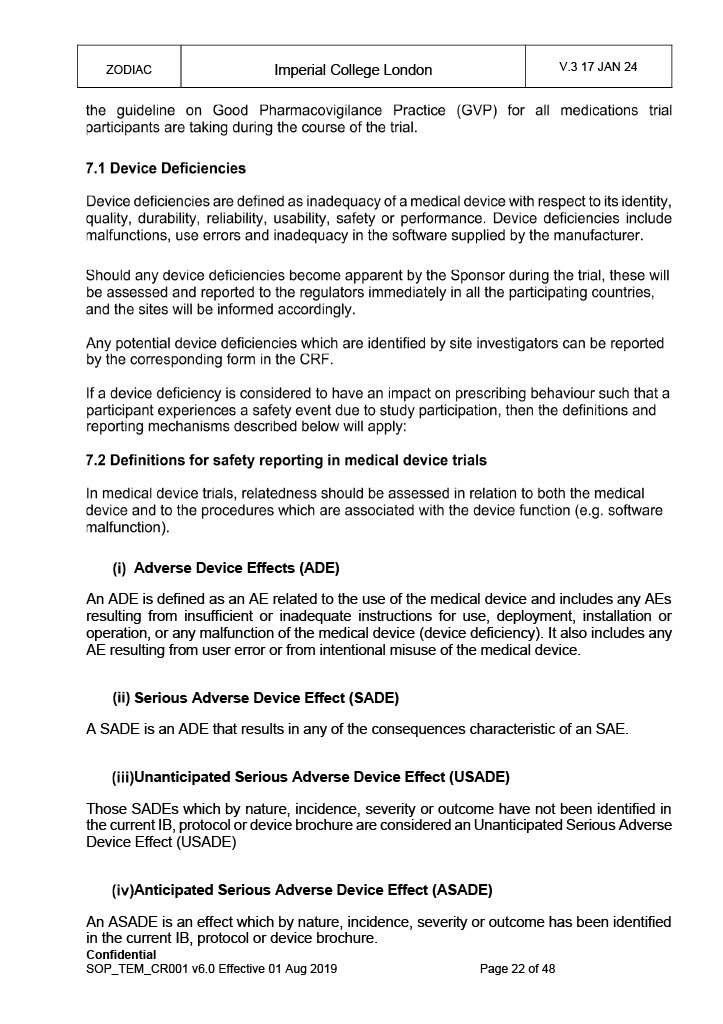

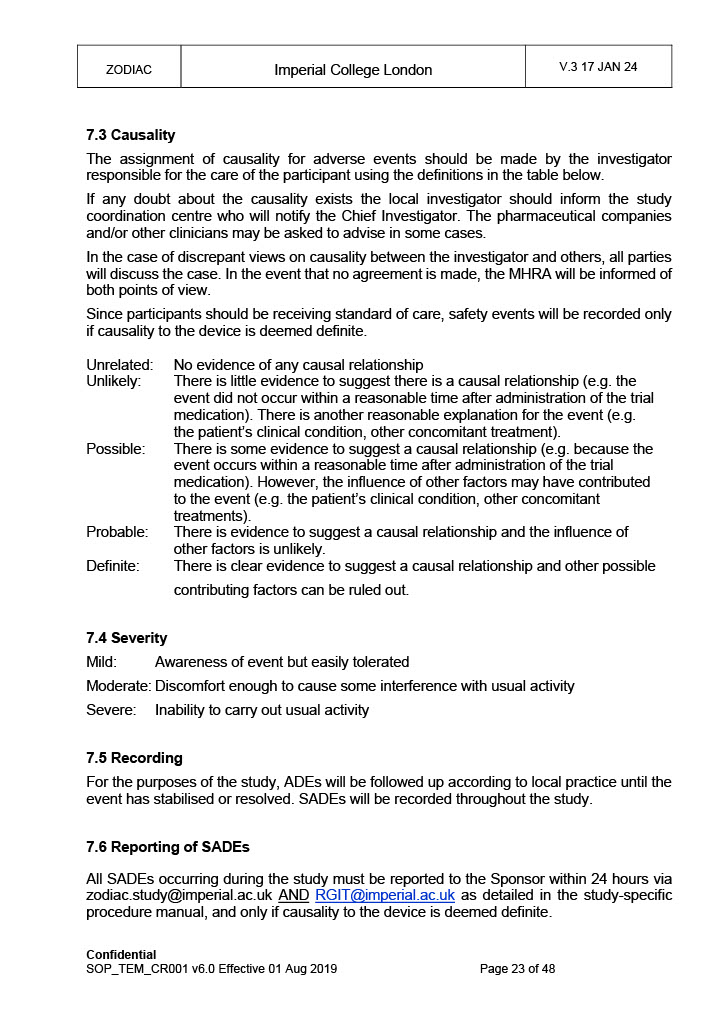

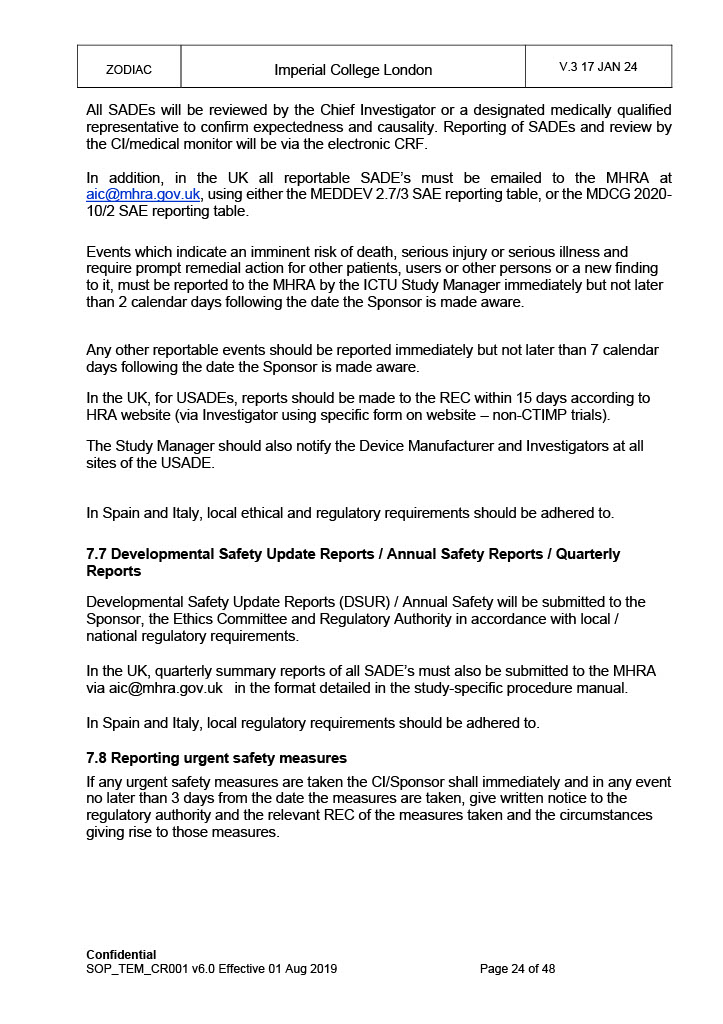

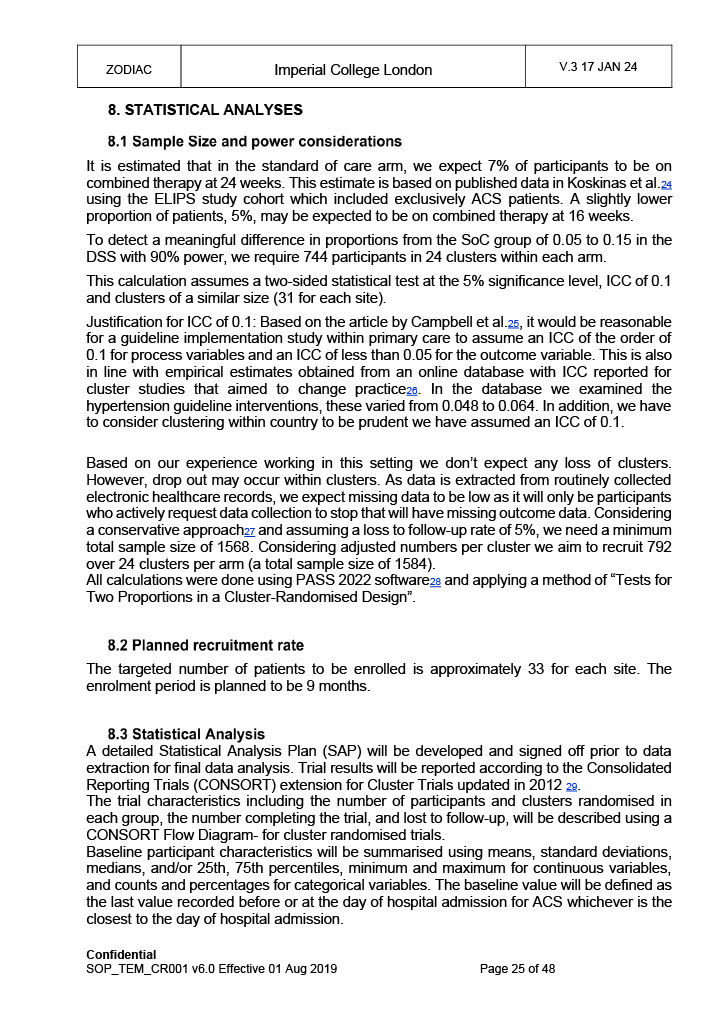

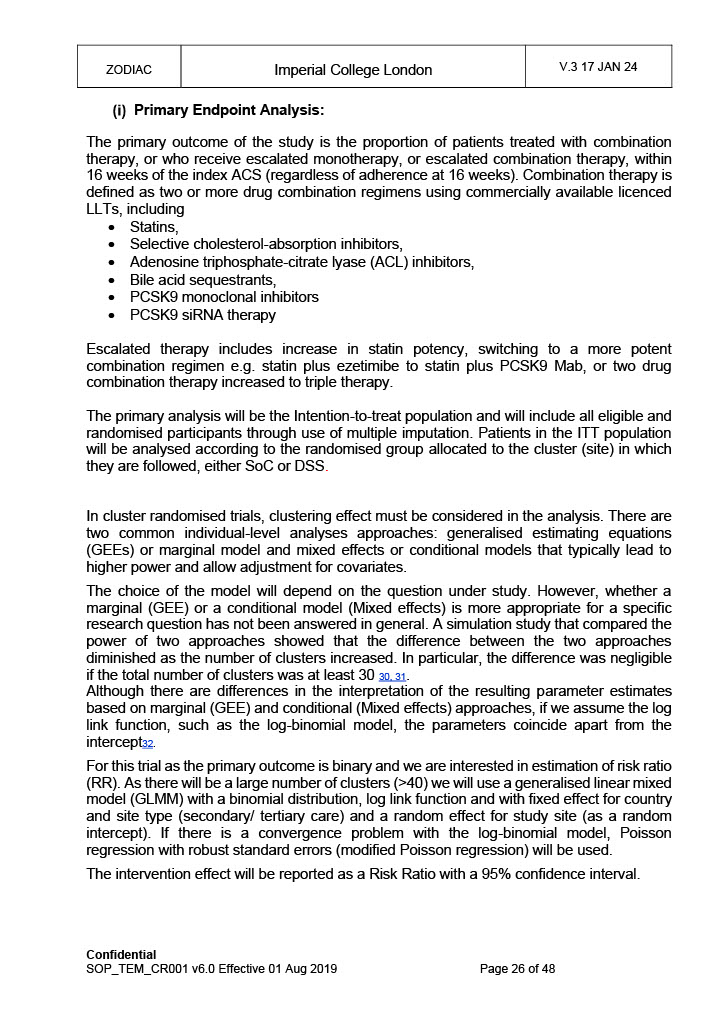

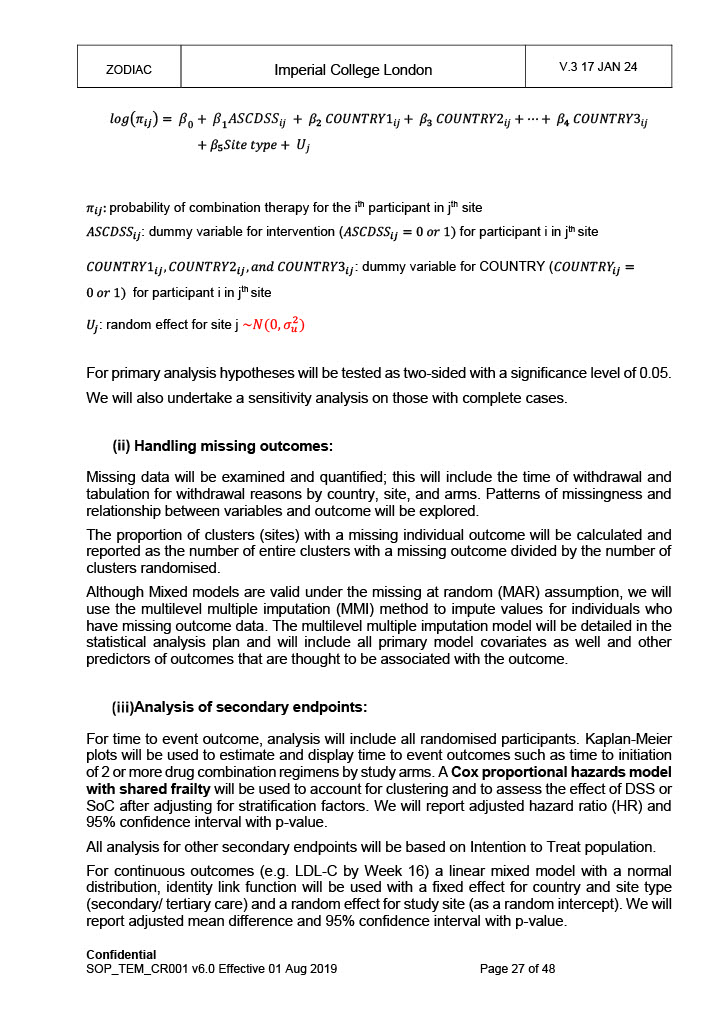

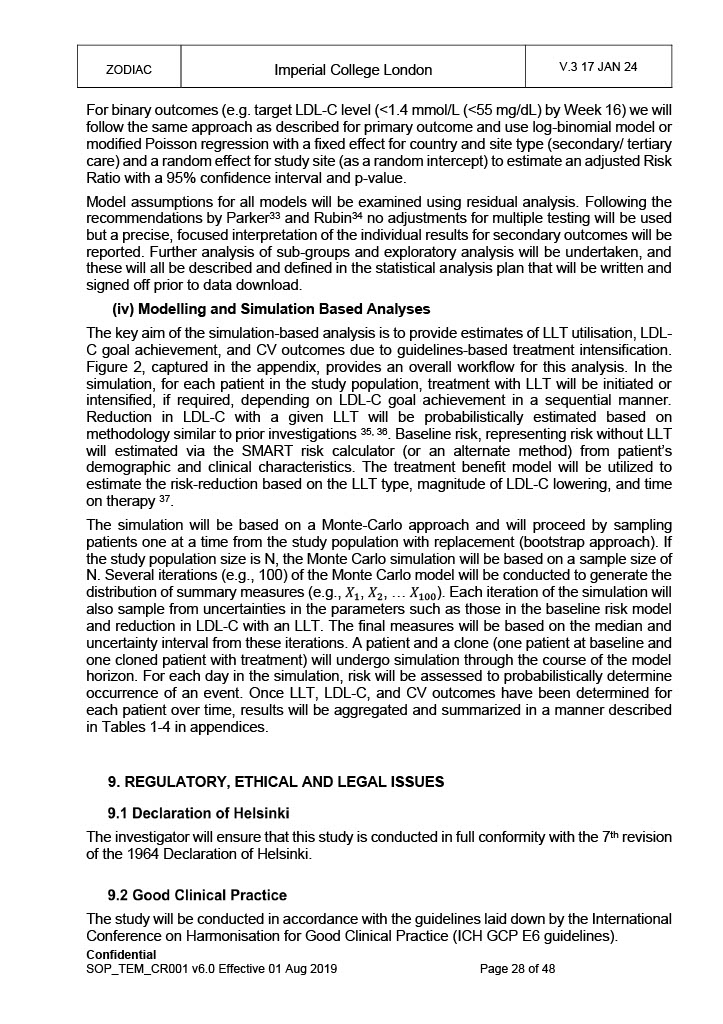

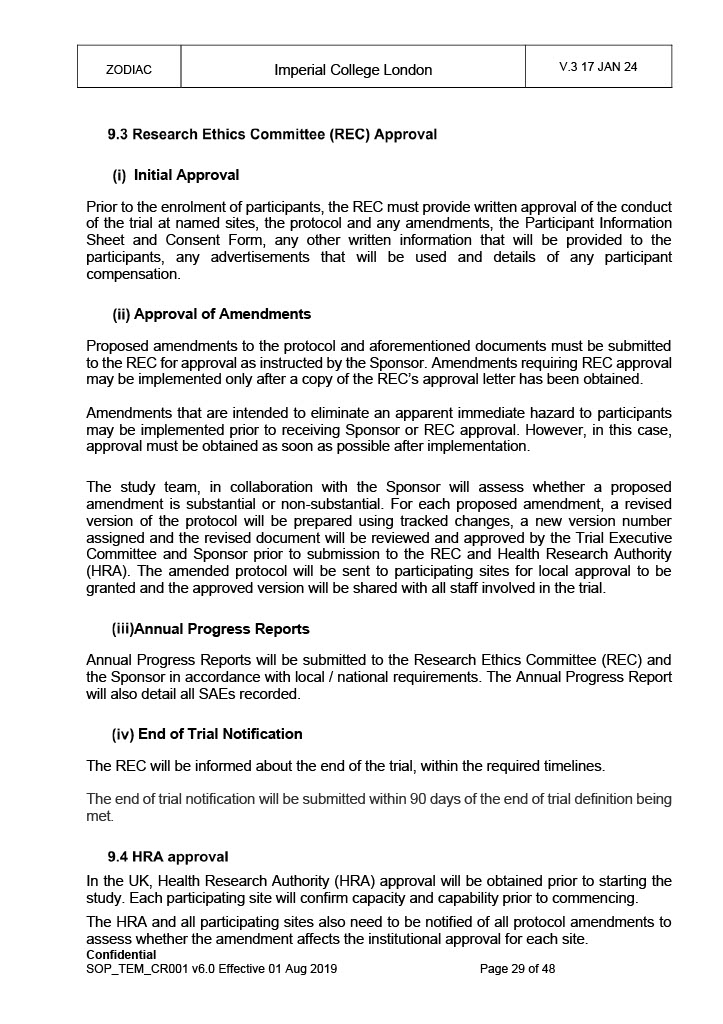

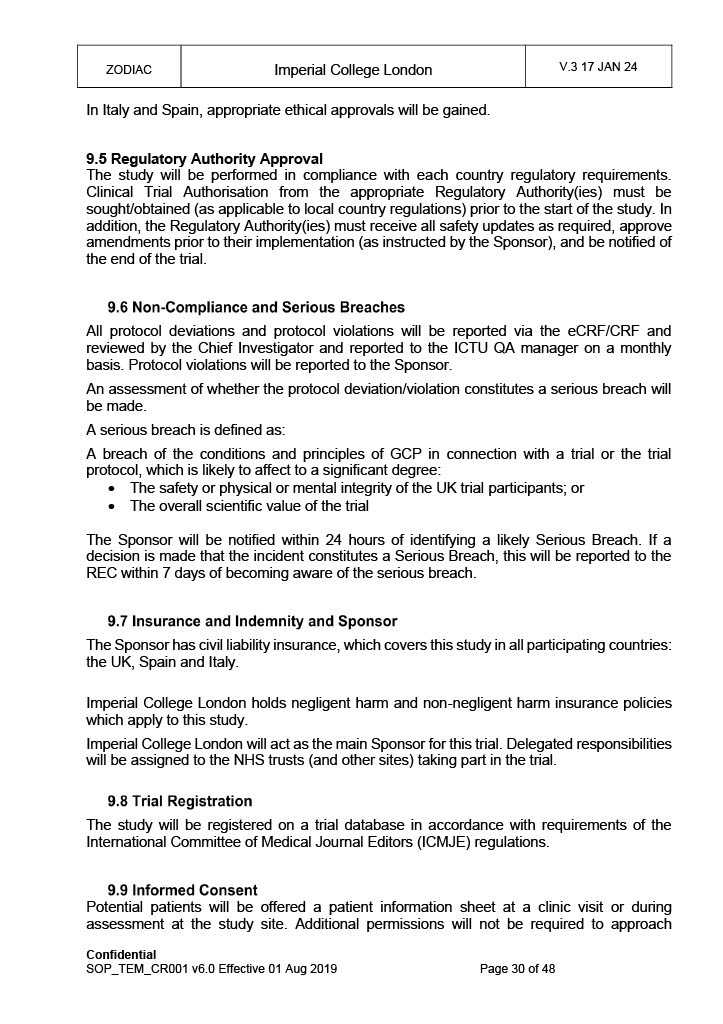


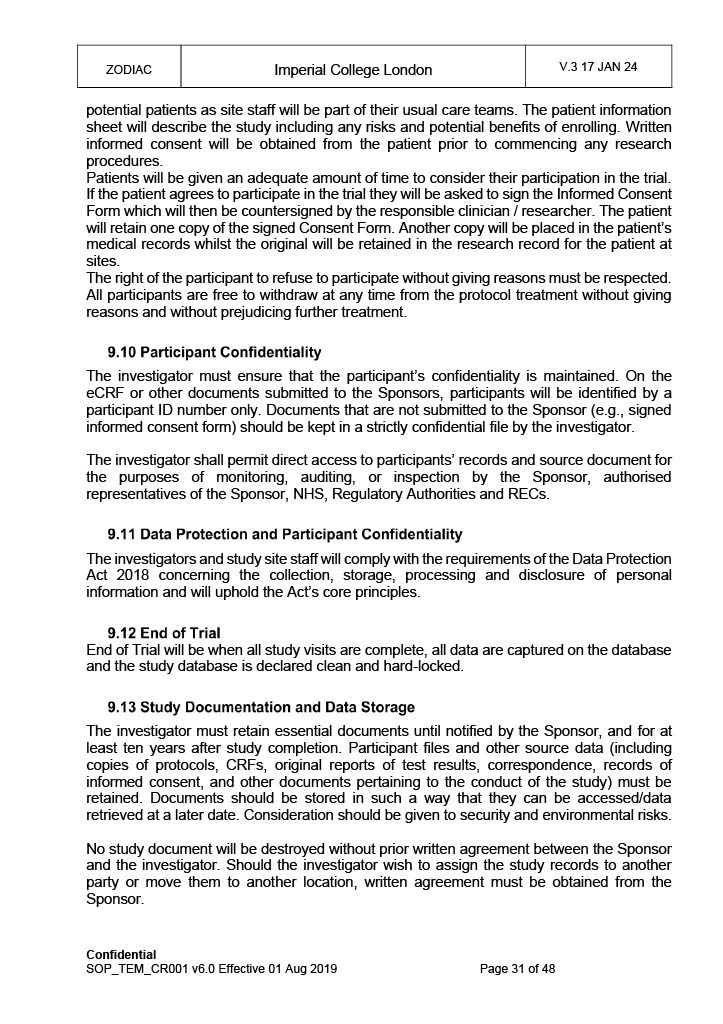

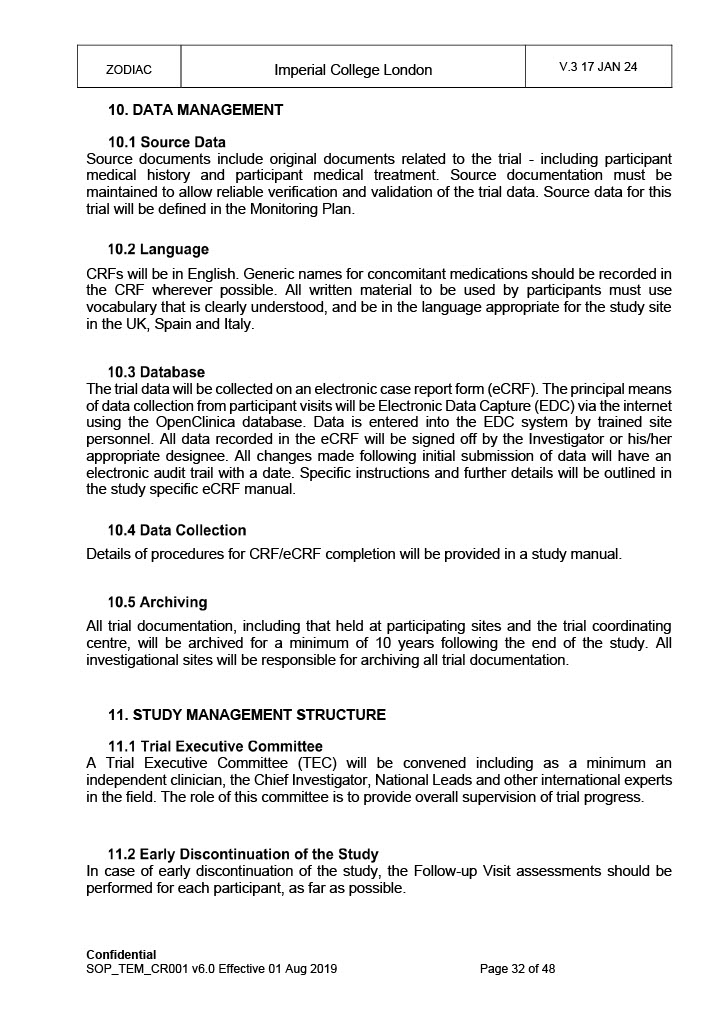

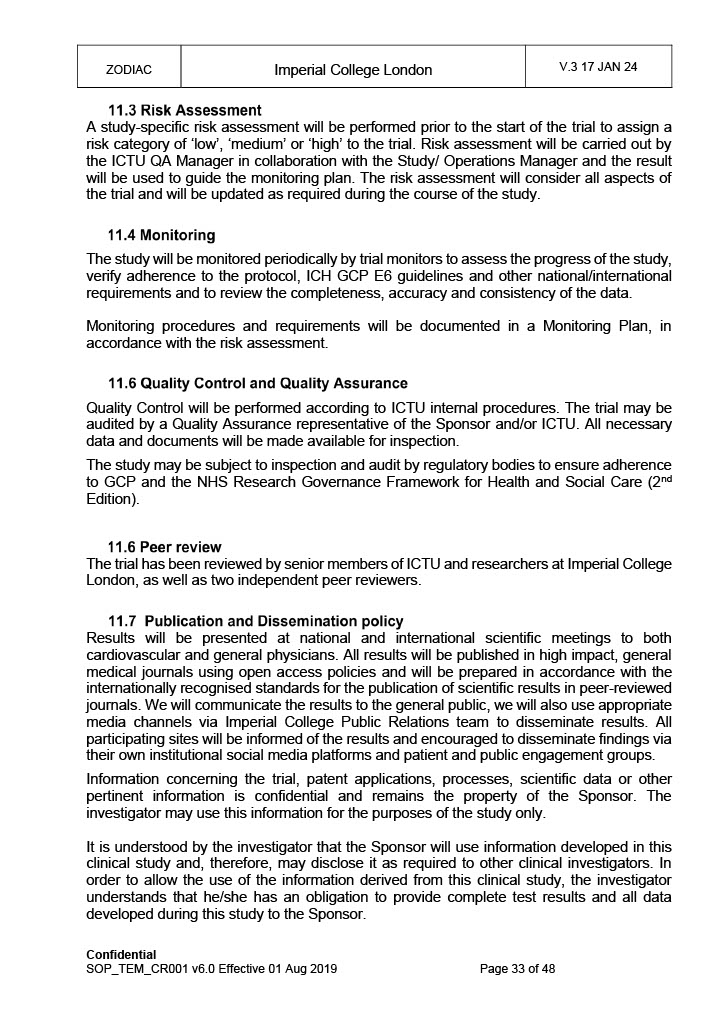

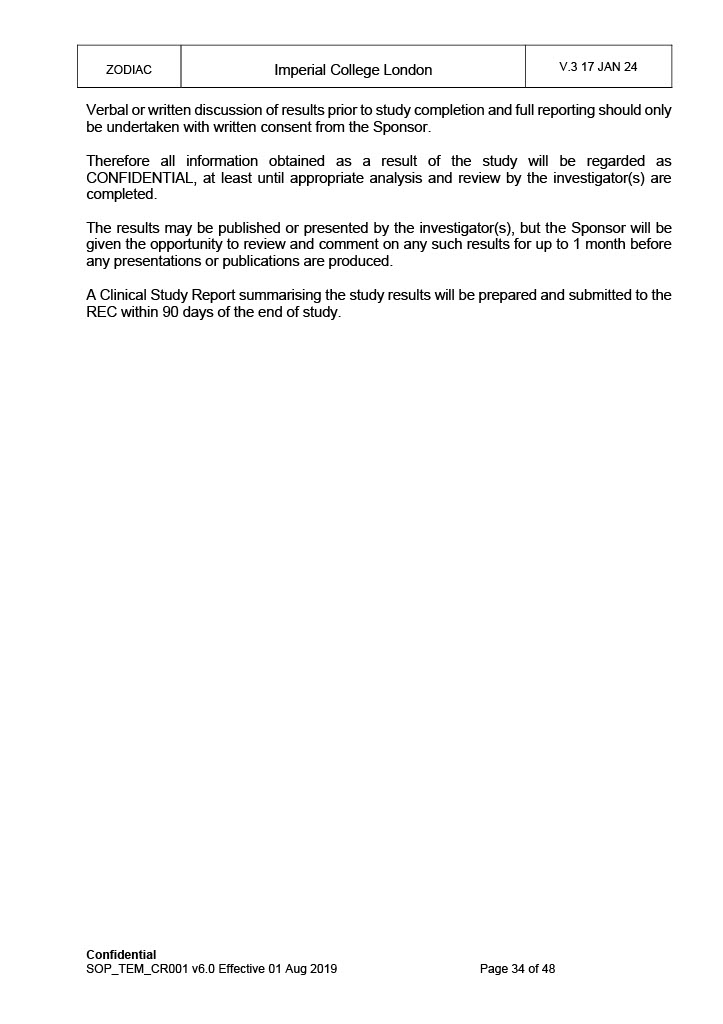

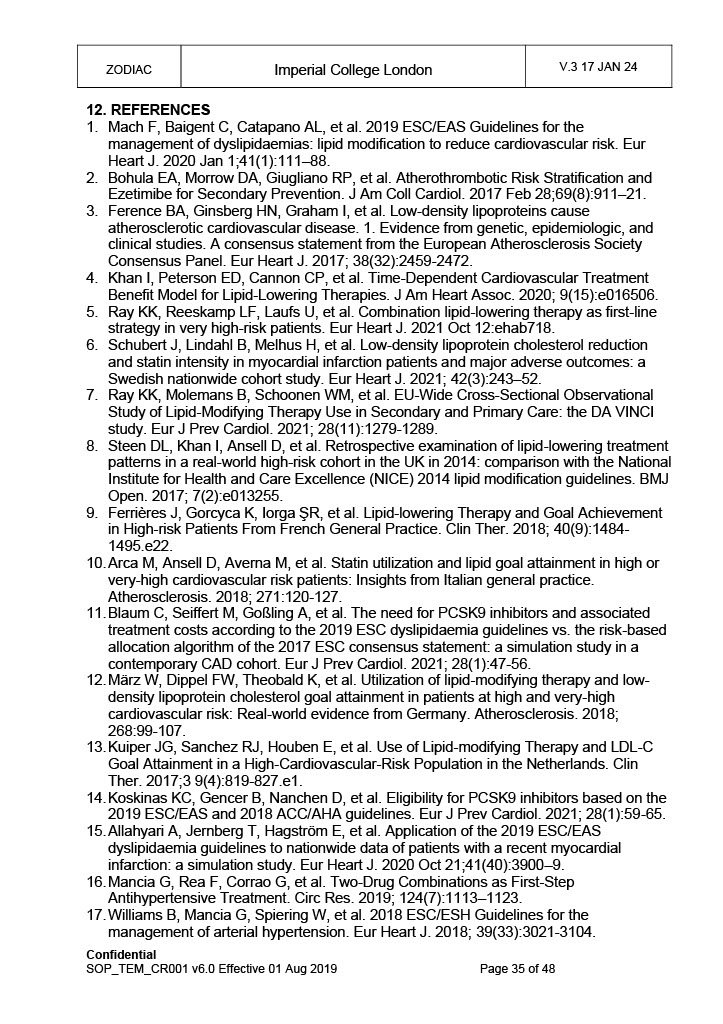

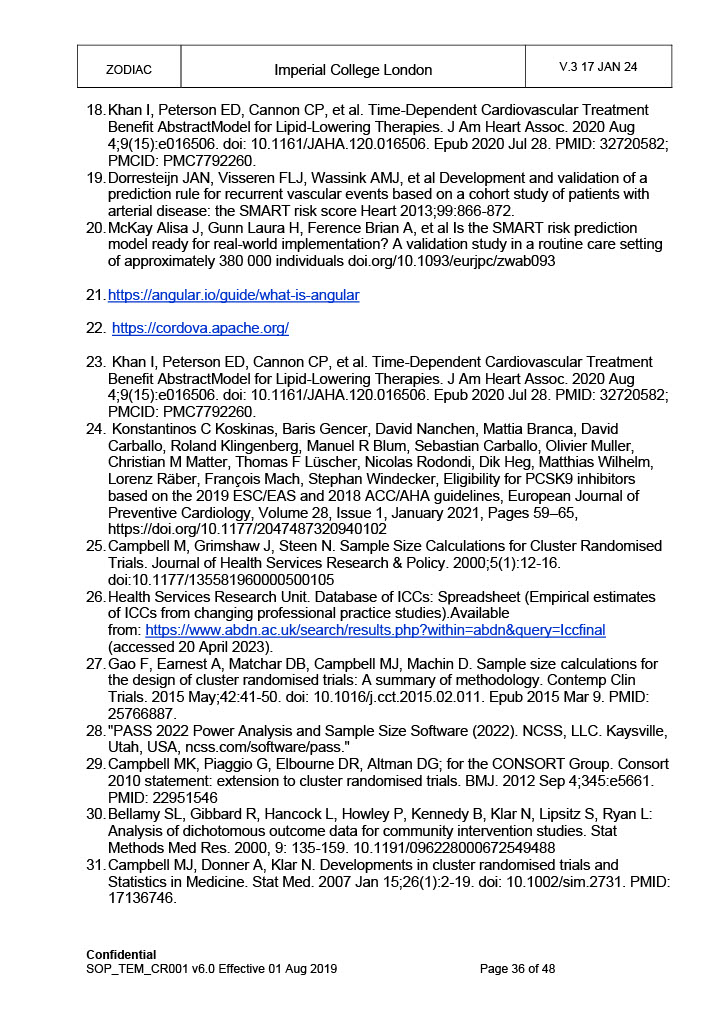

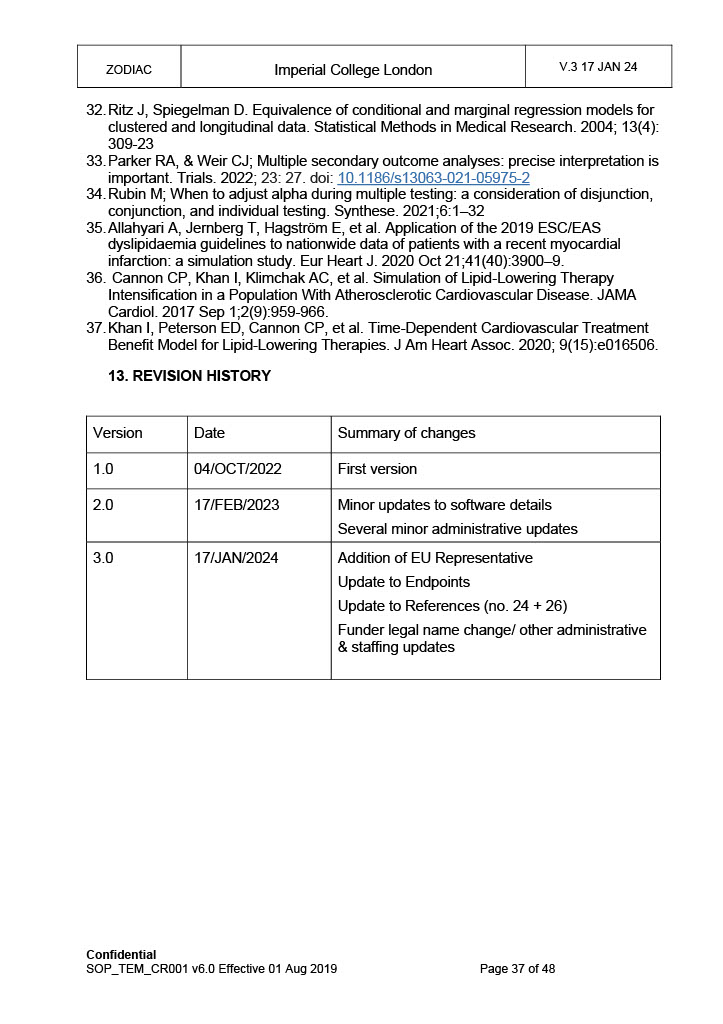

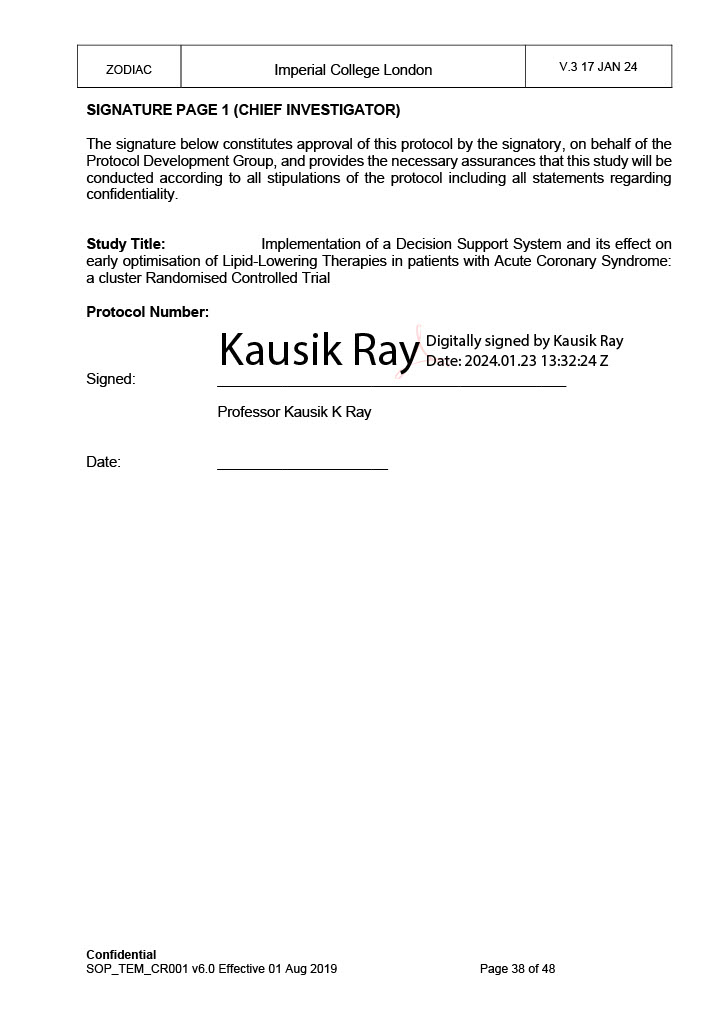

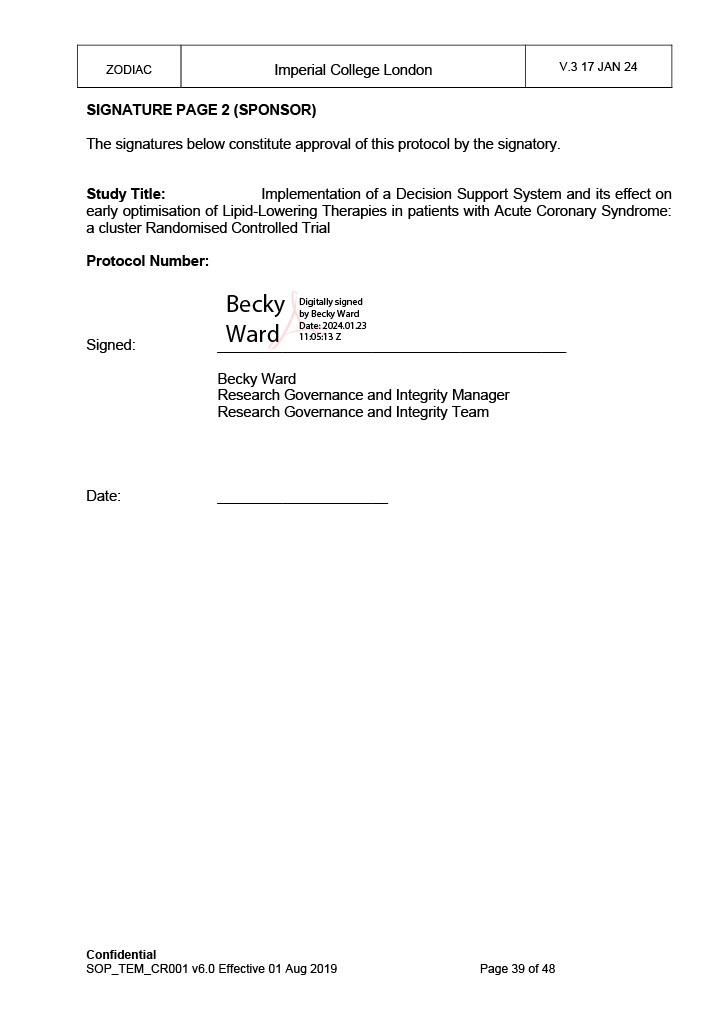

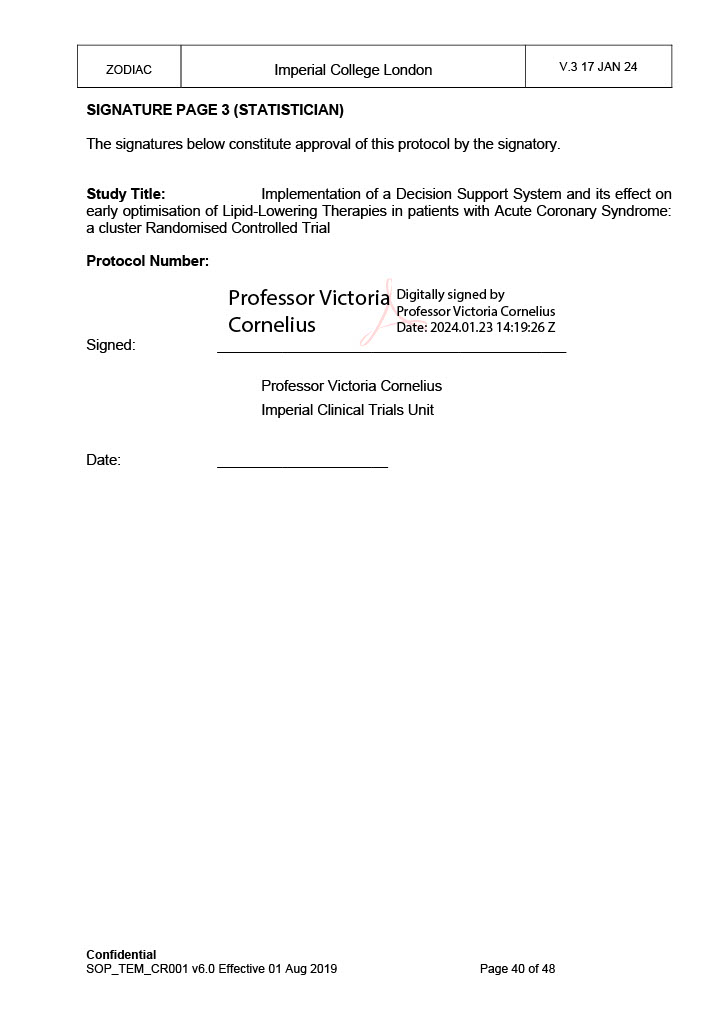

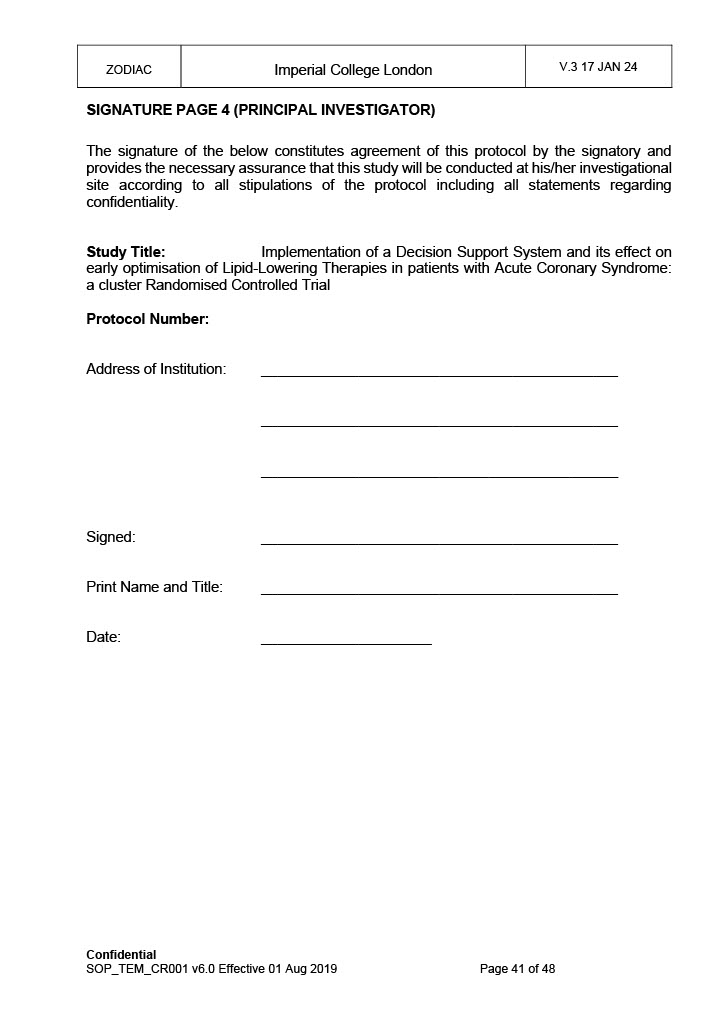

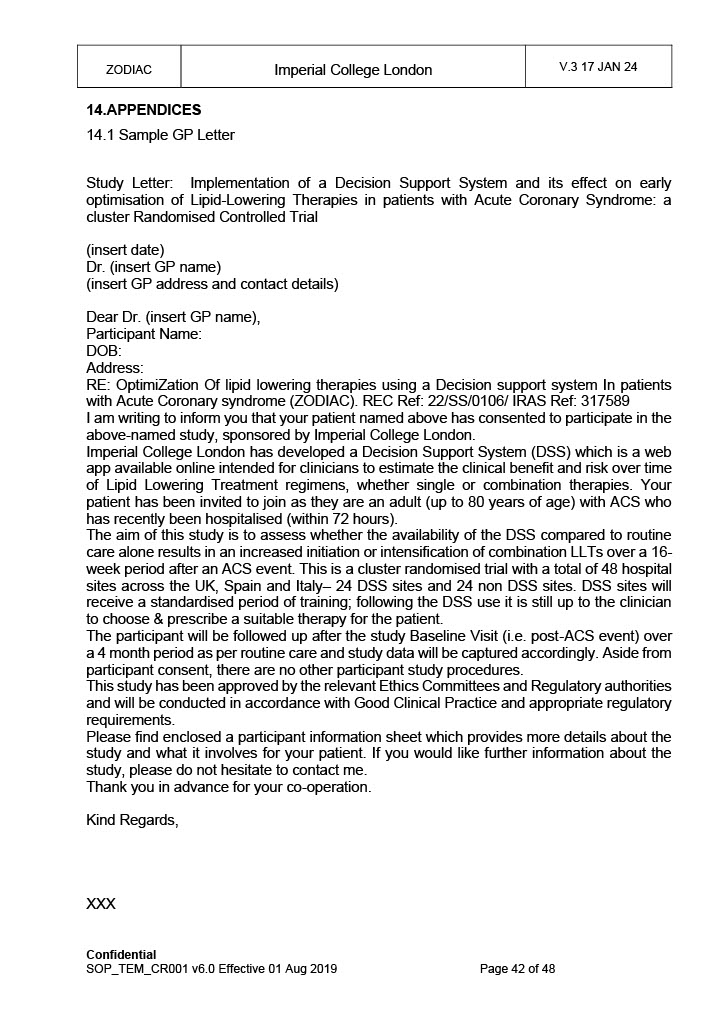

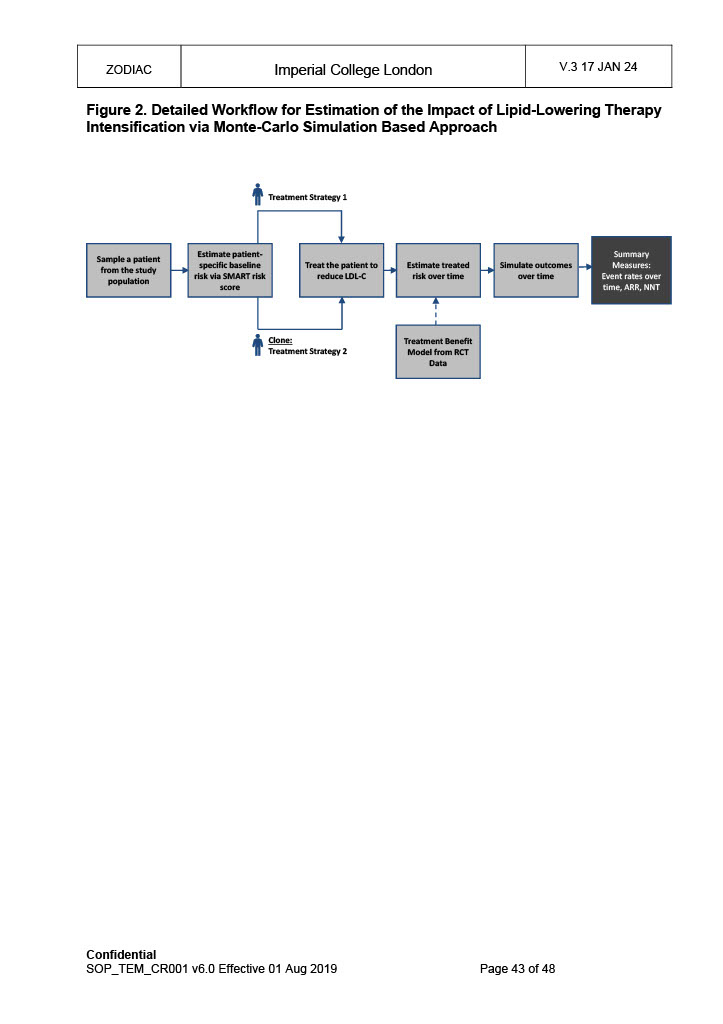

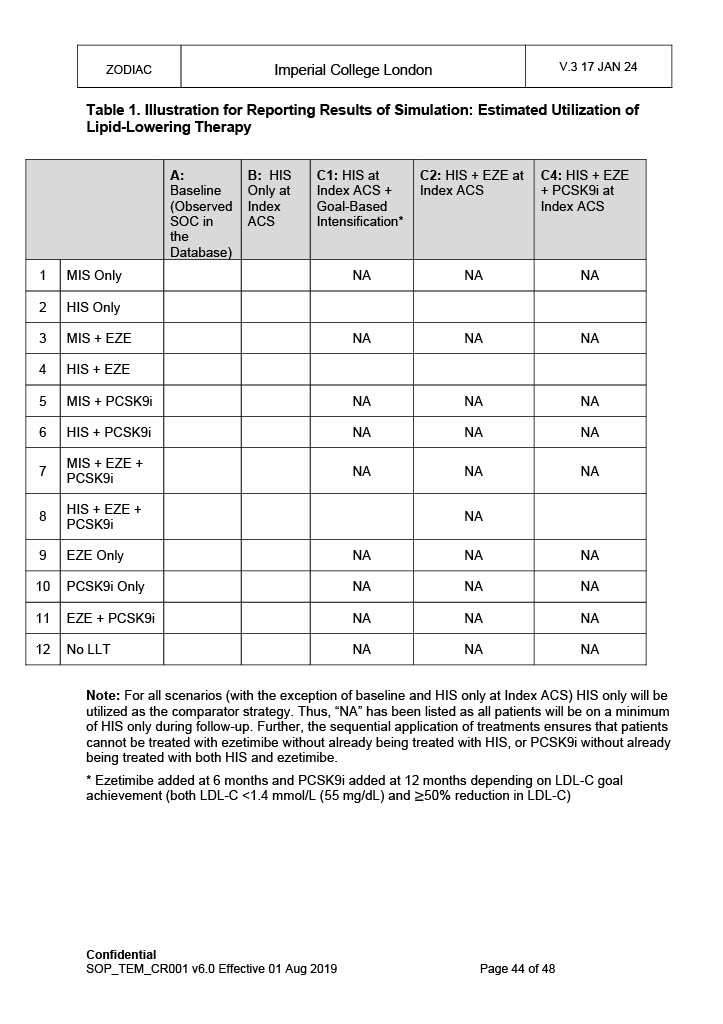

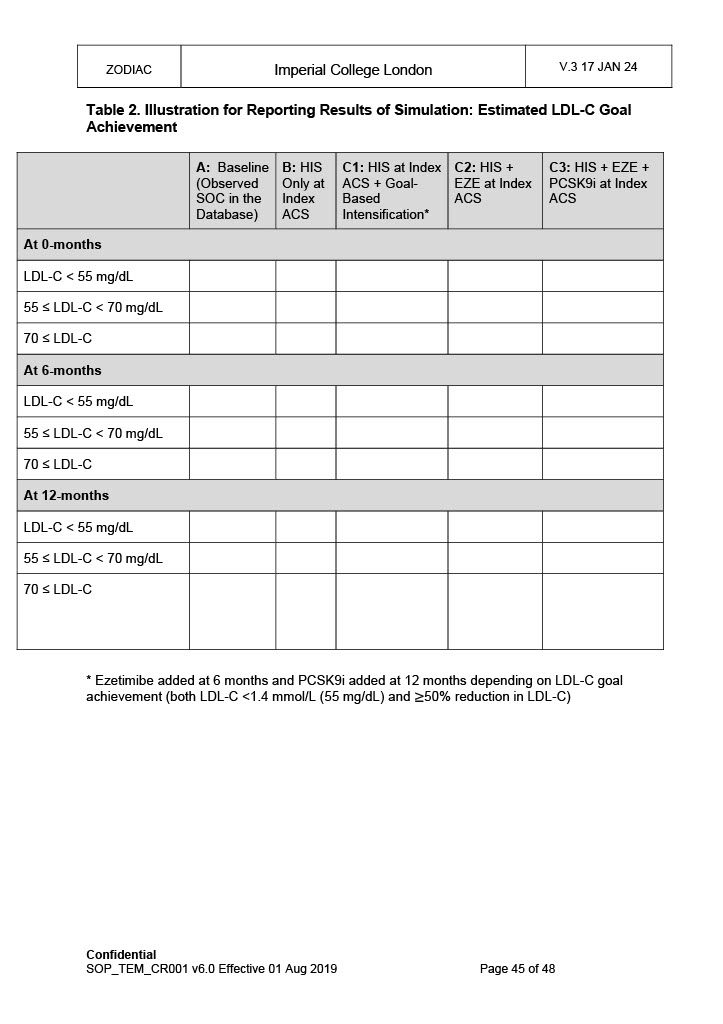

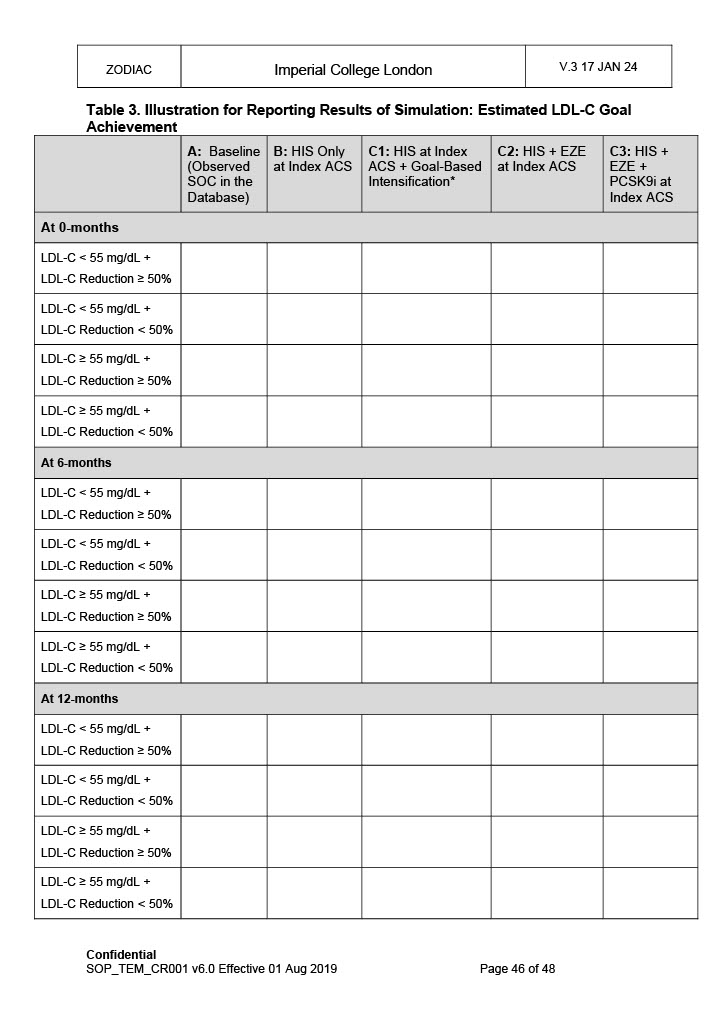


## Supplementary Document 2: Statistical Analysis Plan (SAP)


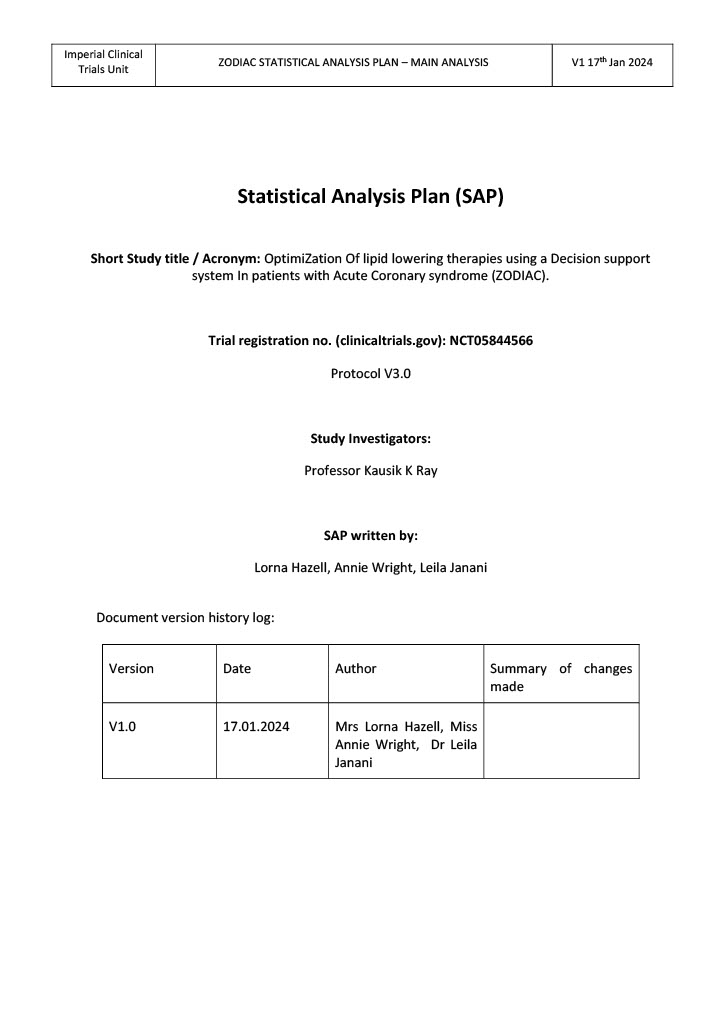

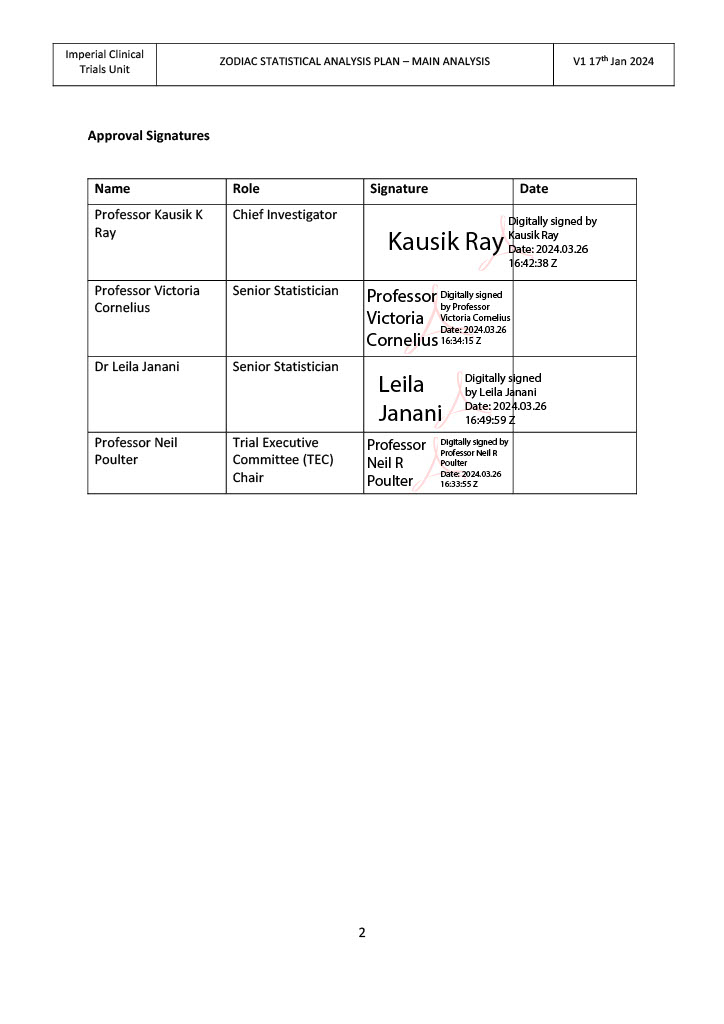

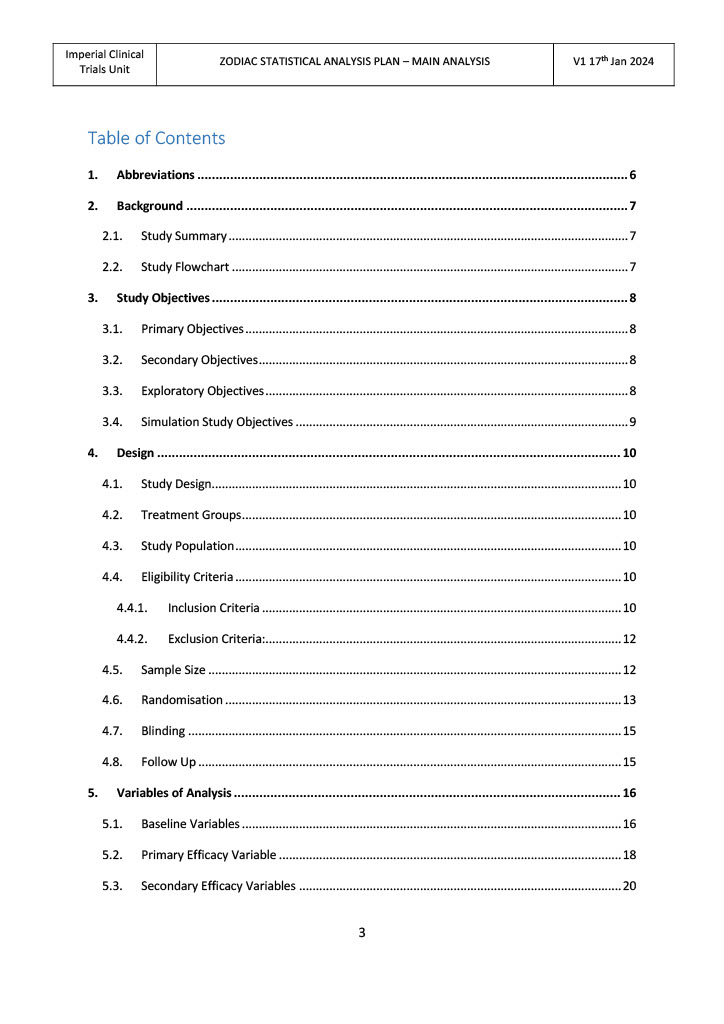

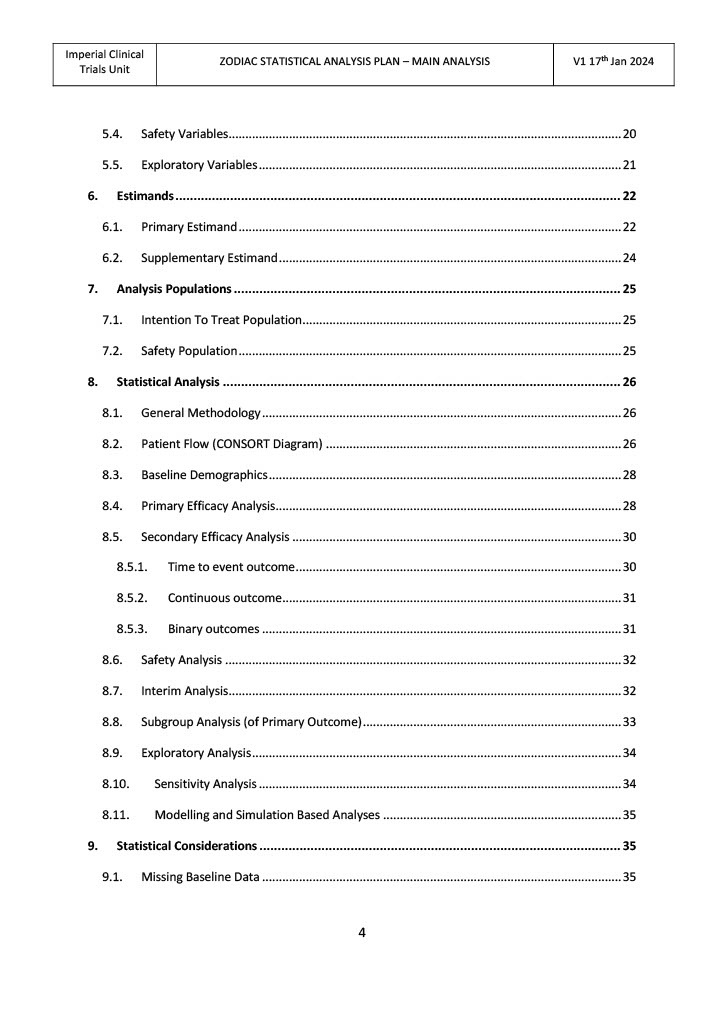

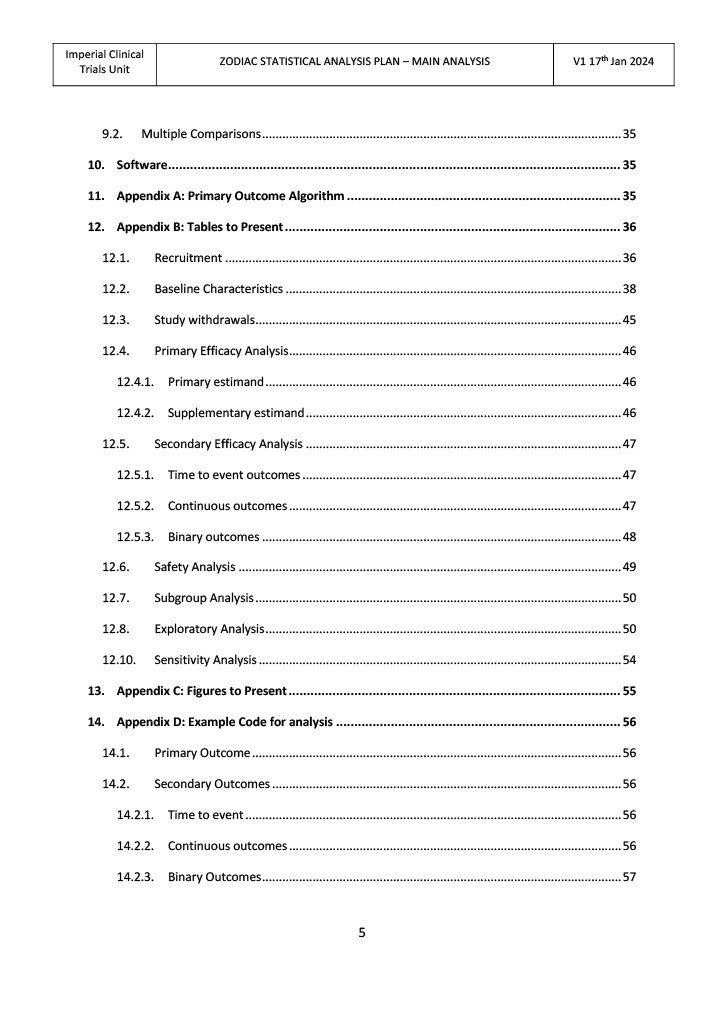

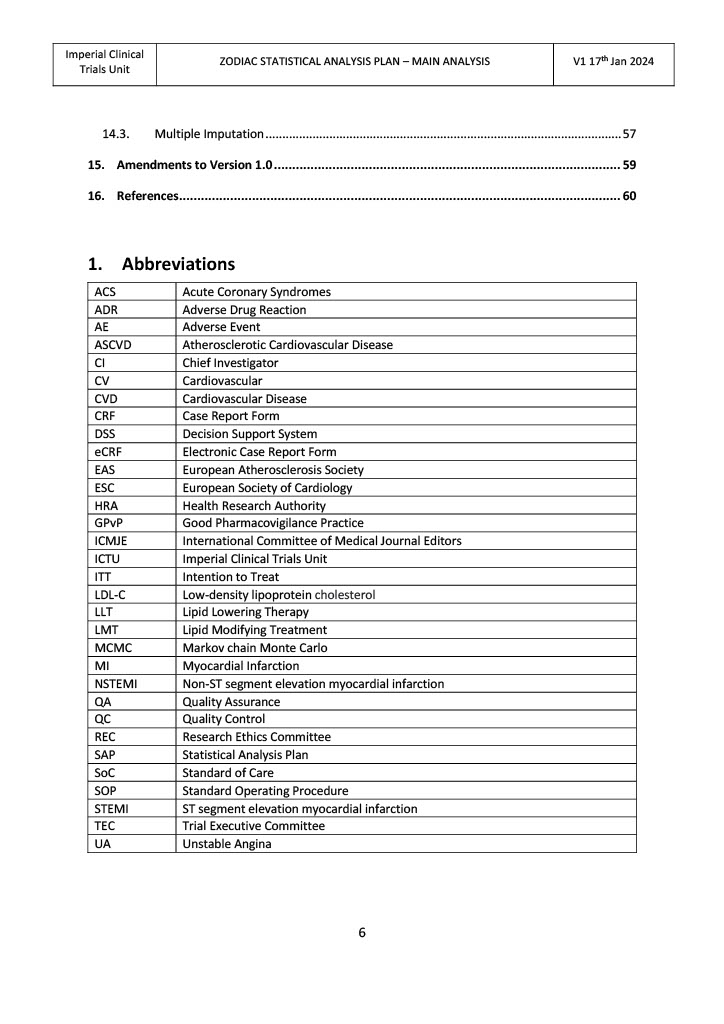

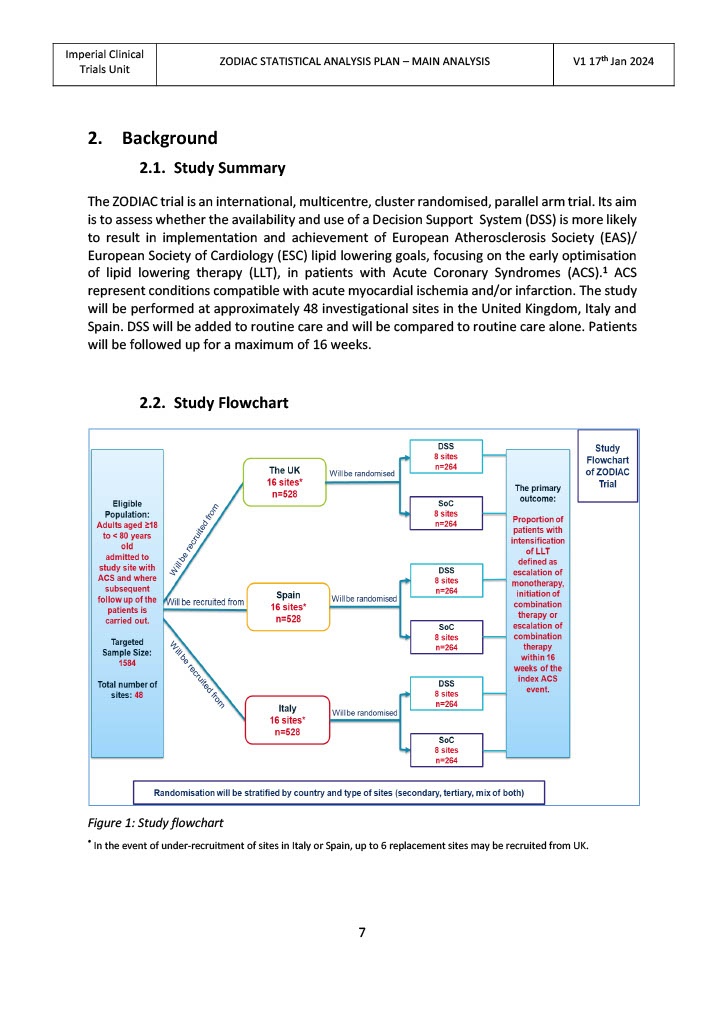

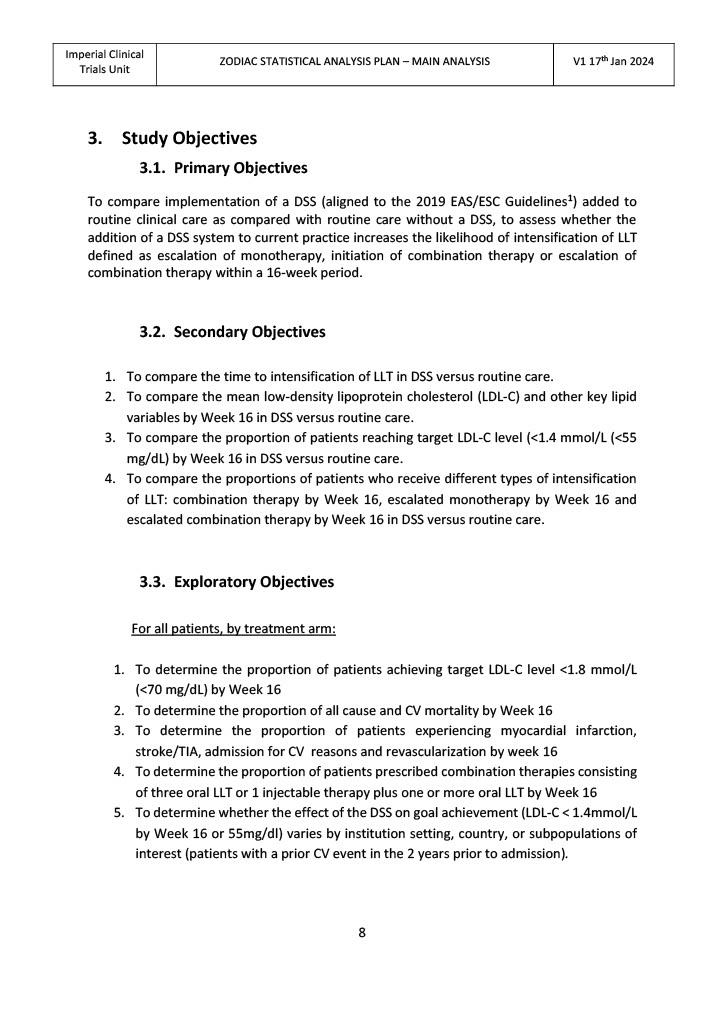

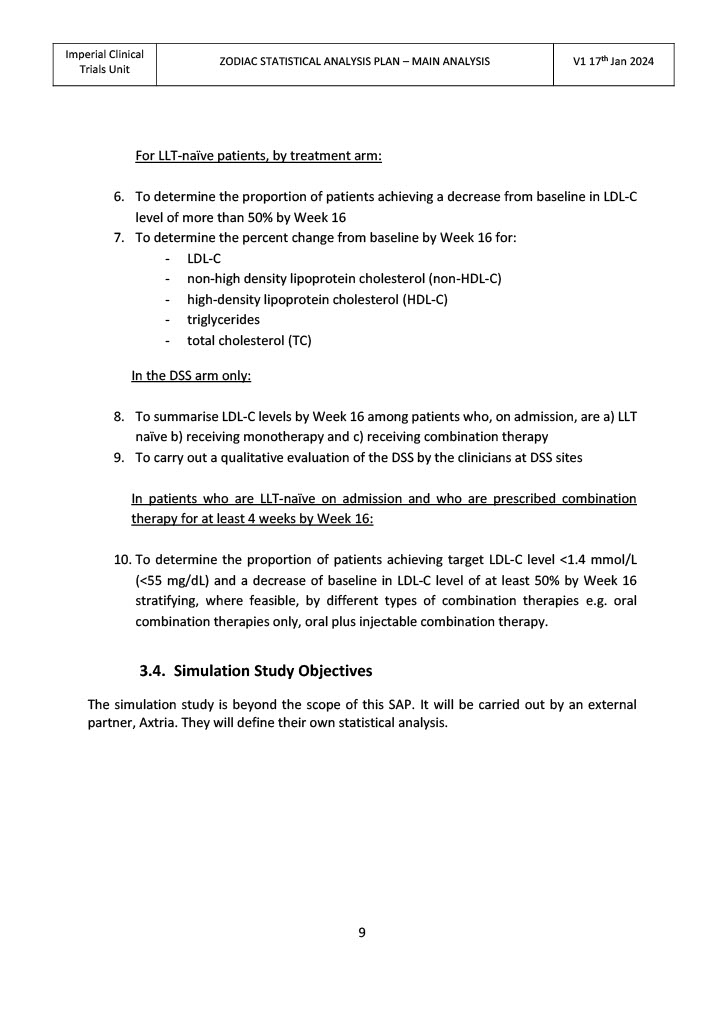

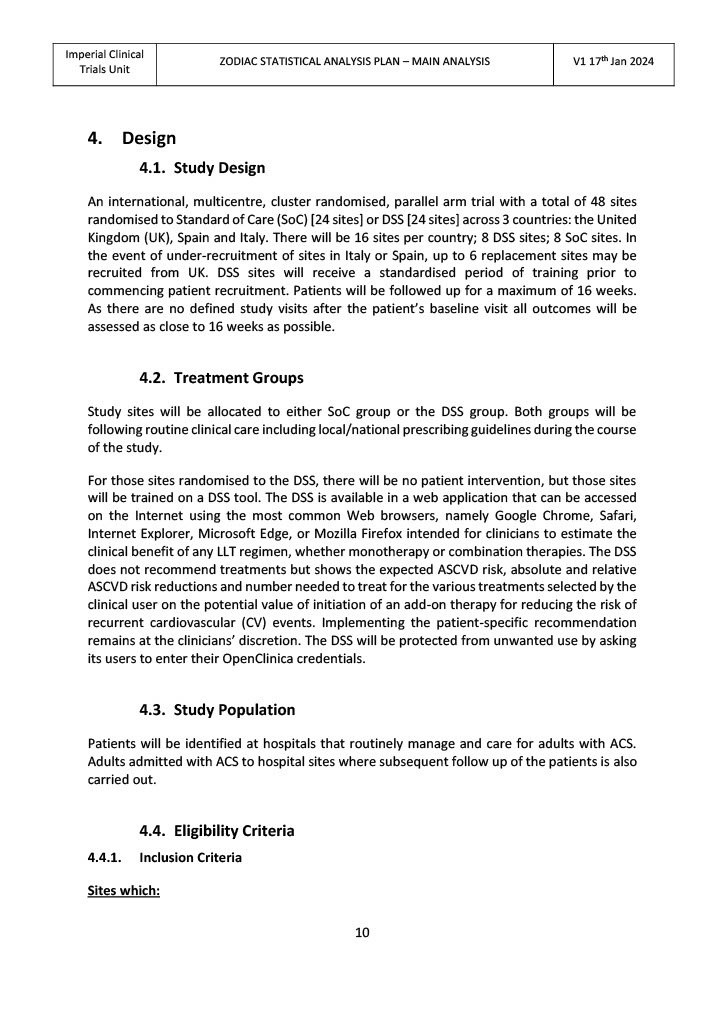

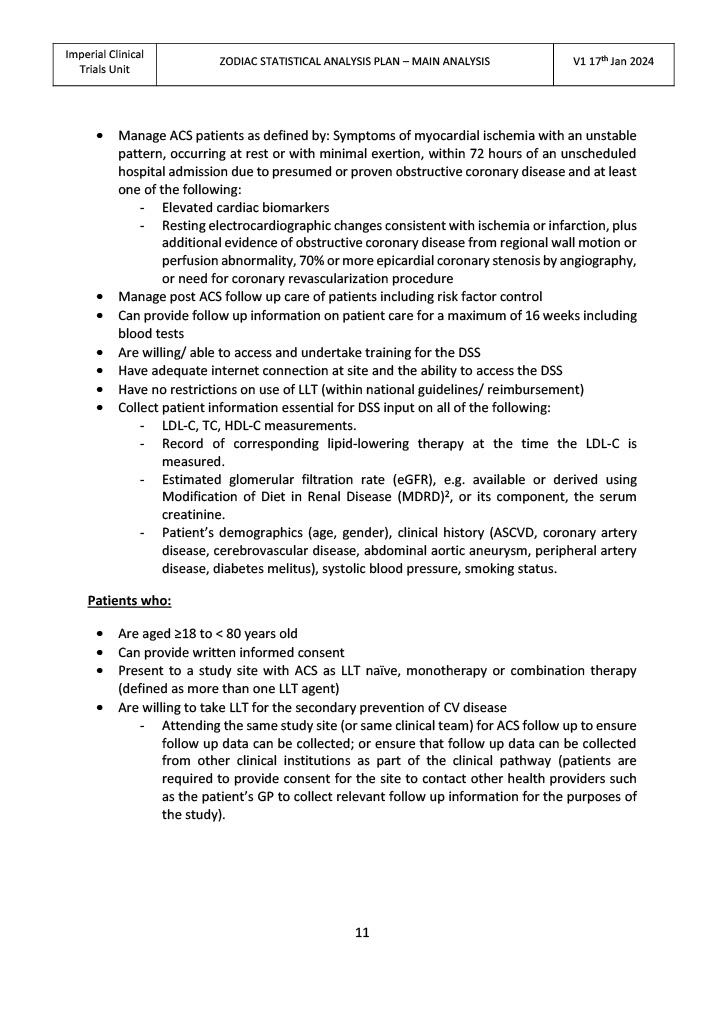

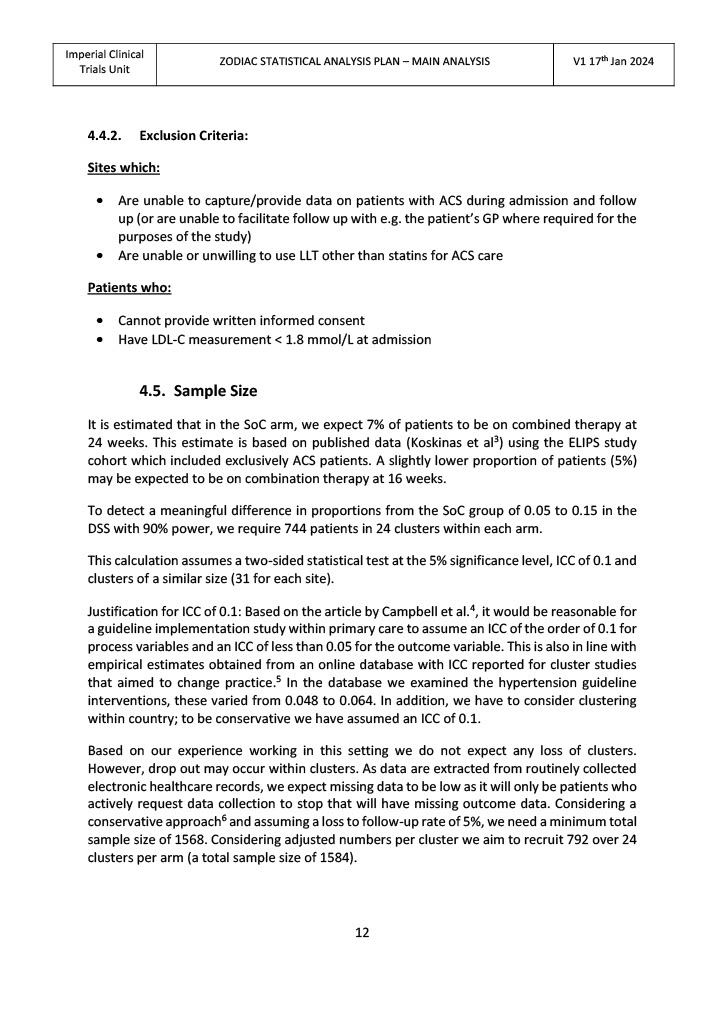

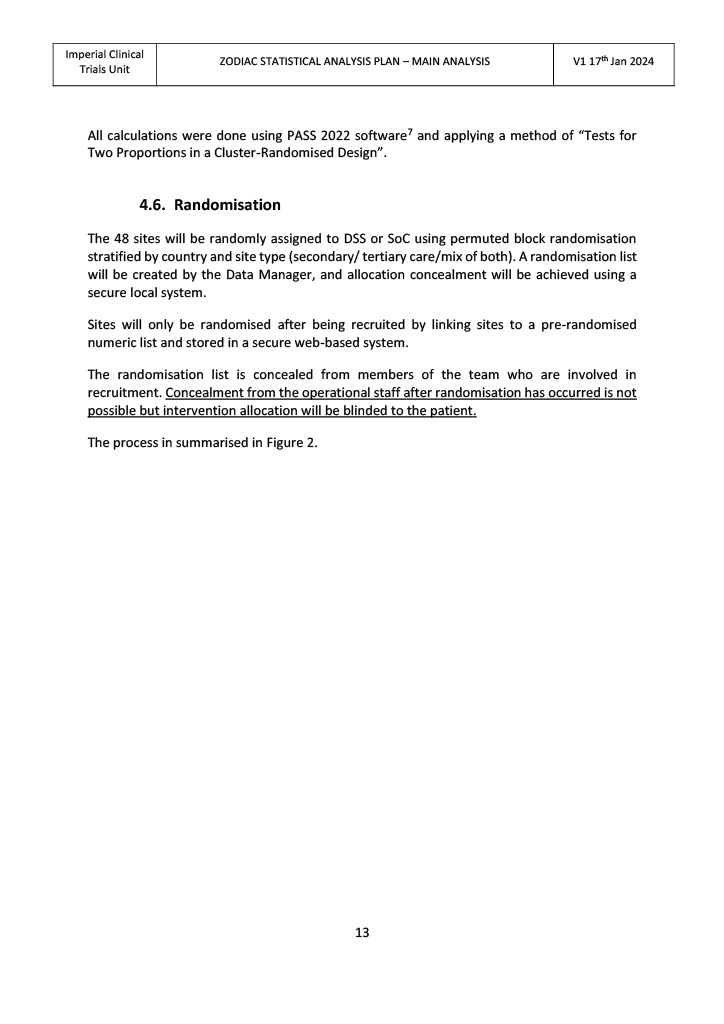

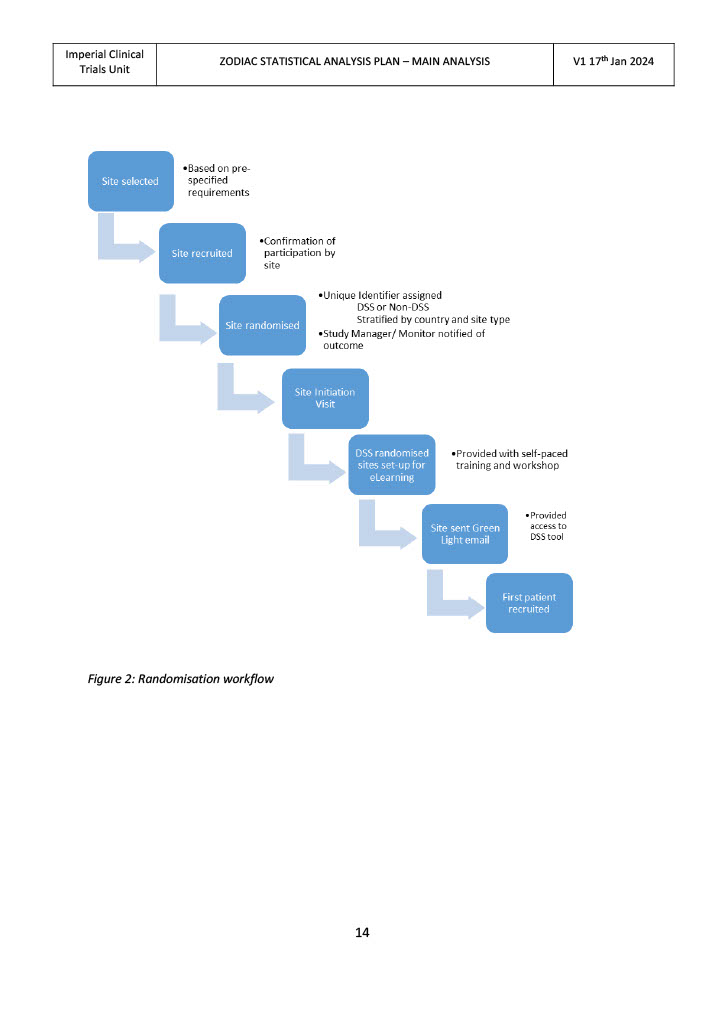

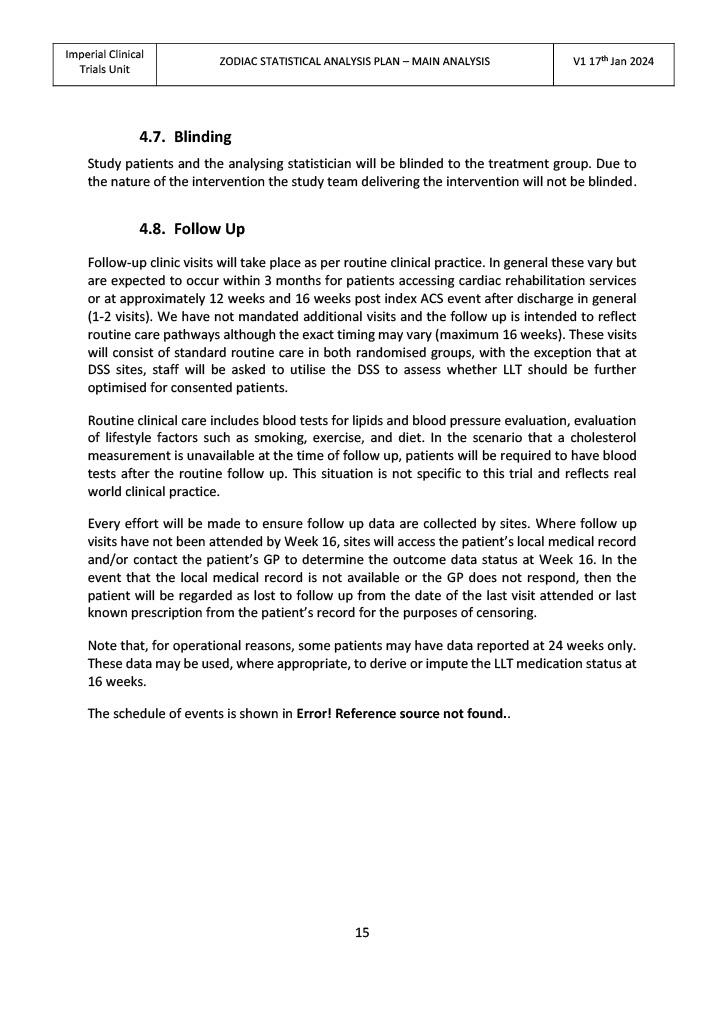

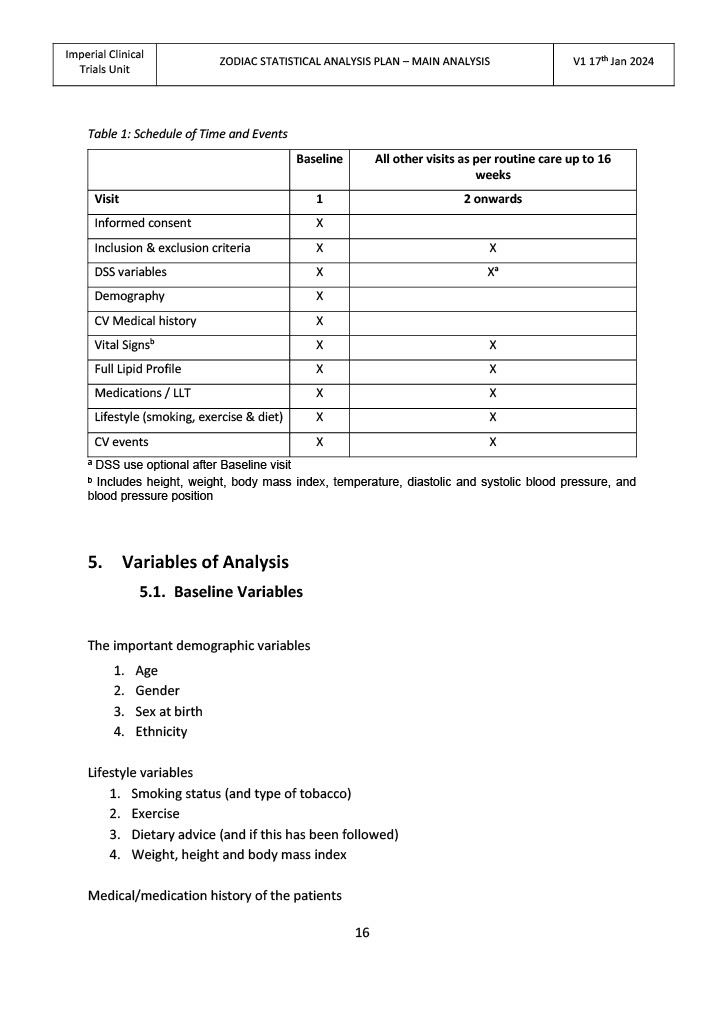

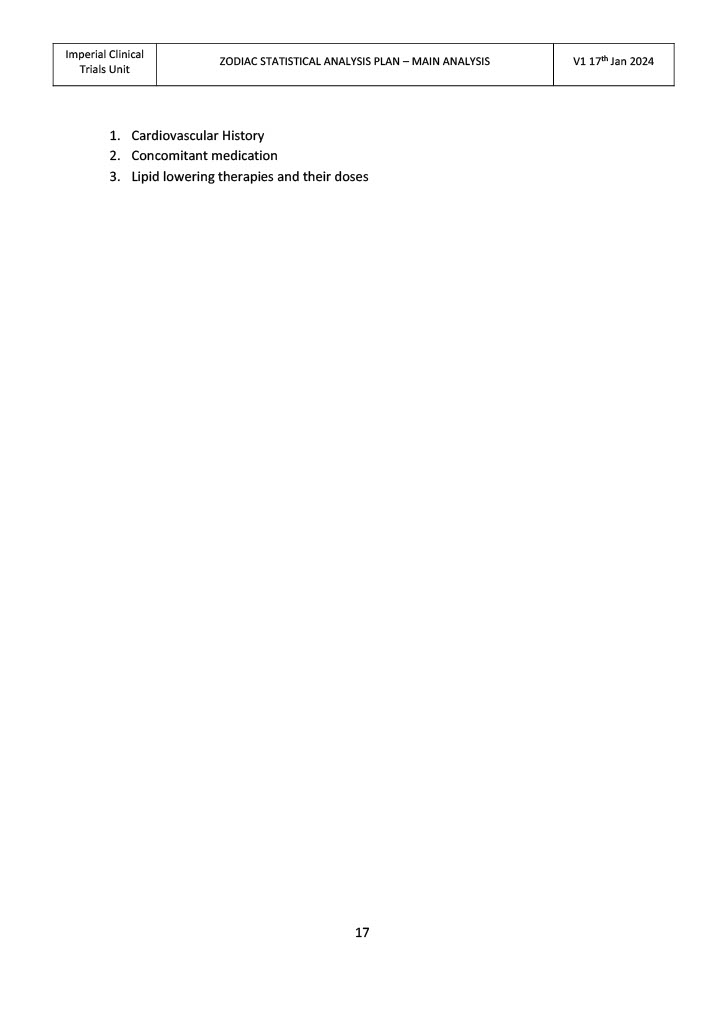

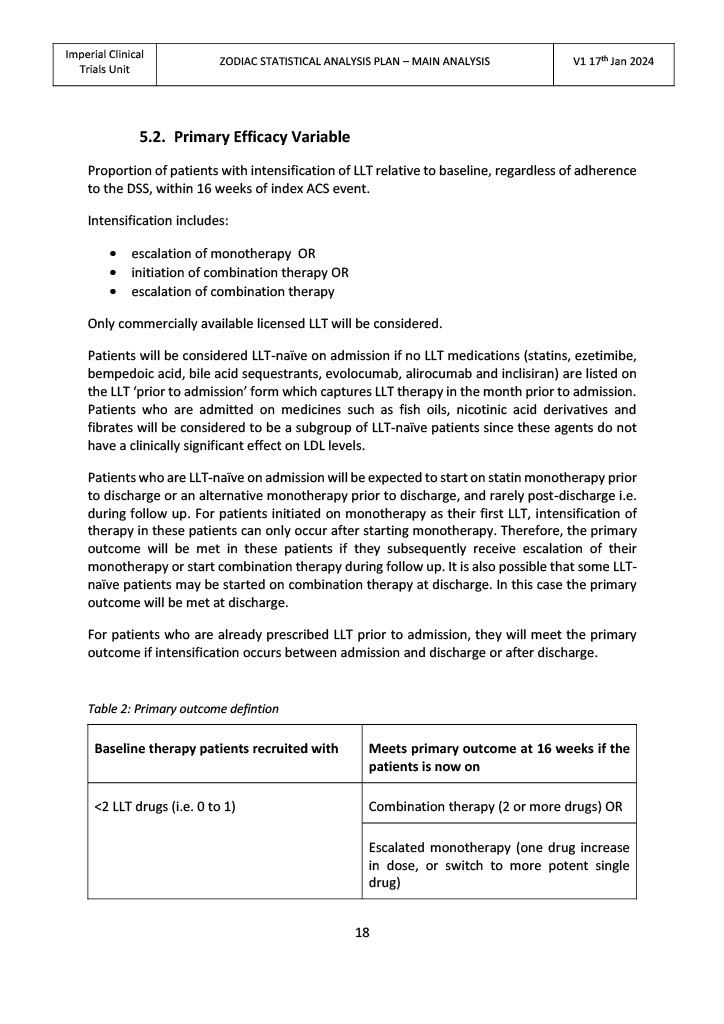

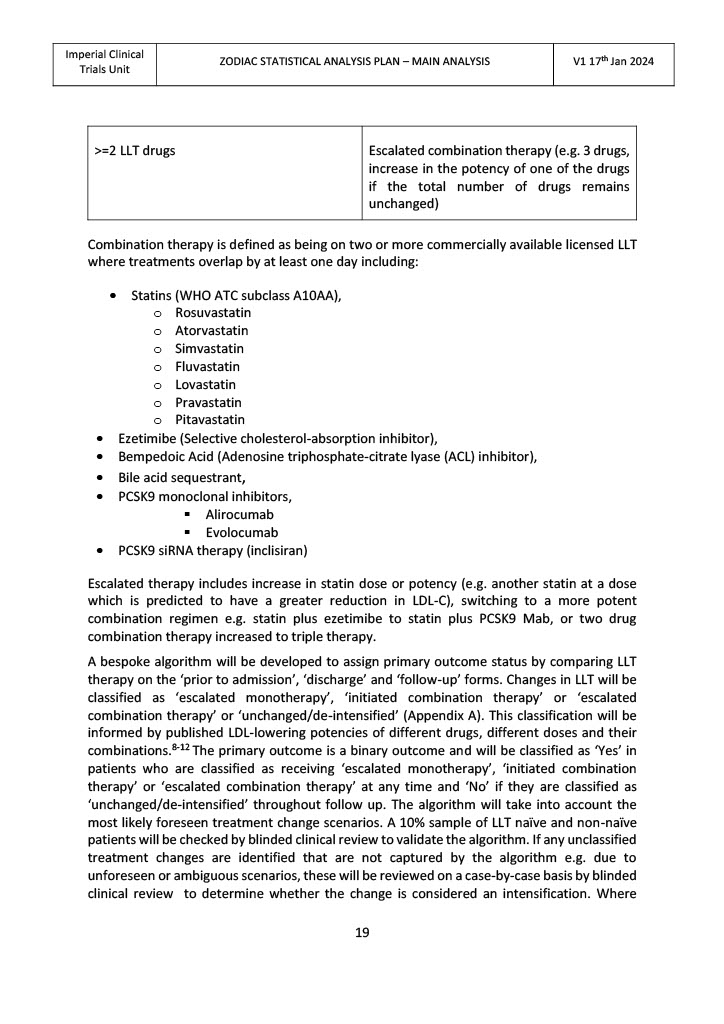

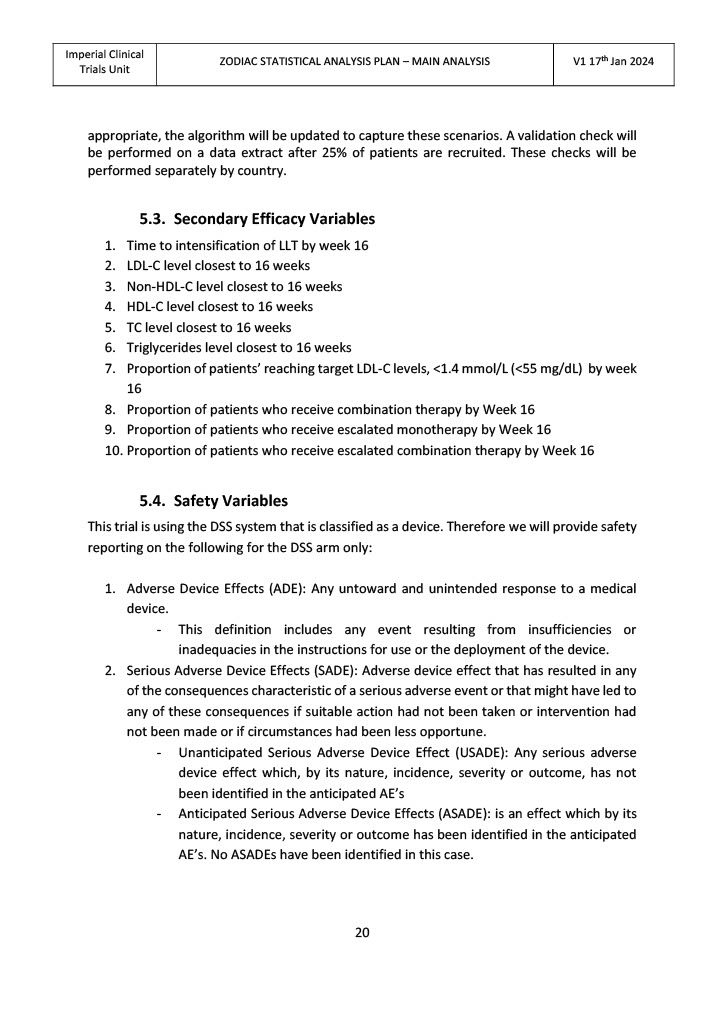

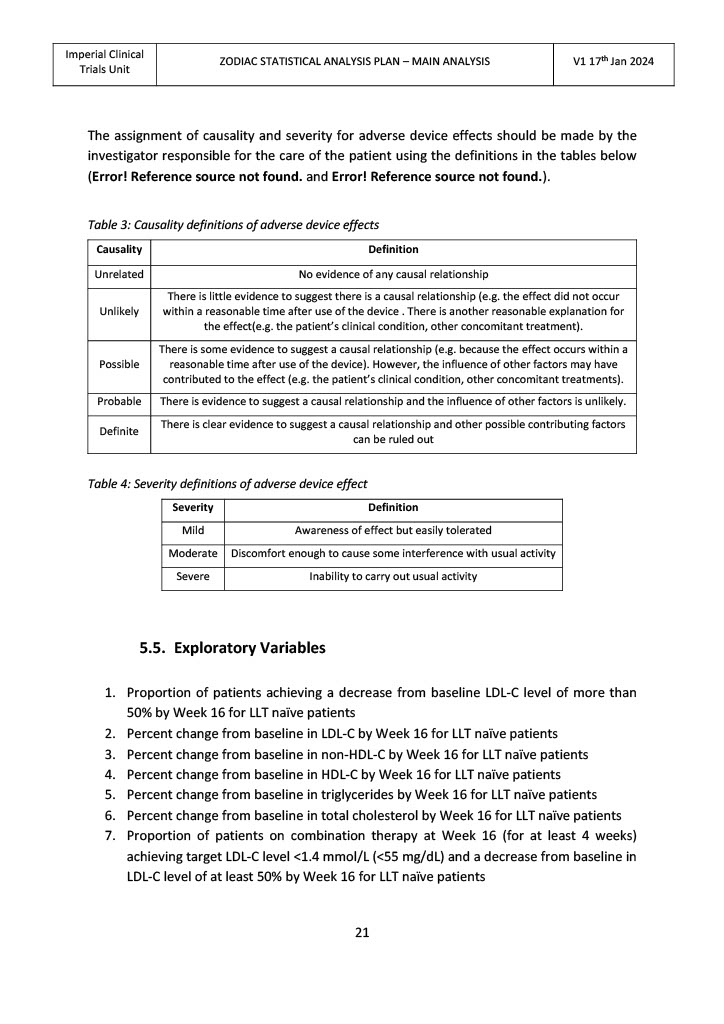

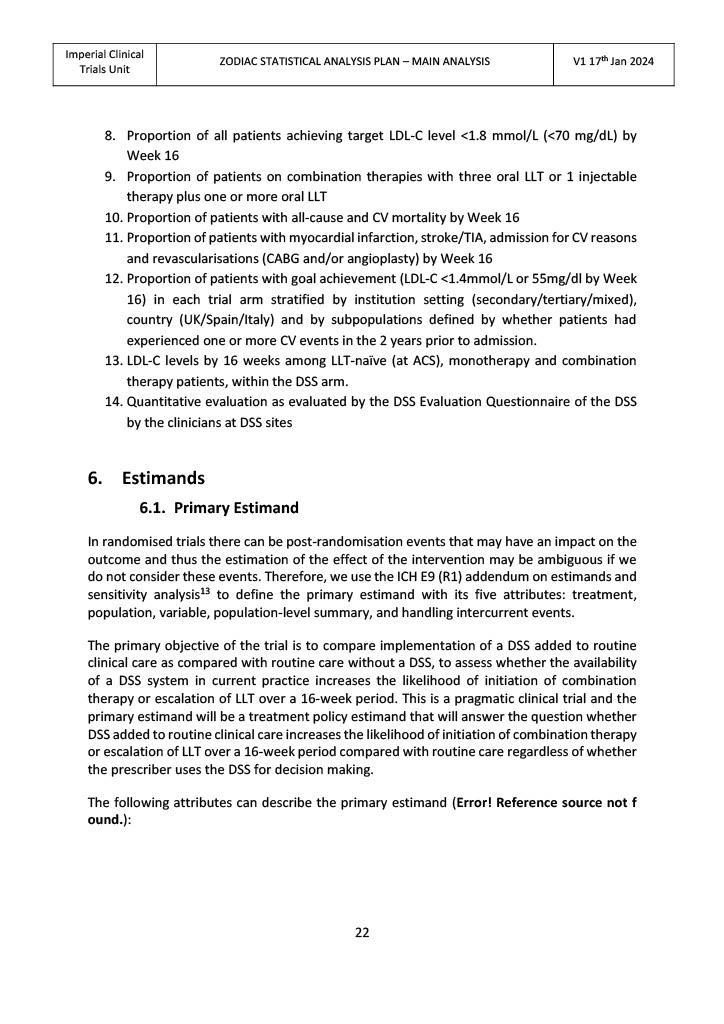

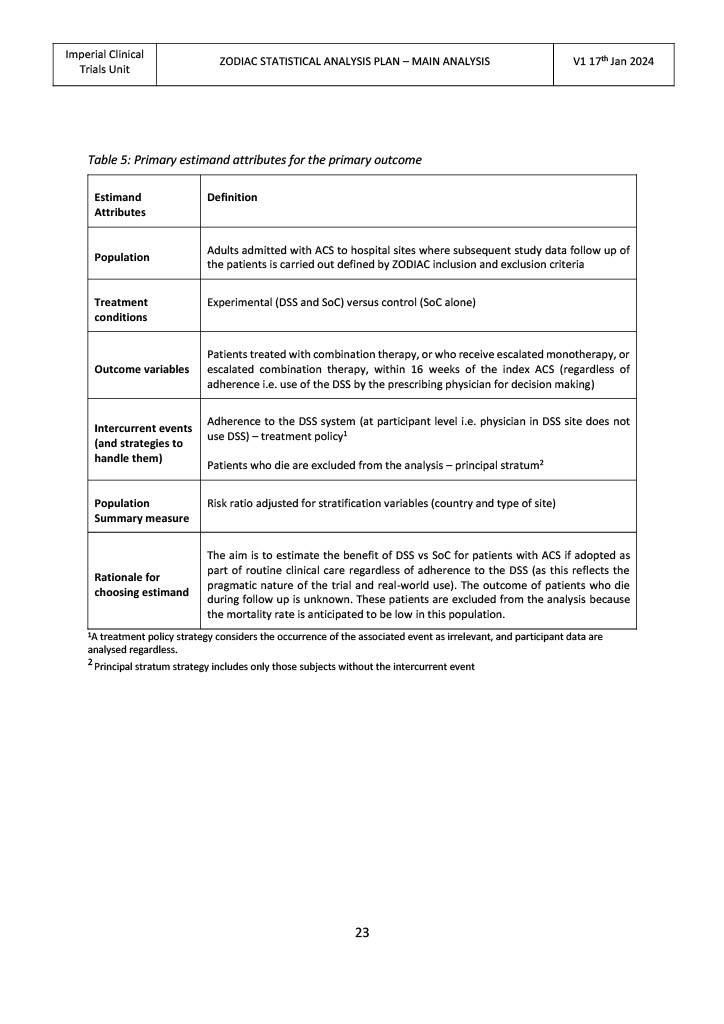

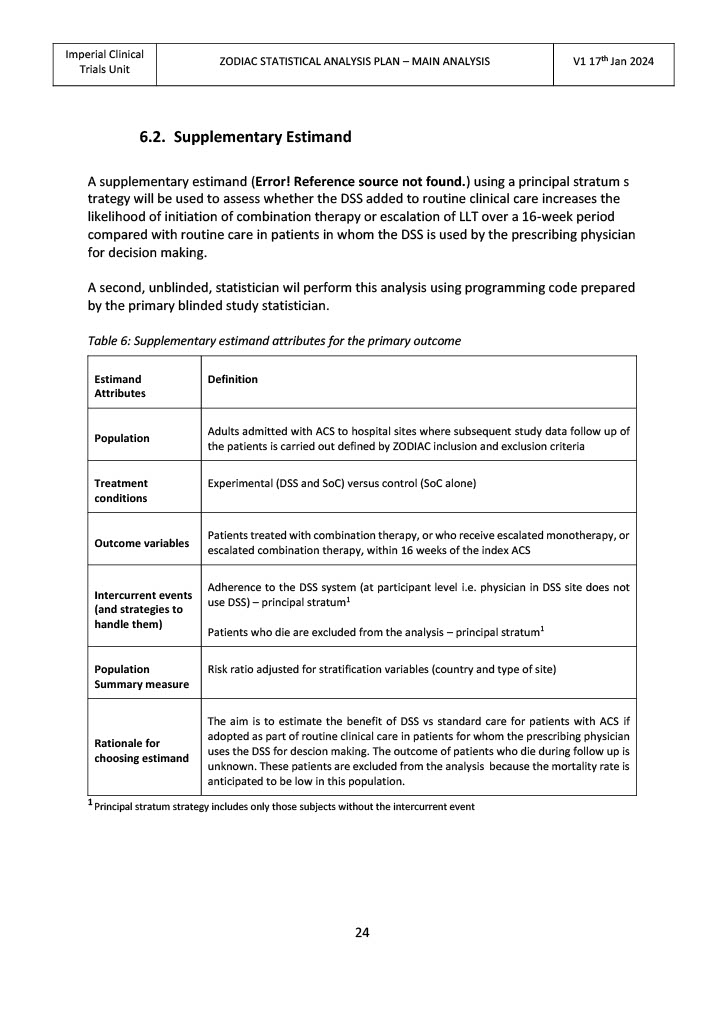

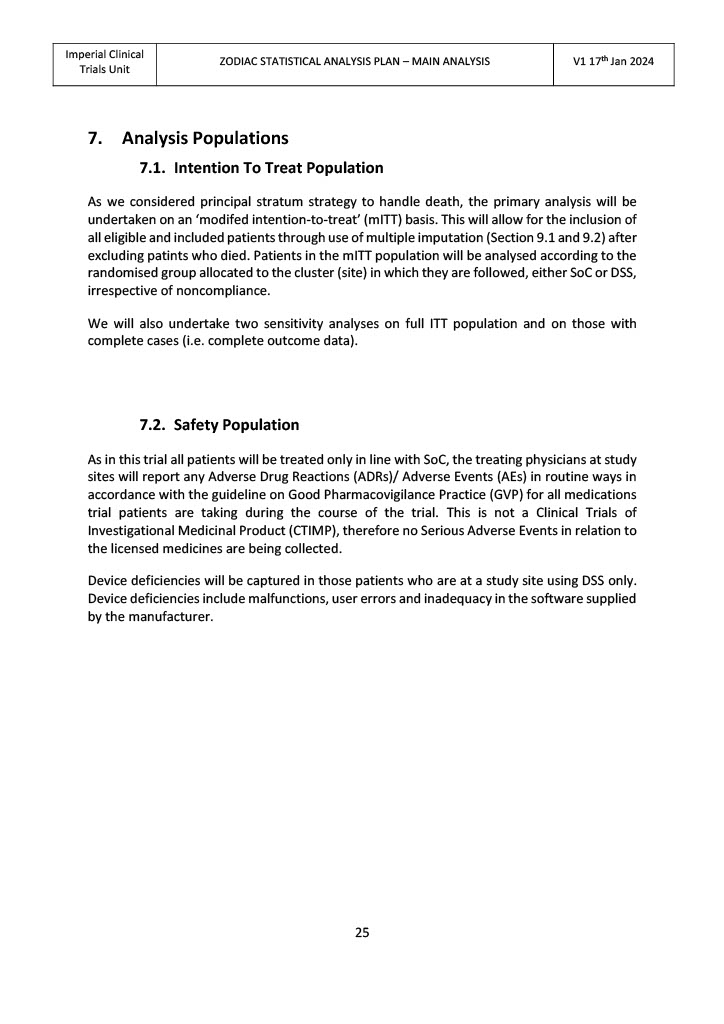

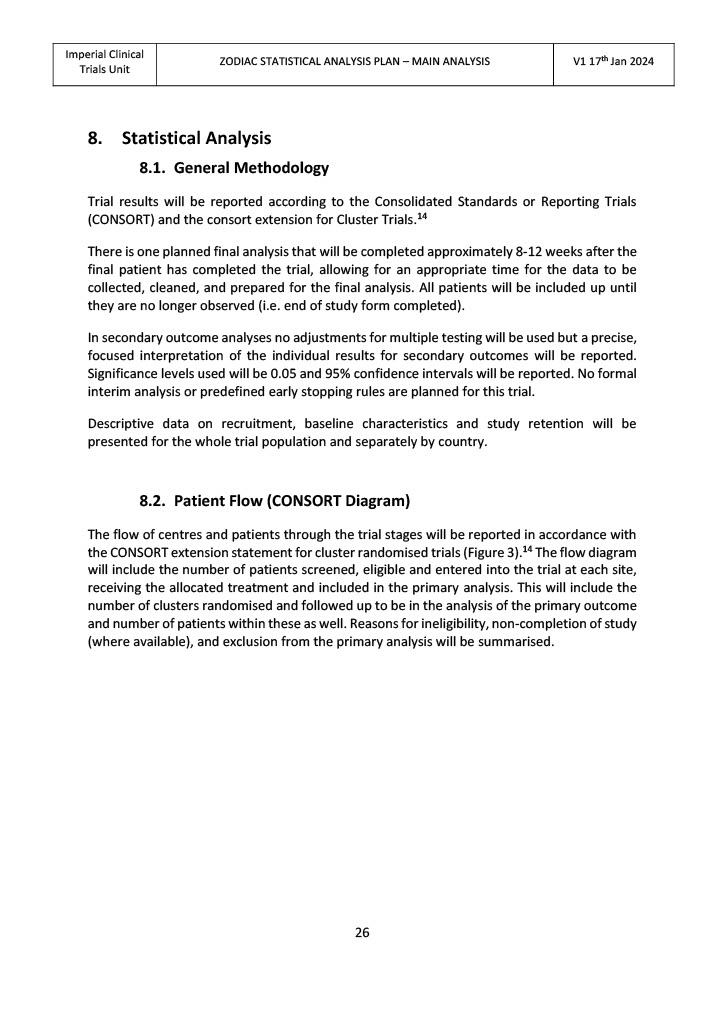

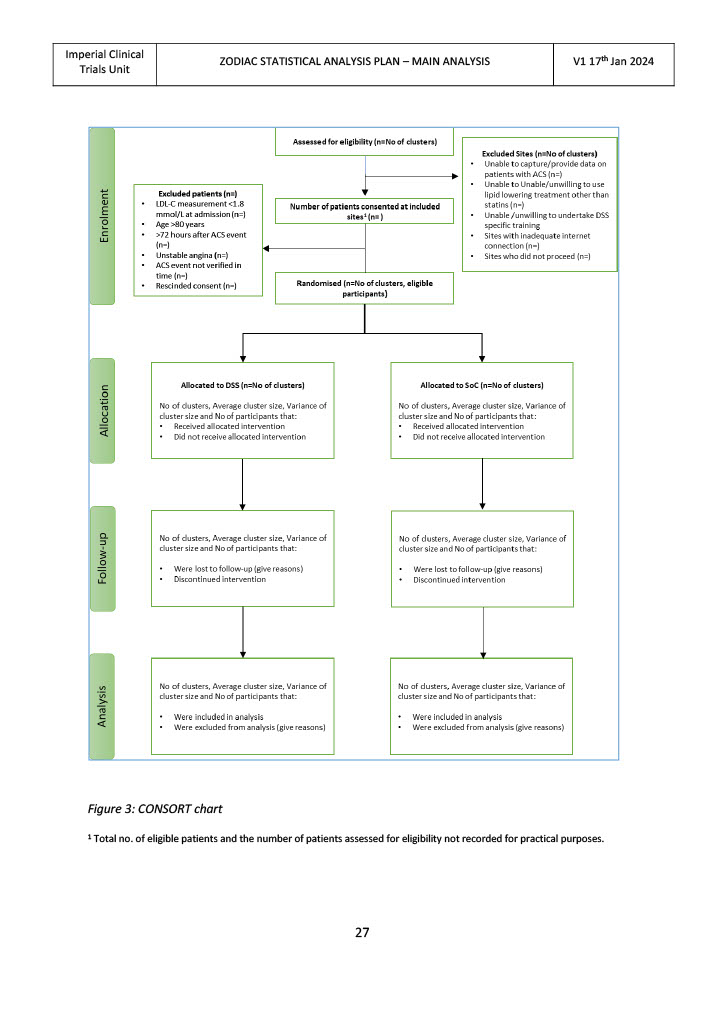

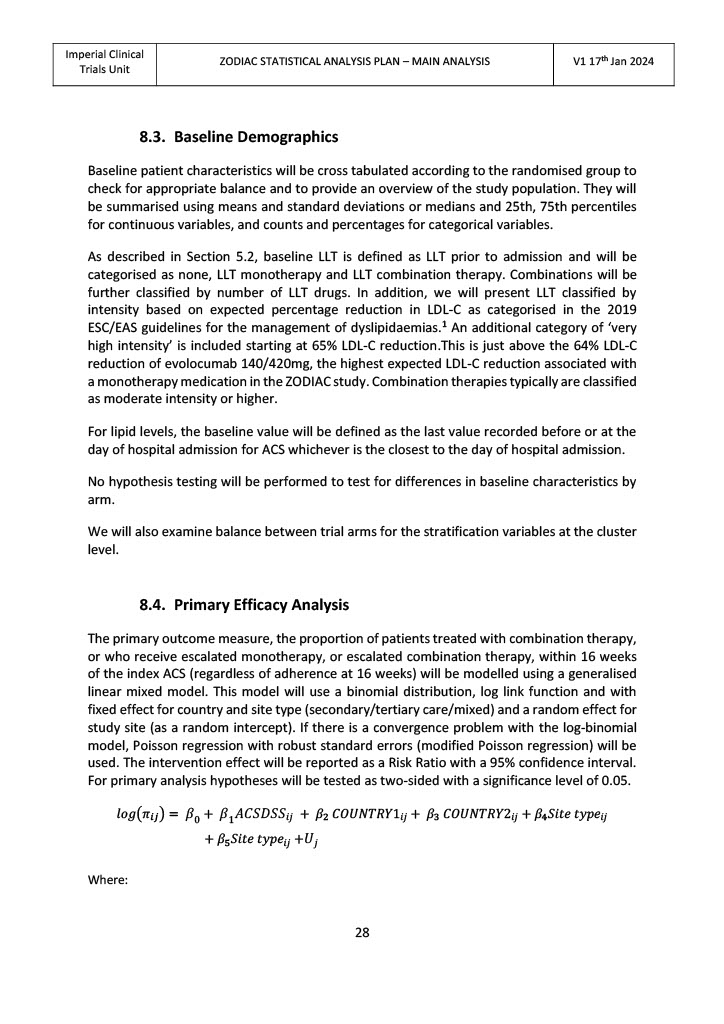

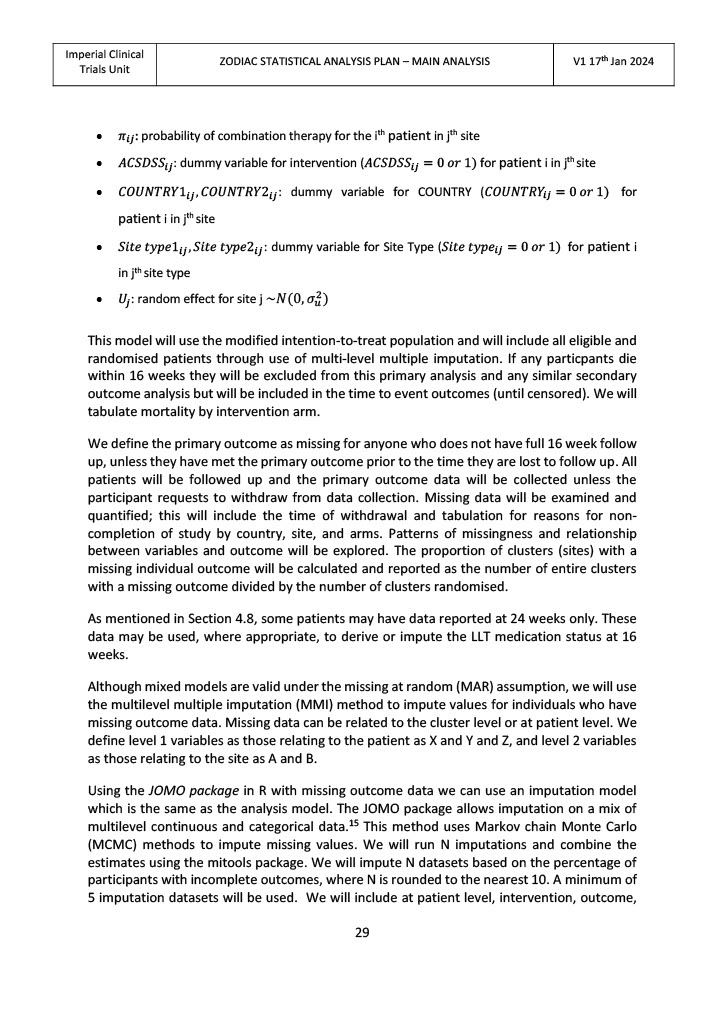

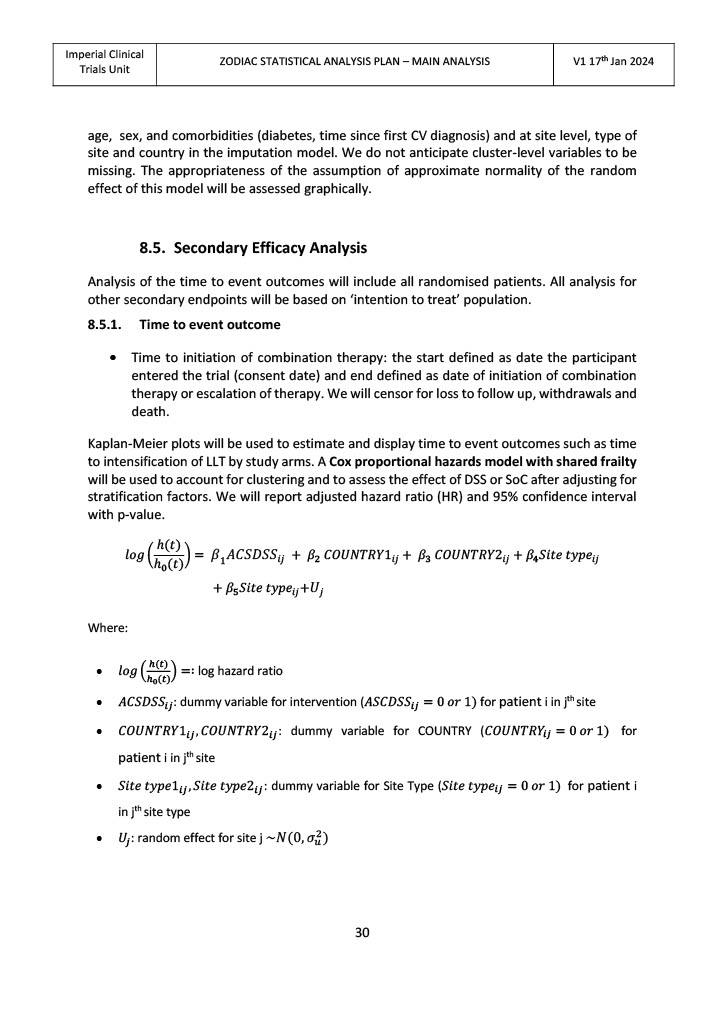

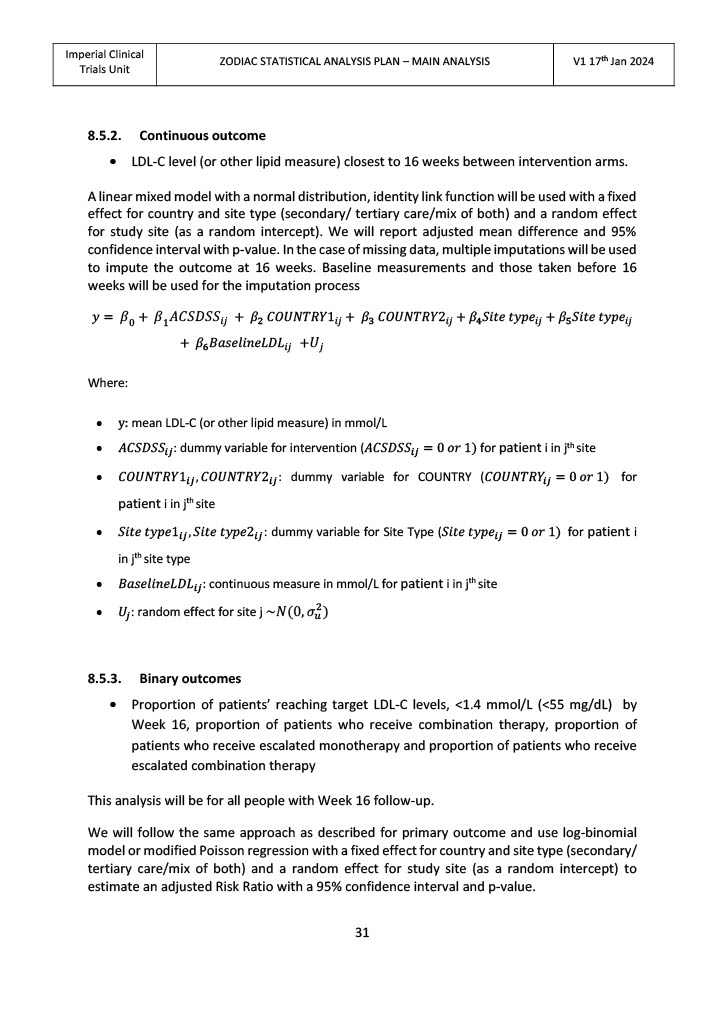

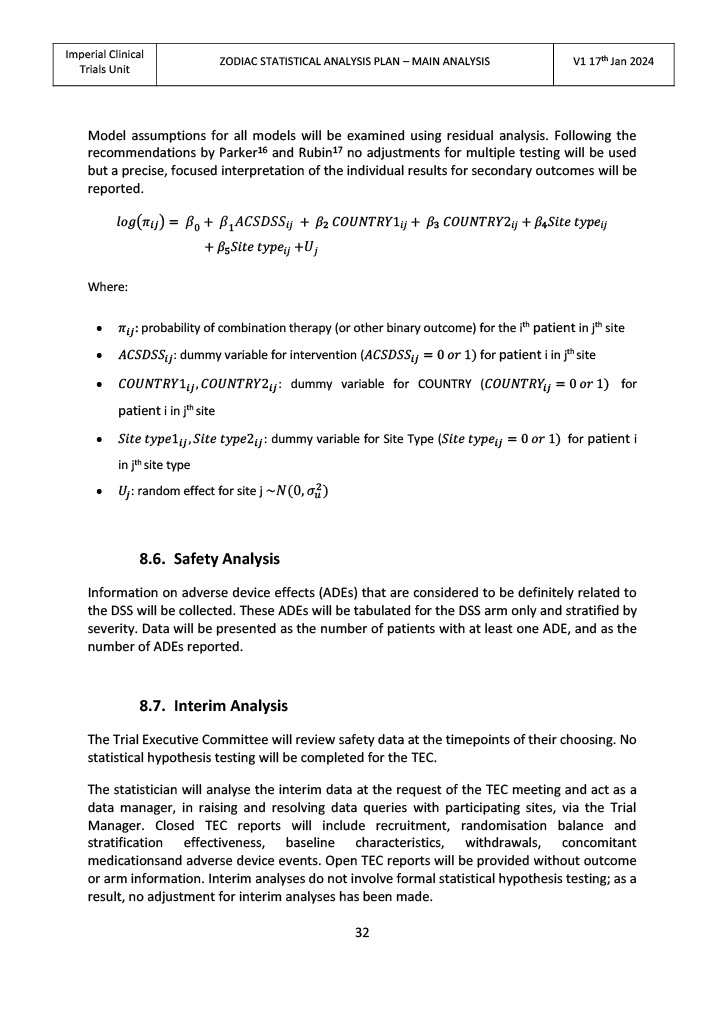

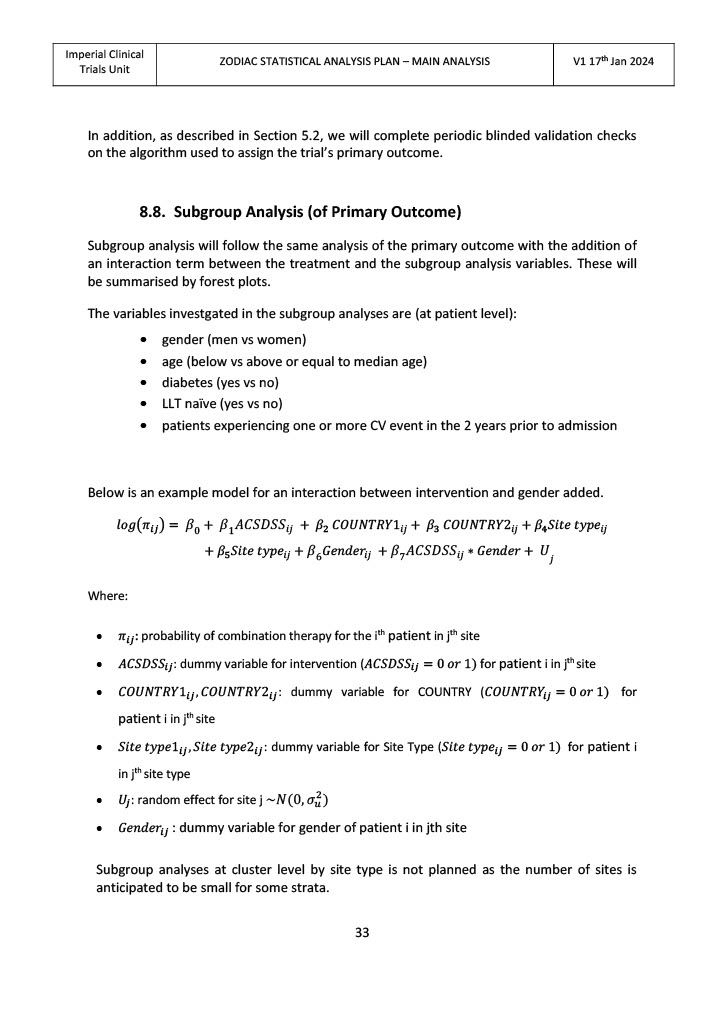

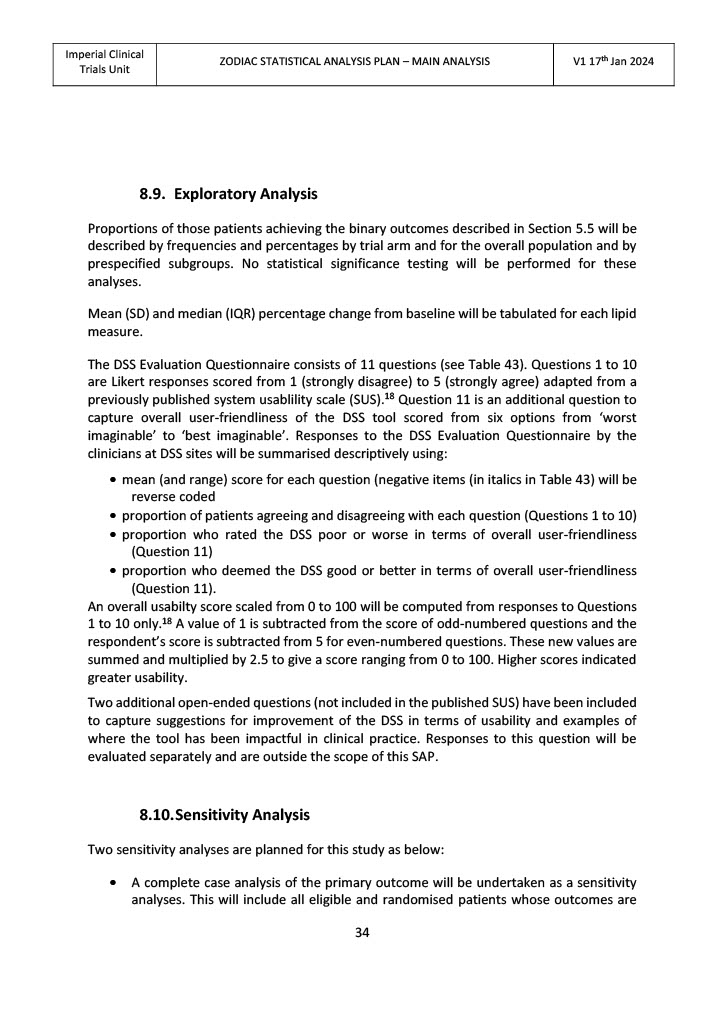

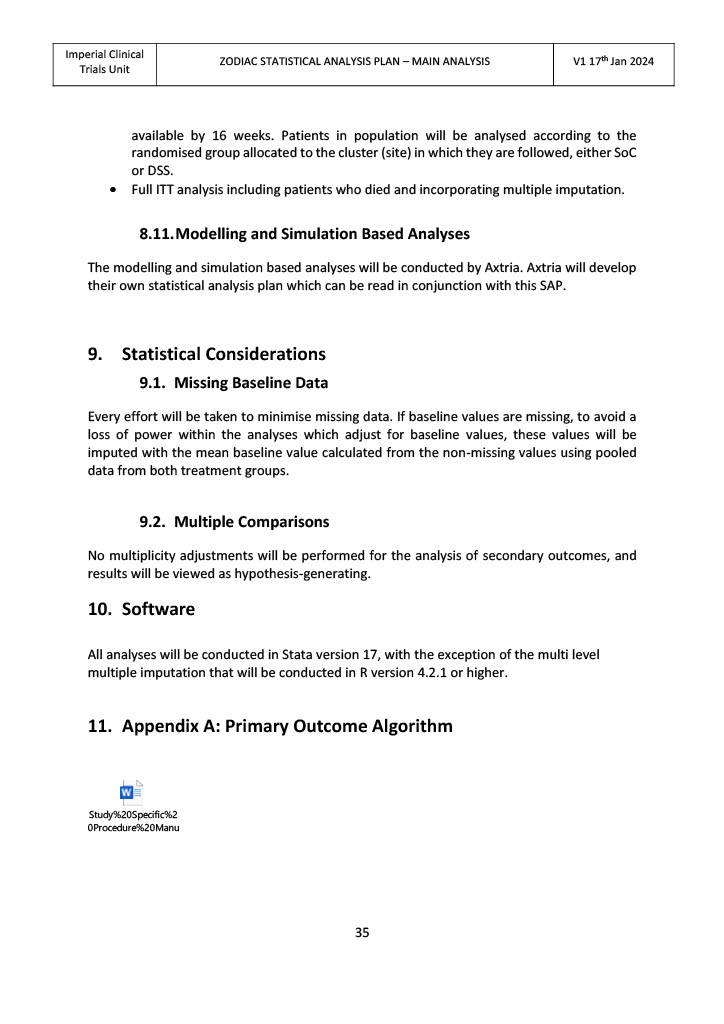

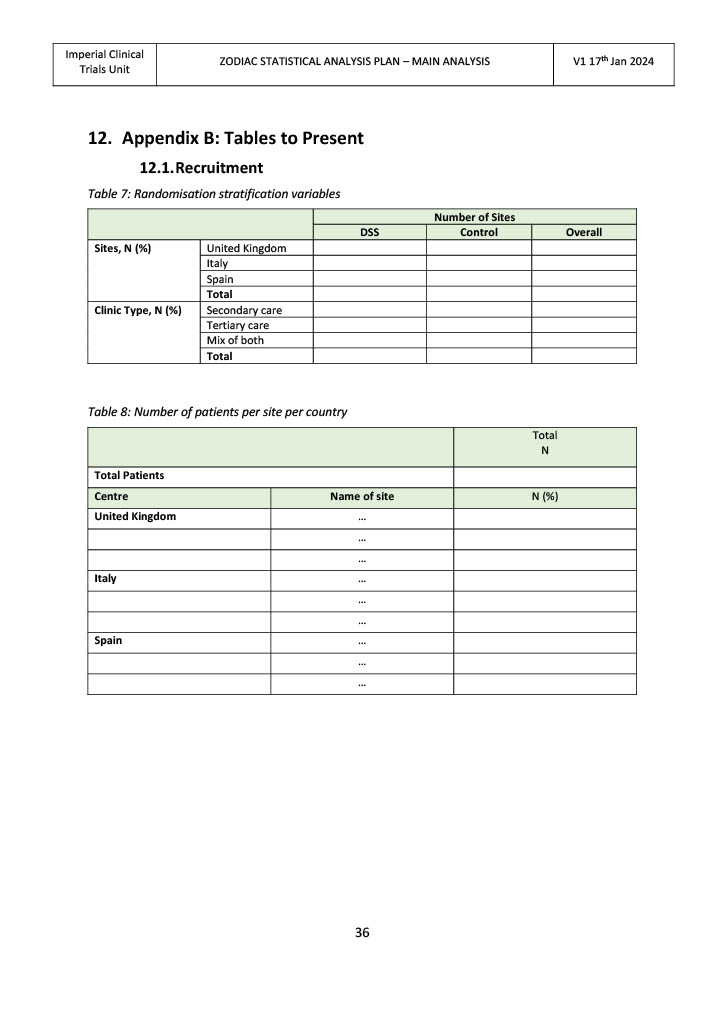

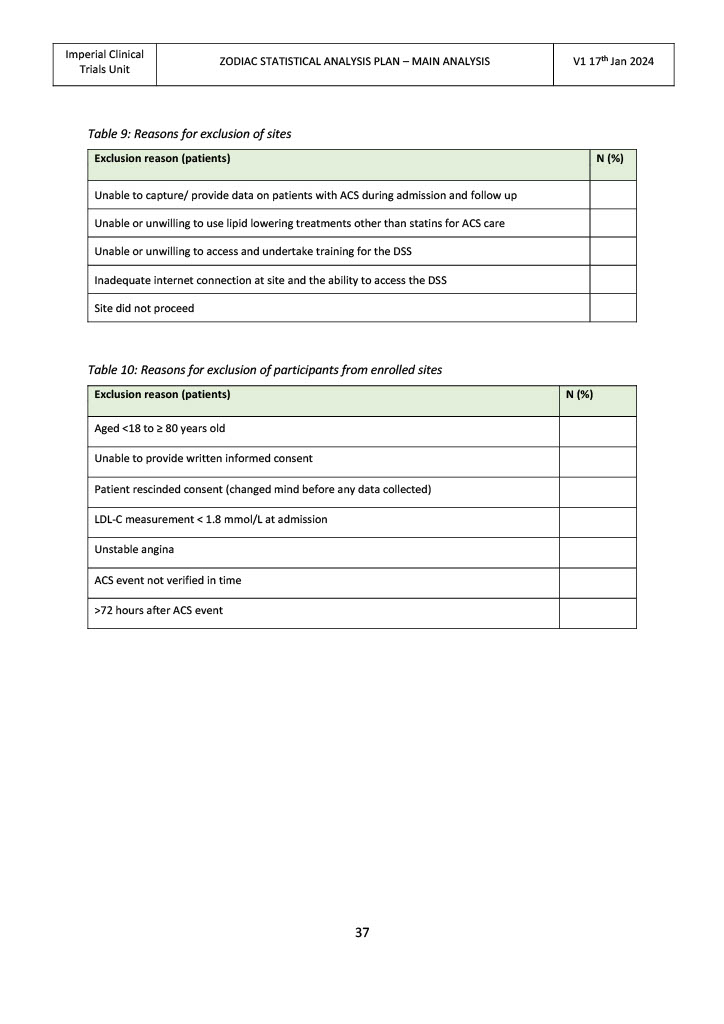

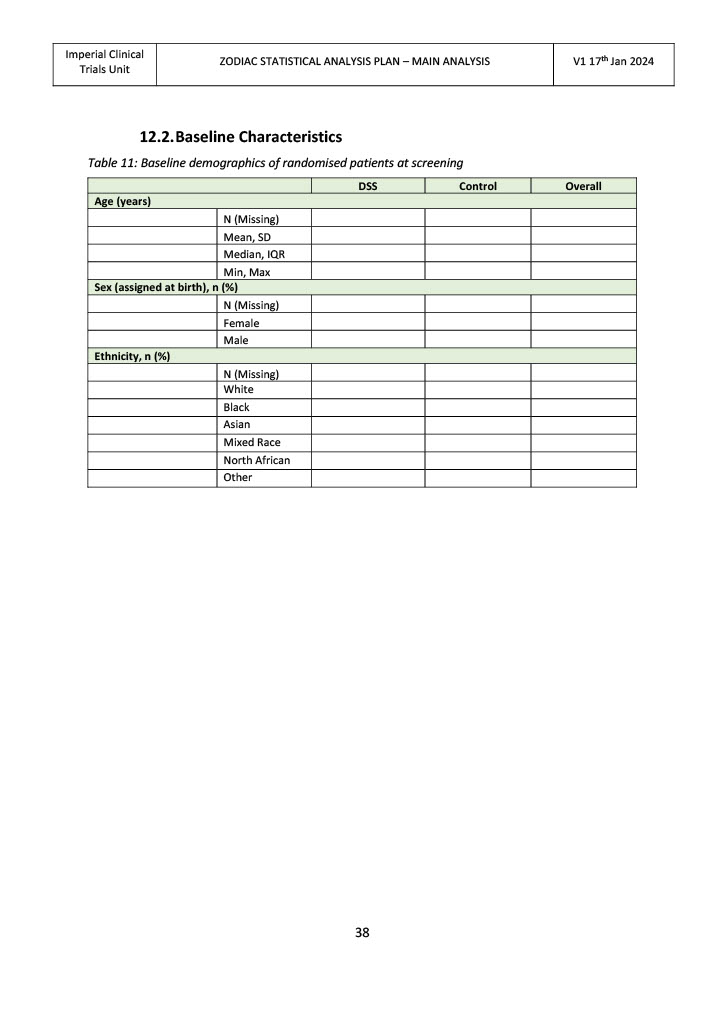

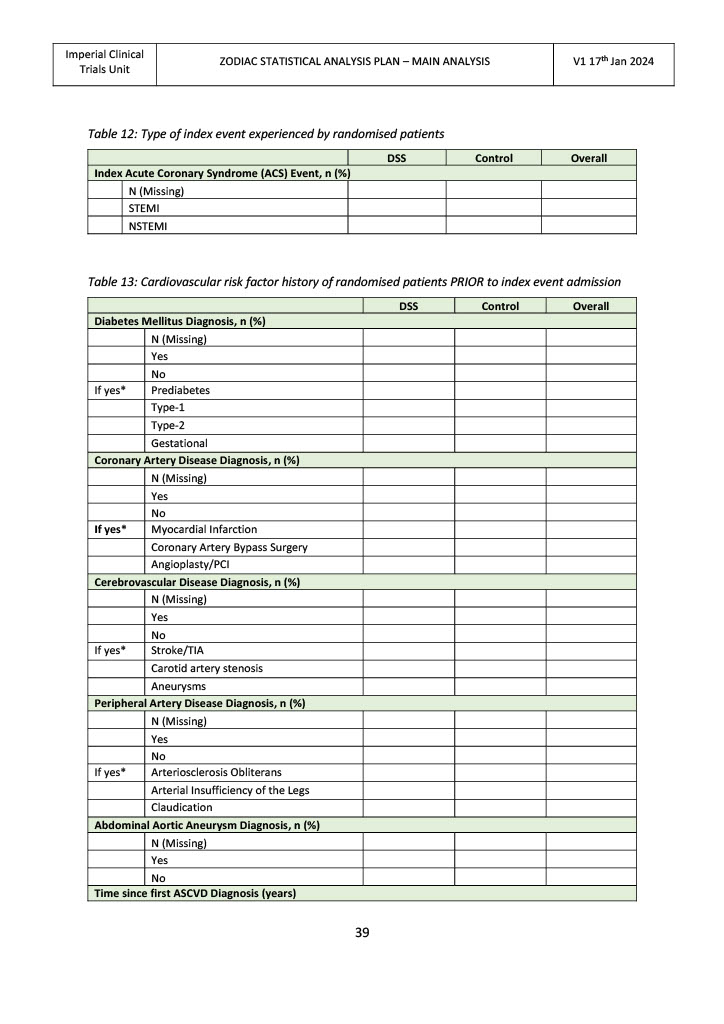

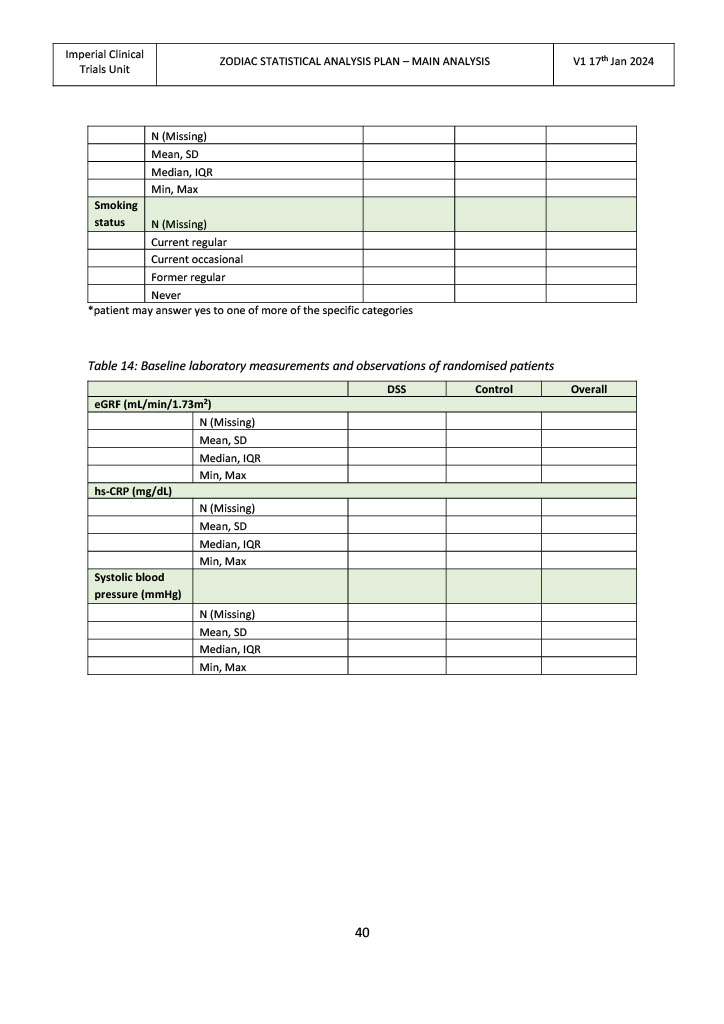

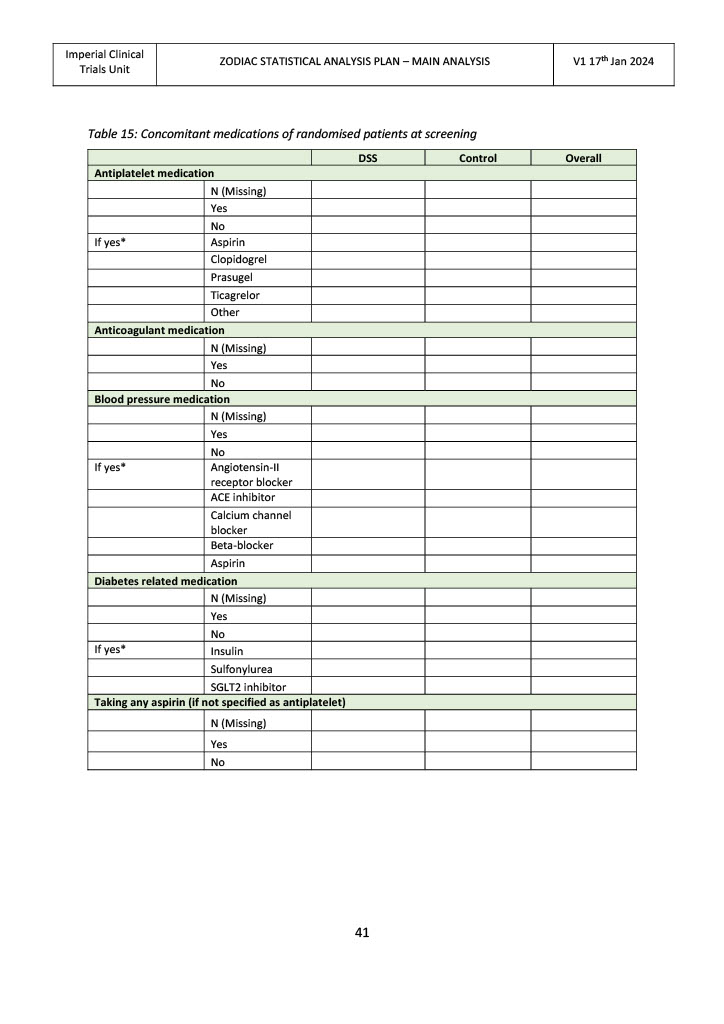

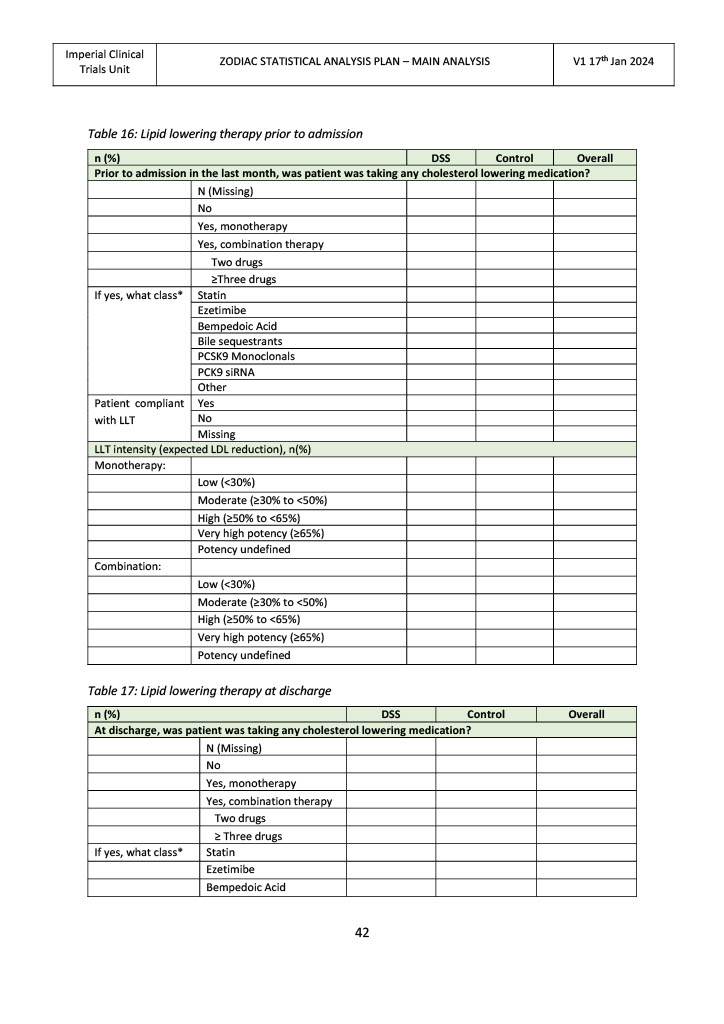

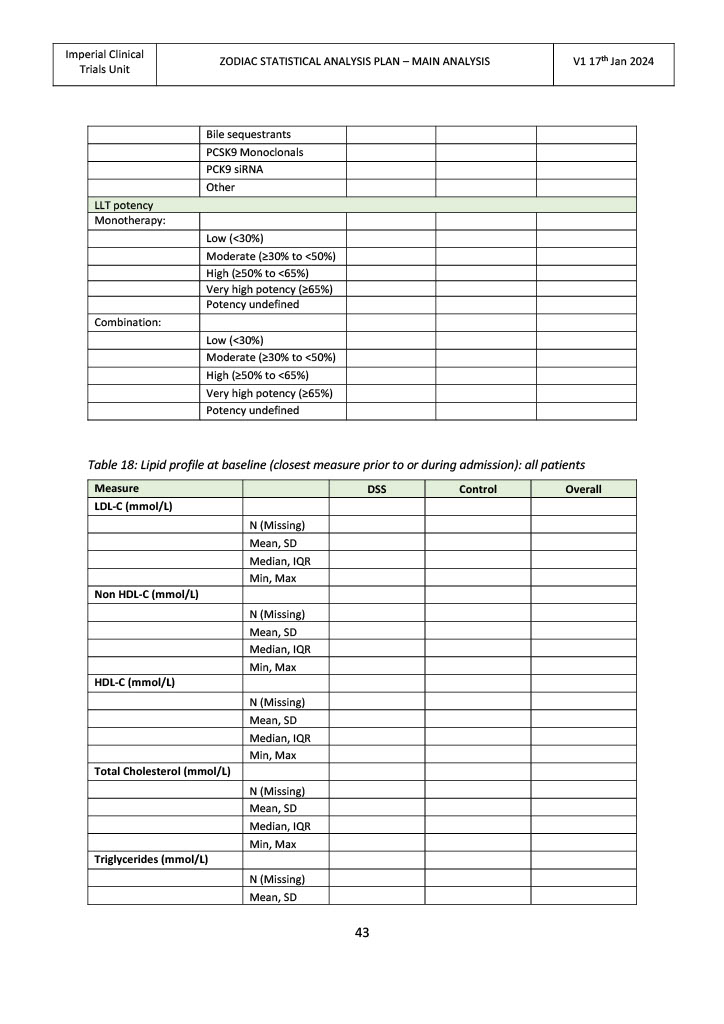

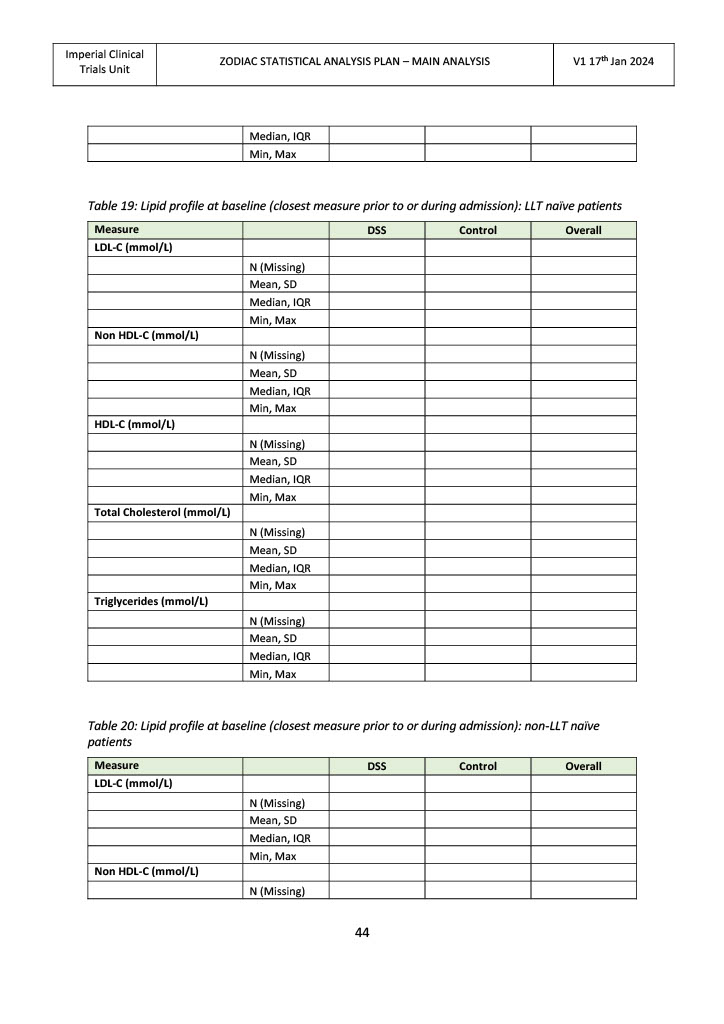

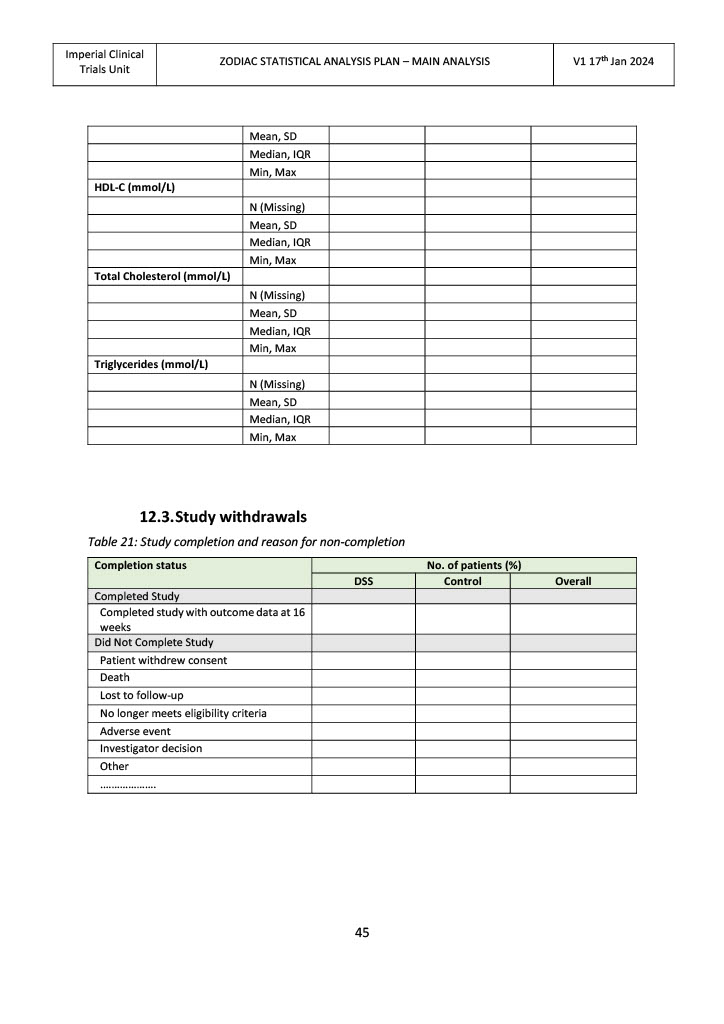

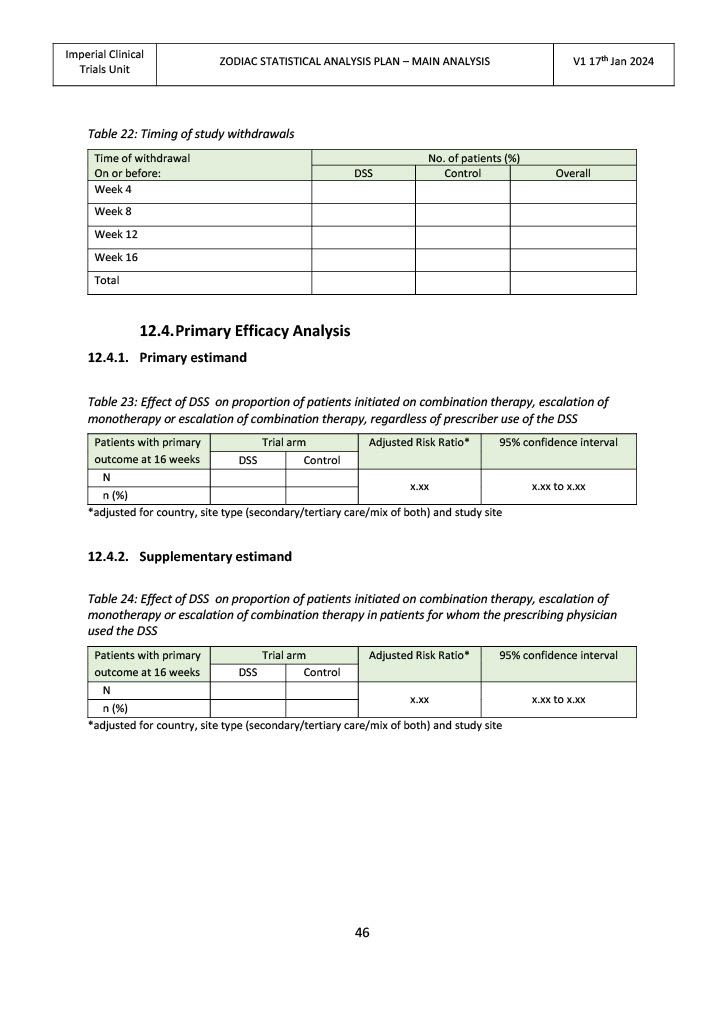

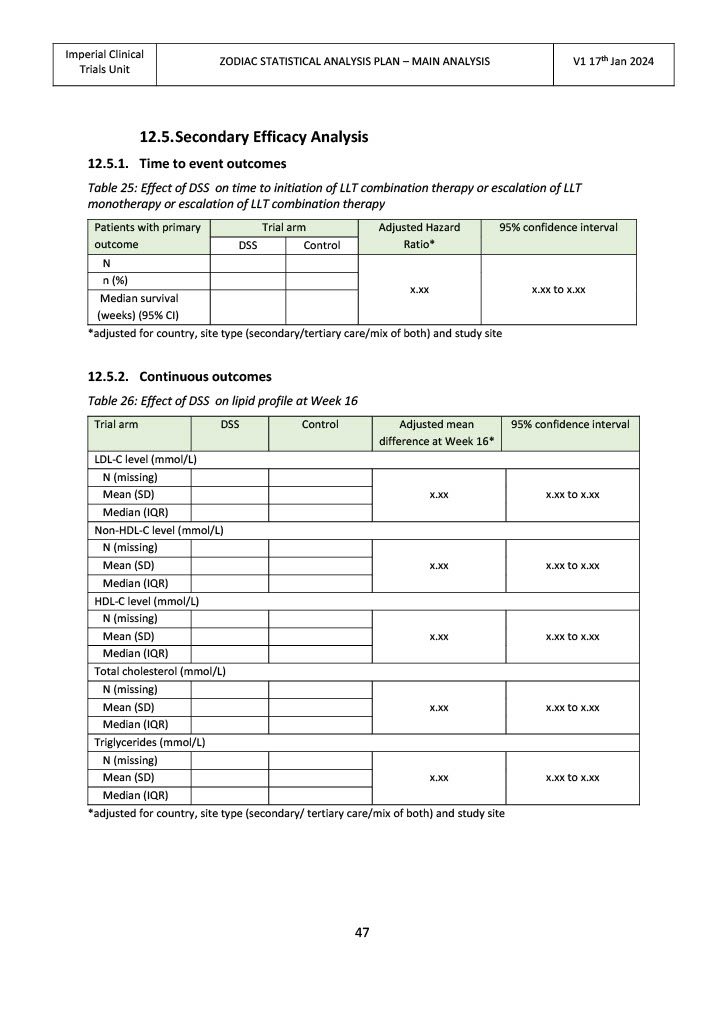

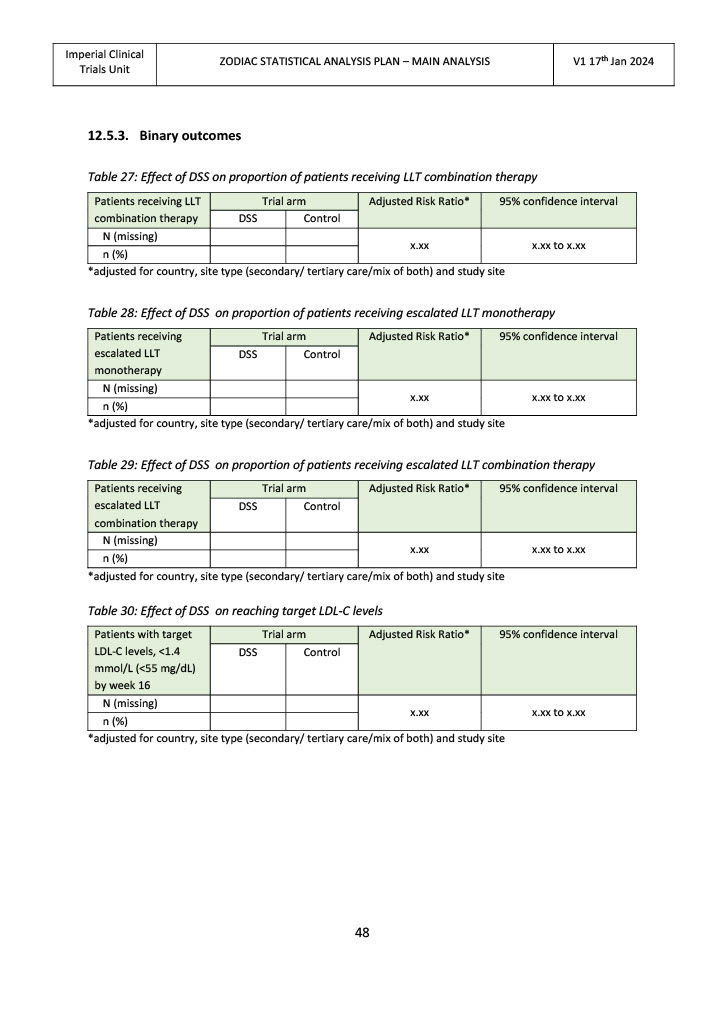

Supplement: ztaf135_Supplementary_Data [file ztaf135_supplementary_data.docx]
